# Influence of chloride on the crystallization and photostability of mixed-halide FAPbBr_3−*x*_Cl_*x*_ perovskites for photovoltaics

**DOI:** 10.1039/d6el00097e

**Published:** 2026-08-04

**Authors:** Laura Bellini, Guus J. W. Aalbers, Simon V. Quiroz Monnens, Nick R. M. Schipper, Willemijn H. M. Remmerswaal, Lana M. Kessels, Christ H. L. Weijtens, Martijn M. Wienk, René A. J. Janssen

**Affiliations:** a Materials to Optoelectronic Devices, Institute of Complex Molecular Systems Eindhoven University of Technology P.O. Box 513 5600 MB Eindhoven The Netherlands r.a.j.janssen@tue.nl; b Dutch Institute for Fundamental Energy Research De Zaale 20 5612 AJ Eindhoven The Netherlands

## Abstract

Widening the bandgap of bromide-based metal-halide perovskites by incorporating chloride allows for tunable absorption onsets in the 2.3–3.0 eV range. While mixed Br/Cl perovskites have been extensively studied for light-emitting diodes and photodetectors, their photovoltaic performance remains mostly unexplored. Here, formamidinium lead halide (FAPbBr_3−*x*_Cl_*x*_) perovskites, with *x* = 0.0–2.4, are studied to understand how chloride incorporation shapes structural, optoelectronic, and photovoltaic properties. Chloride is fully incorporated, as evidenced by near-linear bandgap increase and lattice contraction, but it profoundly alters the crystallization process. Higher chloride concentrations retard the nucleation, leading to larger grains with more irregular shapes and increased grain merging. The mixed Br/Cl perovskites experience light-induced halide segregation, with faster initial segregation kinetics at higher chloride fractions. The bromide- and chloride-rich domains remix in the dark with time. For *x* = 0.6, solar cells benefit from incorporating small amounts of cesium, which reduces hysteresis and significantly improves light stability. Top passivation by 4-fluorophenethylammonium chloride to suppress interfacial recombination, combined with a ternary-fullerene blend electron-transport layer (PCBM, CMC, ICBA), raises the open-circuit voltage (*V*_OC_) to 1.66 V and increases the power conversion efficiency (PCE) from 5.5% to 7.3% at an optical bandgap of 2.38 eV. Unlike lower-bandgap perovskites where losses are dominated by the electron-selective interface, mixed Br/Cl perovskites show comparable recombination losses at the electron- and hole-selective interfaces. At higher chloride fractions (*x* > 0.6), nonradiative recombination increases and the open-circuit voltage does not improve despite the widening of the bandgap, limiting device performance. PCEs of 5.1% and 3.0% are obtained for *x* = 1.2 and 1.8, making optimization of the perovskite/transport-layer interfaces essential for such wide-bandgap absorbers.

## Abstract 2

Chloride widens the bandgap of FAPbBr_3−*x*_Cl*_x_* perovskites and retards crystallization, resulting in larger grains but reduced light stability. Cs improves photostability, while F-PEACl raises the efficiency to 7.3% at a 2.38 eV optical bandgap.

## Graphical abstract

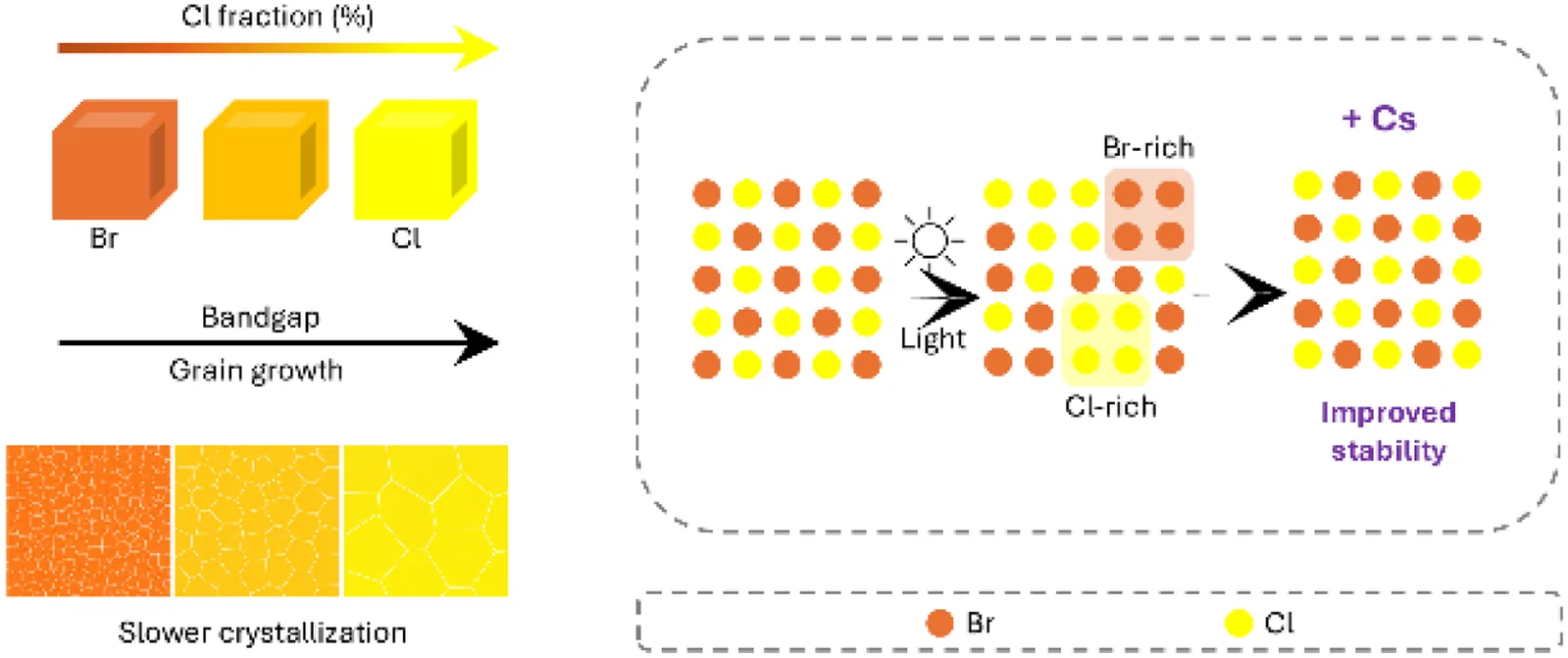

**Broader context**

Wide-bandgap perovskite semiconductors are attracting increasing interest for applications that require either high photovoltage or partial visible transparency, including multijunction photovoltaics, indoor or building-integrated photovoltaics, and photoelectrochemical water splitting systems. Achieving these wider bandgaps through halide alloying, however, introduces fundamental materials challenges. In mixed-halide perovskites, compositional tuning is frequently accompanied by increased defect densities, interfacial energetic losses, and light-induced halide redistribution, all of which limit device efficiency and operational stability. While these issues have been extensively investigated in iodide/bromide perovskites, they remain comparatively unexplored for bromide/chloride perovskite photovoltaics despite offering access to the ultra-wide-bandgap regime. As a result, the relationships between chloride incorporation, crystallization behavior, halide dynamics, and photovoltaic losses are still poorly understood. This work shows that chloride does not only widen the bandgap, but fundamentally changes the formation and operational behavior of the perovskite absorber. Additionally, in ultra-wide-bandgap bromide/chloride perovskites, interfacial recombination losses become a dominant limitation. This emphasizes the need for charge-transport layers and interface engineering strategies specifically designed for these highly energetic absorbers rather than adapted from lower-bandgap perovskite solar cells.

## Introduction

The bandgap of lead-halide perovskites can be tuned by varying the halide composition, making them highly attractive absorber materials for photovoltaic applications.^1,2^ Iodide-based perovskites exhibit bandgaps around 1.5 eV and deliver the highest single-junction efficiencies, reaching 27.3% according to the latest certified records.^3,4^ Substitution of iodide by bromide increases the bandgap up to 2.3 eV, enabling wide-bandgap perovskites for multijunction photovoltaics, indoor photovoltaics (IPV), building-integrated photovoltaics (BIPV), and light-driven water splitting.^5–9^ Increasing the bandgap is expected to yield a higher open-circuit voltage (*V*_OC_), while inherently reducing the short-circuit current density (*J*_SC_) due to the narrower absorption range.^10^ In practice, however, wide-bandgap perovskites suffer from additional efficiency losses compared to mid-bandgap compositions. These are predominantly caused by enhanced nonradiative recombination and interfacial energy-level misalignment.^11,12^ At elevated bromide contents (typically >20% with respect to the total halide fraction), an additional performance-limiting phenomenon emerges in mixed I/Br perovskites, which undergo light-induced halide segregation driven by lattice strain and iodide-vacancy-mediated ion migration, resulting in the formation of iodide-rich, narrow-bandgap domains under illumination.^13–15^ These domains act as recombination and charge-funneling centers, further degrading photovoltaic performance. Modeling of mixed-halide I/Br solar cells has shown that the open-circuit voltage is highly sensitive to the fraction of segregated minority phases, with a strongly nonlinear *V*_OC_ loss and a decreasing tolerance for phase segregation as the bandgap (*i.e.* Br fraction) increases.^16^ Considerable effort has been devoted to mitigating halide segregation in these materials. For instance, the incorporation of cesium as an A-site cation has been shown to reduce lattice mismatch and suppress halide mobility,^17–19^ while bulk and surface passivation strategies aim to reduce defect densities, such as halide vacancies, thereby limiting ion migration and improving operational stability.^20–22^ Despite these advances, light-induced halide segregation remains a major challenge for wide-bandgap I/Br perovskites.

Further bandgap widening beyond the I/Br regime can be achieved by incorporating chloride. Br/Cl perovskites result in bandgaps up to 3.0 eV and have been intensively studied for perovskite-based light-emitting diodes and photodetectors,^23–25^ yet their application in solar cells remains limited. These ultra-wide-bandgap materials may be of interest for semi-transparent photovoltaic and BIPV applications because of their reduced absorption in the visible spectral range. Like mixed I/Br, light-induced halide segregation has been reported in few Br/Cl perovskites studies.^22^ However, in-depth studies of the thermodynamics and kinetics of segregation, the underlying mechanisms, and their effects on photovoltaic performance remain limited. To date, the most detailed investigation was conducted by Cho and Kamat,^26^ who quantified the activation energy barriers for photosegregation and dark recovery by monitoring temperature-dependent absorption of MAPb(Br_0.5_Cl_0.5_)_3_ (MA is methylammonium) perovskites under illumination. Higher energy barriers and light intensity thresholds for segregation were observed compared to I/Br perovskites, indicating greater intrinsic phase stability in Br/Cl materials. Despite this potential advantage, photovoltaic devices based on Br/Cl perovskites remain scarce. According to the annual survey of emerging photovoltaic materials by Almora *et al.*,^10^ the highest reported efficiencies were achieved by Zuo *et al.*,^27^ who explored chloride contents up to 33% relative to the to the total halide concentration. Here, the most efficient chloride-containing device exhibited a power conversion efficiency (PCE) of 8.22% for a composition with 11% Cl. The highest *V*_OC_, 1.55 V, was obtained for a composition containing 19% Cl, remaining far below the radiative voltage limit of approximately 2.02 V at its bandgap of 2.36 eV.^28^ Further increases in chloride content led to a rapid deterioration of all photovoltaic parameters. While mixed Br/Cl perovskite solar cells with chloride contents above 33% remain unexplored, Zia *et al.* recently reported *V*_OC_ values as high as 1.78 V in full-chloride MAPbCl_3_ solar cells.^29^ However, the extremely wide bandgap of MAPbCl_3_ (3.03 eV) severely limits visible light absorption and photocurrent generation, resulting in PCE < 1%. Despite the promising optoelectronic properties of Br/Cl perovskites, their photovoltaic performance and stability have yet to be comprehensively investigated.

In this work, we fabricate FAPbBr_3−*x*_Cl_*x*_ (FA is formamidinium) perovskite films with chloride contents up to 80% within the halide sublattice. Through combined structural, optical, and photovoltaic characterization, we explore how chloride incorporation influences bandgap tuning, crystal lattice parameters, electronic structure, and film morphology, as well as its impact on crystallization dynamics. We further investigate the emergence of light-induced halide segregation and its potential consequences for device stability in these wide-bandgap absorbers. To address these challenges, strategies such as partial cesium incorporation, top surface passivation, and electron-transport layer blending are employed to improve device stability and mitigate interfacial recombination losses.

## Results and discussion

### Composition and film properties

**Fig. 1:**
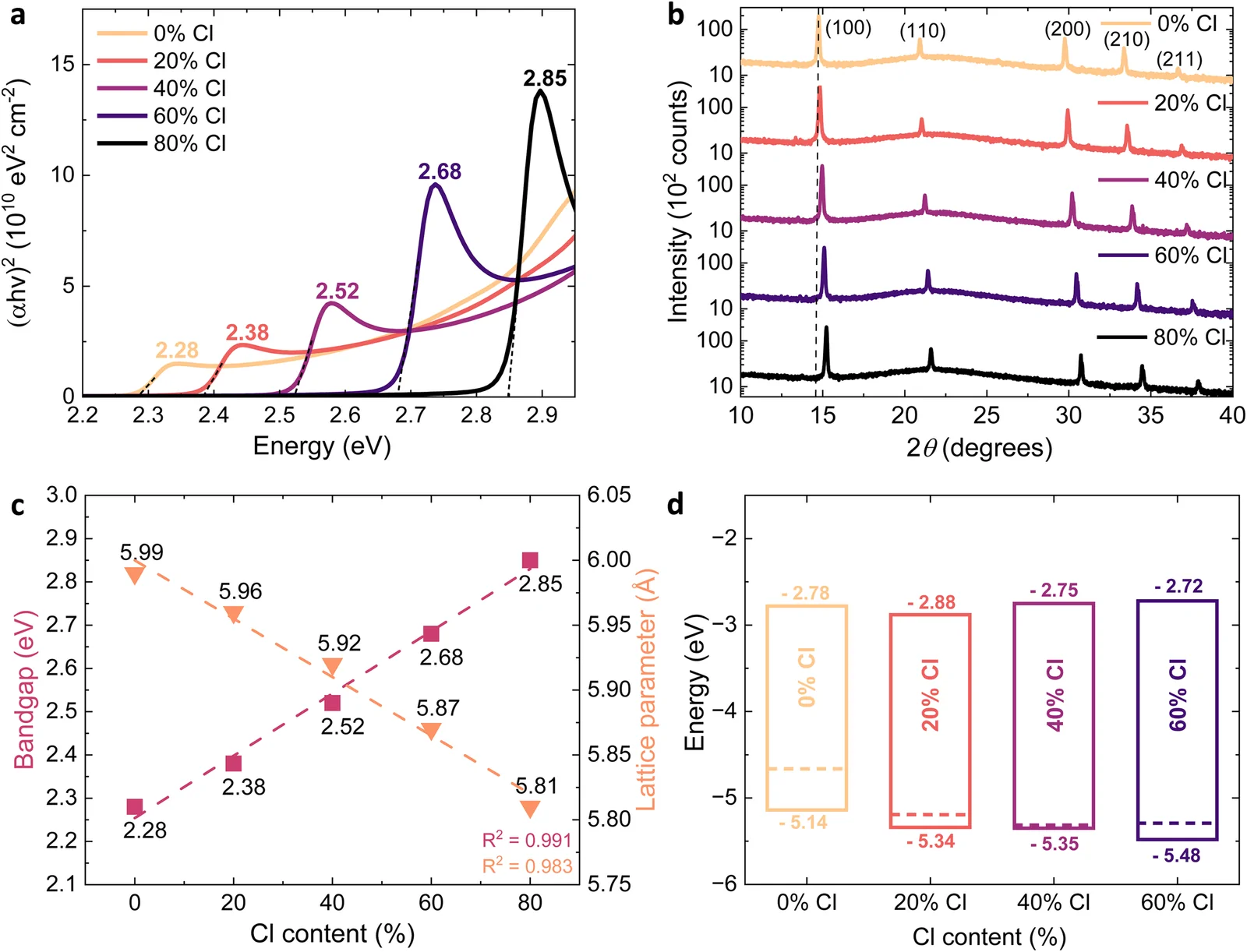
Characterization of FAPbBr_3−*x*_Cl_*x*_ perovskite films containing 0, 20, 40, 60, and 80% Cl relative to the total halide concentration. (a) Tauc analysis from absorbance spectra, (b) XRD, (c) optical bandgaps and lattice parameters determined from Tauc analysis and (100) XRD reflection, (d) VBM and CBM *vs.* vacuum. The dashed line indicates the work function.

Formamidinium lead halide perovskite films with a general FAPbBr_3−*x*_Cl_*x*_ composition were prepared with *x* = 0.0, 0.6, 1.2, 1.8, and 2.4, corresponding to 0, 20, 40, 60, and 80% of Cl relative to the total halide content. The films were fabricated using a two-step spin-coating process, in which the lead halide (PbX_2_, X = Br, Cl) solution was applied first, followed by the formamidinium halide (FAX) solution after 30 s. With increasing chloride content, the films exhibit a pronounced color change (Fig. S1), evolving from dark orange to yellow and becoming nearly transparent at high Cl fractions, indicating a widening of the optical bandgap. Consistently, ultraviolet-visible (UV-vis) absorbance spectra (Fig. S2) show a progressive blue-shift of the absorbance maximum (*λ*_max_), moving from 529 nm for the bromide-pure perovskite to 508, 481, 453, and 428 nm for films containing 20, 40, 60, and 80% Cl, respectively. The corresponding onsets of absorption, or optical bandgaps, determined from Tauc plots (Fig. 1a, c and Table S1), increase linearly from 2.28 eV for FAPbBr_3_ to 2.85 eV for the 80% Cl composition. This trend is in agreement with the well-known influence of halide substitution on the perovskite electronic structure progressing from I to Br to Cl. The valence band maximum (VBM) in lead-halide perovskites is an antibonding combination of Pb 6s and X valence p orbitals, while the conduction band minimum (CBM) is predominantly composed by Pb 6p orbitals, with some admixture of the X p orbitals.^30,31^ The first ionization potential of halogen atoms decreases down Group 17 as the valence electrons are farther from the nucleus. Cl 3p orbitals are lower in energy than the Br 4p and I 5p orbitals. Hence, the VBM shifts downward when replacing I by Br, and Br by Cl, resulting in a gradual widening of the bandgap. The near-linear increase of the optical bandgap, along with the absence of shallow absorbance onsets or secondary peaks, suggests the proportional incorporation of chloride into the lattice.^32,33^ The optical absorbance spectra were analyzed using the Elliott band-fluctuations (EBF) model to determine the bandgap (*E*_g_) and exciton binding energy (*E*_B_) of the mixed-halide perovskites (Fig. S3, S4 and Table S2).^34^ The bandgap extracted from these fits is consistently 0.08 eV higher than the optical bandgap from the Tauc plots (Table 1). The *E*_B_ remains nearly constant at 33.9 ± 0.4 meV for compositions containing 0–60% Cl, while an increase to 40.2 meV is observed for the 80% Cl composition. Lizárraga *et al.* found 33.3 ± 0.5 meV for MAPbBr_3_ at room temperature,^34^ and 52.9 ± 0.2 meV for MAPbCl_3_ using the spectrum reported by Comin *et al.*^35^ Ory *et al.* reported *E*_B_ of 41.2 ± 0.5 meV for FAPbBr_3−*x*_Cl_*x*_ (*x* = 0 to 0.25).^36^ The spread among the reported *E*_B_ values arises from compositional variations (FA *vs.* MA) and from different versions of the Elliott formula used.^37^ For *E*_B_ ≤ 50 meV and under photovoltaic cell operating conditions, full ionization of the exciton population can be expected.^38^

**Table 1:** Bandgap (*E*_g_) values obtained from Tauc plot analysis, Elliot fitting, and EQE spectra

Cl content (%)	*E*_g_ (Tauc plot)	*E*_g_ (Elliot model)	*E*_g_ (EQE derivative)
0	2.28	2.36	2.28^b^
20	2.38	2.46	2.38^a^
40	2.52	2.60	2.52^a^
60	2.68	2.76	2.66^a^
80	2.85	2.93	

a Obtained from the EQE spectra shown in Fig. 5b.

b From ref. 9.

X-ray diffraction (XRD) analysis (Fig. 1b) further confirmed the formation of a mixed-halide perovskite phase across all compositions. All films exhibit diffraction peaks characteristic of a cubic perovskite structure, indexed to the (100), (110), (200), (210), and (211) planes. No additional reflections are observed for the higher chloride contents, confirming the absence of secondary phases. Increasing chloride content induces a gradual shift of peaks toward higher 2*θ* values, consistent with a reduced lattice spacing resulting from substitution of Br by smaller Cl ions in the perovskite lattice. The (100) reflection (Fig. S5) was used to extract the lattice parameter (*a*), which decreases linearly from 5.99 Å for 0% Cl to 5.81 Å for the 80% Cl film (Fig. 1c and Table S3). The linear contraction follows Vegard's law,^39^ providing further evidence of successful halide mixing in the FAPbBr_3−*x*_Cl_*x*_ system.

The experimental Br and Cl contents were quantified by X-ray photoelectron spectroscopy (XPS, Fig. S6 and Table S4) and compared with the theoretical atomic concentrations. As expected, the Br concentration decreases gradually with increasing Cl content, and the Br and Cl atomic fractions closely match the nominal values. Depth-profiling measurements (Fig. S7) performed on films containing 0, 50, and 100% Cl show a near constant halide distribution throughout the perovskite thickness, indicating the absence of significant vertical compositional gradients.

The detailed-balance (Shockley–Queisser) limit of the photovoltaic parameters of FAPbBr_3−*x*_Cl_*x*_ films under AM1.5 G illumination at 298.15 K calculated using the optical bandgaps obtained from Tauc plot analysis are shown in Table S5. Assuming the detailed-balance limit, *V*_OC_^SQ^ increases from 1.94 V for the bromide-based composition to 2.49 V for the film containing 80% Cl. In contrast, *J*_SC_^SQ^ decreases, as expected, from 8.9 to 2.7 mA cm^−2^, due to the progressively increasing optical bandgap. The fill factor (FF^SQ^) remains nearly constant across the composition range. Combined, the PCE^SQ^ decreases from 16.8% to 6.4%. Based on these results, subsequent investigations were limited to chloride contents up to 60%, as higher Cl fractions would lead to severely reduced current densities resulting from absorption shifted predominantly into the UV region.

Understanding how the band edges shift with increasing chloride content is essential for selecting suitable electron- and hole-transport layers, as efficient charge extraction relies on proper interfacial alignment. Ultraviolet photoelectron spectroscopy (UPS, He I, *hν* = 21.22 eV) was used to determine the VBM and Fermi level (*E*_F_) of films containing 0, 20, 40, and 60% Cl deposited on glass/indium tin oxide (ITO) relative to the vacuum level (*E*_vac_ = 0 eV). The position of the Fermi level was obtained from the secondary electron energy cutoff (*E*_SEE_) of the UPS spectrum *via E*_F_ = −|*hν* − *E*_SEE_|. The VBM = |*E*_SEE_ − *E*_onset_| − *hν* was extracted from the low-binding-energy onset (*E*_onset_), determined from the intersection of the extrapolated linear part of the leading edge in a semi-logarithmic plot with the spectral background, which was corrected for the He I satellite contributions (Fig. S8). This approach provides a more accurate determination of the VBM than a linear extrapolation for halide perovskites, where the density of states gradually decreases near the valence band edge.^40^ The CBM was calculated as *E*_CBM_ = *E*_VBM_ + *E*_g_, where *E*_g_ is the bandgap determined using Elliott's equation (Table S2).

The resulting energy diagrams (Fig. 1d) for the FAPbBr_3−*x*_Cl_*x*_ films show that the VBM shifts significantly from −5.14 eV for the pure bromide film to −5.34 eV for 20% Cl, with the CBM correspondingly decreasing from −2.78 to −2.88 eV, and the bandgap widened as a result of chloride incorporation. The lowering of the VBM is consistent with the deeper valence p orbital energy of chloride relative to bromide, which stabilizes the antibonding Pb–halide states forming the VBM. Further increasing the chloride content to 40% and 60% results in additional shifts, with the VBM located at −5.35 and −5.48 eV, respectively. The non-linear evolution of the VBM, characterized by a pronounced shift at low chloride concentrations followed by a more gradual change at higher fractions, has been previously described in the literature.^35,41^

Chloride incorporation alters the morphological properties of the films: the average film thickness (Table S6), measured *via* profilometry, decreases from 415 to 368 nm when the chloride concentration increases from 0% to 60%. This reduction can largely be explained by the lattice constant contraction from *a* = 5.99 to 5.81 Å across the series. For films with an identical molar amount of perovskite, the layer thickness scales with the cube of the lattice parameter, giving an expected reduction from 415 to 378 nm, which is close to the measured value of 368 nm. We note that additional factors such as viscosity changes and crystallization dynamics also play a role. Surface roughness *R*_q_ – calculated as the quadratic mean of the surface height deviations from the mean plane – remains modest across the series (17–28 nm, Table S6).

**Fig. 2:**
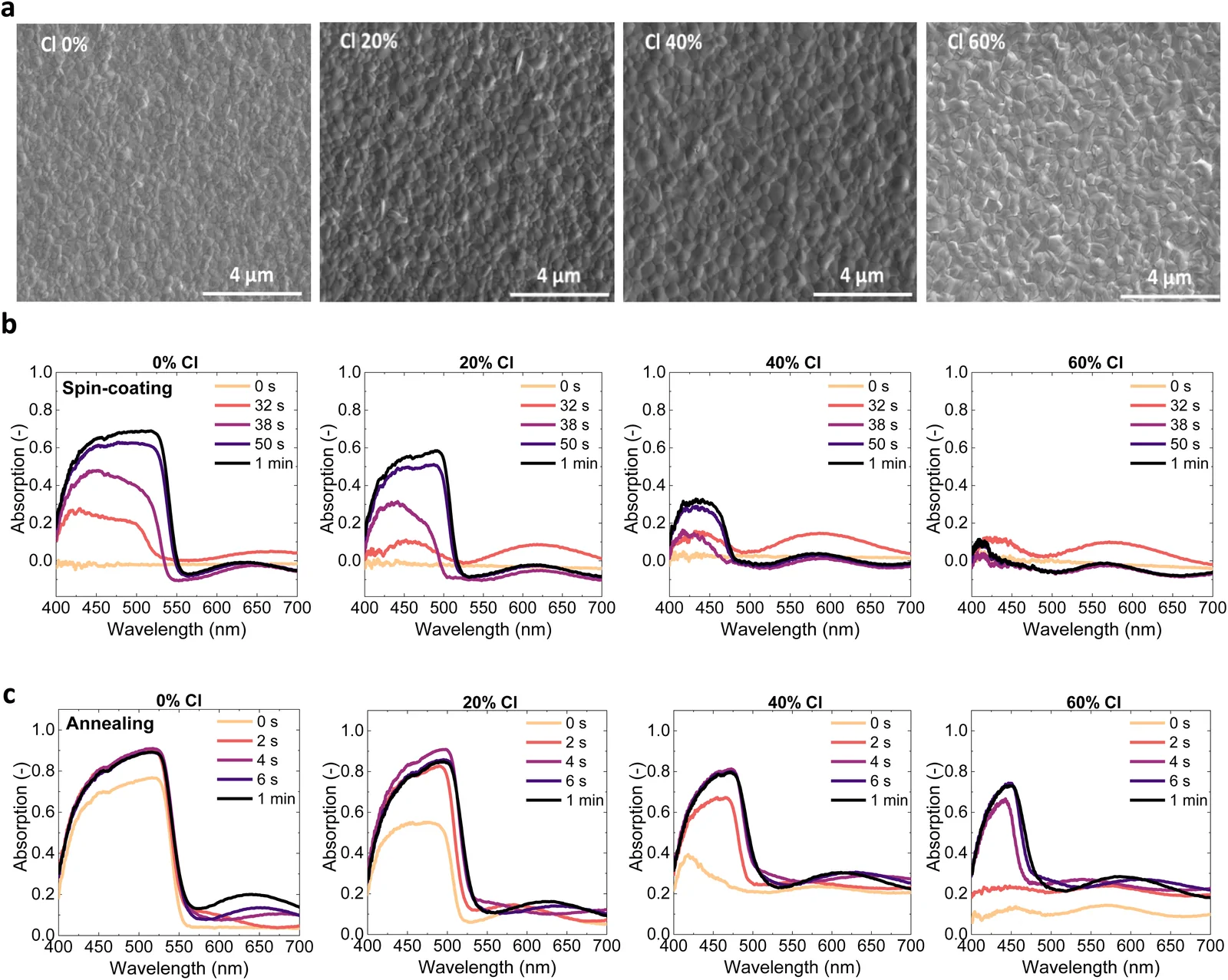
(a) SEM images of FAPbBr_3−*x*_Cl_*x*_ perovskite films containing 0, 20, 40, and 60% Cl relative to the total halide concentration. *In situ* UV-vis absorption spectra recorded during (b) spin-coating and (c) thermal annealing.

Scanning electron microscopy (SEM) images (Fig. 2a) provided further insight into the morphology. All films are continuous, compact layers free of visible pinholes. The bromide-pure film exhibits well-defined, round-shaped grains. With increasing chloride content, the average grain size increases from 323 nm for the full-bromide composition to 403 nm for 20% Cl and 503 nm for 40% Cl (Fig. S9). For the film containing 60% Cl, a comparable average grain size of 470 nm is observed; however, the grain morphology begins to change. The grains appear more irregular in shape and show signs of partial coalescence, consistent with altered grain growth. Similar to the thickness, this behavior is likely related to composition-dependent crystallization dynamics.

Motivated by the effects of the chloride fraction on film thickness and morphology, we investigated the crystallization dynamics using *in situ* UV-vis absorption spectroscopy during both spin-coating (Fig. 2b) and subsequent thermal annealing (Fig. 2c). Absorptance (*A* = 1 − *R*) spectra were determined from the specular and diffuse reflectance spectra (*R*). During spin-coating, spectra were recorded for films containing 0, 20, 40, and 60% Cl prior to deposition (*t* = 0 s) and at selected time points (*t* = 32, 38, 50 s, and 1 min) following the addition of the FAX solution at *t* = 30 s. The 80% Cl composition could not be measured under the same conditions due to insufficient UV light intensity from the halogen lamp used. As expected, at *t* = 0 s the absorption is zero for all samples between 400 and 700 nm. Upon applying the FAX solution at *t* = 30 s, perovskite crystallization can be seen from absorptance at short wavelengths, while weak spectral oscillations appear at longer wavelength resulting from interference effects arising from light reflected from the glass substrate and the wet precursor layer. A pronounced absorption onset is observed for the 0% Cl film at *t* = 32 s, but the appearance of such onset is progressively delayed in time for increasing chloride contents, appearing at *t* = 38 s for 20% Cl, and at *t* = 50 s for 40% Cl. Lastly, even after 1 min, no discernible absorption onset is detected for the 60% Cl film in the accessible wavelength range (>400–1100 nm), suggesting a substantial slowdown of crystallization kinetics with increasing chloride incorporation.

During thermal annealing, spectra were collected at *t* = 0, 2, 4, and 6 s, and after 1 min. In agreement with the spin-coating results, films with lower chloride content show higher initial absorption and a sharper absorption onset from the earliest stages of annealing. At *t* = 0 s, the 0% Cl film appears nearly fully crystallized, followed closely by the 20% Cl composition. The 40% Cl film develops a clear absorption onset after 2 s, whereas for the 60% Cl film the onset becomes evident only after 4 s, highlighting the pronounced delay in crystallization. The delayed crystallization observed for Cl-rich compositions is likely associated with stronger interactions in the precursor solution. Chloride ions act as stronger ligands towards Pb, forming stable complexes in solution and intermediate phases that must first decompose before perovskite formation.^42,43^ For all compositions, the absorption spectra stabilize within approximately 6 s of thermal annealing, and no further changes are observed after 1 min, indicating completion of crystallization. A gradual blue-shift of the absorption edge with increasing chloride content is observed, consistent with the bandgap widening induced by halide substitution. Moreover, the overall absorption intensity decreases with higher chloride content, which correlates with the thinner films revealed by profilometry.

The delayed perovskite crystallization observed for Cl-rich compositions can account for the evolution of film morphology. We speculate that the slower crystallization kinetics with increased Cl content are a consequence of a reduced nucleation rate, leading to fewer initial nuclei that eventually grow into larger grains. At higher chloride fractions, the further suppressed nucleation promotes grain coalescence during growth, giving rise to the increasingly irregular grain shapes and partial merging seen by SEM.

### Recombination and halide dynamics

While halide substitution allows for continuous bandgap tuning in FAPbBr_3−*x*_Cl_*x*_ perovskites, the resulting optoelectronic quality is ultimately governed by recombination losses. To probe the effect of chloride incorporation, we investigated the carrier recombination dynamics using time-resolved photoluminescence (TRPL) and quantified the quasi-Fermi level splitting (QFLS) *via* absolute photoluminescence (APL) at 1-sun equivalent illumination. TRPL probes the temporal evolution of photogenerated carriers following pulsed excitation, providing insight of recombination kinetics,^44,45^ and APL yields the QFLS under steady-state illumination, which represents the internal *V*_OC_.^11,46^ For both TRPL and APL measurements, perovskite films were deposited on glass covered with an 1,6-hexylenediphosphonic acid (HDPA) interlayer to minimize substrate-perovskite interfacial recombination.^47^

**Fig. 3:**
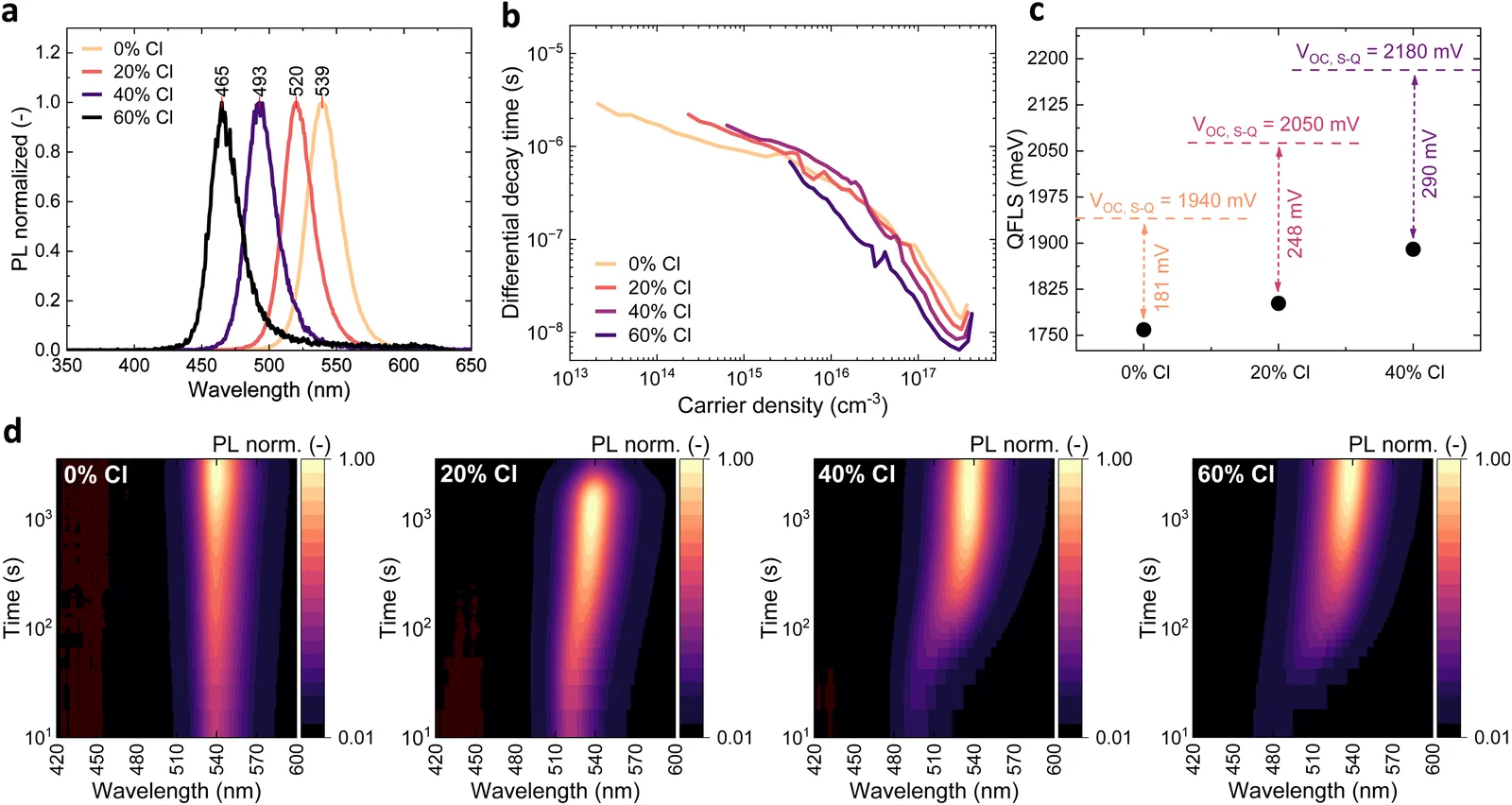
Photoluminescence (PL) of FAPbBr_3−*x*_Cl_*x*_ perovskite films containing 0, 20, 40, and 60% Cl relative to the total halide concentration. (a) Early-time PL emission immediately after (<2 ns) the laser pulse. (b) Differential PL decay time *versus* carrier density. (c) QFLS obtained from absolute PL. (d) Normalized PL emission *versus* time during continuous 365 nm illumination at 1-sun equivalent intensity.

TRPL measurements were performed using a 355 nm pulsed laser, and the PL emission was collected as a function of delay time after excitation. Early-time PL emission spectra recorded immediately after laser excitation (<2 ns) show single emission peaks at 539, 520, and 493 nm for films containing 0, 20, and 40% Cl, respectively (Fig. 3a), consistent with the optical bandgaps obtained from UV-vis spectroscopy. For the film with 60% Cl a PL peak at 465 nm is found that is less symmetric and tails slightly to longer wavelengths. The excitation density under pulsed excitation with a low a time-averaged generation rate (*G* ≈ 5 × 10^18^ cm^−3^ s^−1^) remains low and is unlikely to cause light-induced halide segregation. This is confirmed by the PL emission spectra for 20 and 40% Cl perovskite films. Cho and Kamat demonstrated that electron and hole transfers from mixed Br/Cl to Br-rich domains in mixed Br/Cl perovskites occurs on the timescale of 1–20 ps.^26^ Hence, any light-induced halide segregation formed in the experiment should be visible in our early PL of 20 and 40% Cl films. The weaker additional long-wavelength emission observed for the 60% Cl film suggests the presence of some pre-segregated secondary halide phases.

The TRPL transients (Fig. S10) were converted into differential decay times (*τ*_diff_) and plotted as function of carrier density (*n*) (Fig. 3b) to enable a direct comparison of the PL decay across compositions with different bandgaps. Plotting *τ*_diff_*versus* carrier density, instead of *versus* QFLS, avoids the bandgap-dependent variation in maximum QFLS after laser excitation (Fig. S11) and more clearly highlights differences in recombination behavior between the compositions. At high carrier densities (*n* > 3 ×10^16^ cm^−3^), the chloride-rich films exhibit a faster decay, *i.e.*, a shorter *τ*_diff_. This can be attributed to the increased radiative bimolecular recombination coefficient (*k*_rad_) associated with wider bandgaps (Fig. S12). Simulations using different *k*_rad_ values (Fig. S13) reproduce this behavior, showing uniformly shorter *τ*_diff_ with increasing *k*_rad_ while maintaining nearly identical slopes of *τ*_diff_*versus n* over the entire carrier density range. For lower carrier densities (*n* < 10^16^ cm^−3^) the experiments show a transition from bimolecular to monomolecular-like charge recombination for all samples. For pure monomolecular decay, *τ*_diff_ would be a constant. The experiments show that at lower carrier densities, additional nonradiative recombination processes dominate the recombination decay. Such monomolecular decay is commonly associated with trap-mediated, or Shockley–Read–Hall, recombination.

A 405 nm LED was employed for APL measurements (Fig. 3c). Due to instrument limitations, only films with chloride contents up to 40% could be measured. As expected, the QFLS increases with increasing bandgap, reaching values of 1759, 1802, and 1890 meV for the 0, 20, and 40% Cl films, respectively. However, when compared to the corresponding SQ limits (Table S5), the energetic losses also increased from 181, *via* 248, to 290 meV, respectively, corresponding to 91, 88, and 87% of the SQ limit. Such a trend aligns with literature reports, where wider-bandgap perovskites typically exhibit higher nonradiative recombination losses.^10^ This behavior is often attributed to the increased density of halide vacancies and lattice defects introduced when mixing halides, and to the formation of deeper intrinsic defect states associated with the larger bandgap.^11,12,48^

To assess whether partial chloride incorporation into bromide-based perovskites induces photo-induced halide segregation, samples with varying chloride content were continuously illuminated using a 365 nm LED calibrated to 1-sun intensity, while recording PL spectra over time (Fig. 3d and S14). The pure-bromide sample exhibits a stable PL peak position at 539 nm, as expected for a perovskite incapable of light-induced halide segregation. In contrast, films containing 20, 40, and 60% Cl show a pronounced redshift of the PL over time, evolving from 520, 494, and 469 nm, respectively, to 539 nm, *i.e.*, to the PL wavelength observed for FAPbBr_3_. This indicates the formation of bromide-pure domains *via* light-induced halide migration. To quantify the segregation kinetics, characteristic times were extracted from the PL evolution (Fig. S15). The half-time (*t*_1/2_) denotes the time required for the PL peak to reach the wavelength-midpoint between the initial chloride-rich emission and the final bromide-pure emission, capturing the early-stage halide segregation kinetics. The *t*_90_ and *t*_99_ values describe the time required to reach 90% and 99% of the total redshift, respectively, thereby marking the transition from predominant to virtually bromide-pure emission. For the 20% Cl film, *t*_1/2_ = 160 s, *t*_90_ = 1660 s, and *t*_99_ = 3219 s, indicating relatively slow kinetics across all stages and a gradual approach toward the bromide-pure emission. Increasing the chloride content accelerates initial migration markedly, with *t*_1/2_ decreasing from 160 s (20% Cl) to 107 s (40% Cl) and 67 s (60% Cl), indicating a stronger chemical potential driving force for halide redistribution. The observed trend appears similar to mixed I/Br perovskites, where higher bromide content, stronger illumination, and elevated temperatures have been found to accelerate halide segregation.^14,26,49^ Analogous to the behavior of *t*_1/2_, *t*_90_ = 517 s and *t*_99_ = 2413 s are shorter for 40% Cl, than for 20% Cl (*t*_90_ = 1660 s and *t*_99_ = 3219 s). Surprisingly, for 60% Cl (*t*_90_ = 663 s and *t*_99_ = 3404 s) the halide segregation kinetics slow down compared to 40% Cl and exhibit a pronounced long kinetic tail that does not scale with the faster early-stage dynamics. Considering that the activation energy for iodide migration in mixed I/Br perovskites increases with bromide content as a consequence of a reduced lattice constant,^50,51^ a similar effect could explain why at ultimate stages – where the driving force is less – bromide ion migration becomes sluggish in the chloride-rich films.

UV-vis absorbance measurements performed before and immediately after illumination (Fig. S16) revealed no detectable new peaks or shallow absorption onsets, indicating that the amount of segregated bromide-rich phase is minimal and below the detection limit. Although the amount of the segregated phase is too small to be detected by UV-vis absorbance, it can dominate the PL response *via* efficient charge funneling, resulting in a pronounced redshifted emission.^52^ After 48 h of storage in the dark, PL spectra were recorded again (Fig. S17). For all samples, the fully segregated bromide-rich emission at 539 nm largely disappears, and the halides remix, resulting in a PL peak that closely overlaps with the pristine emission prior to illumination. The 40% Cl film exhibits a weak shoulder at longer wavelengths (around 530 nm). The 60% Cl sample already displays a double peak in the pristine state, in agreement with the asymmetric PL peak observed in the early TRPL (Fig. 3a). After 1 h illumination and 48 h in dark the 60% Cl sample shows a small (∼4 nm) redshift of the chloride-rich peak (to around 470 nm) and a slight increase in the intensity of the bromide-rich peak at 525 nm. Apparently, higher chloride content slows or limits full remixing of the halides, resulting in a less complete recovery of the original emission spectrum, in accordance with the greater intrinsic stability of chloride-containing perovskites.^26^ The difference in spectral shape of steady-state and early-time PL spectra (Fig. 3a) likely arises from the different excitation conditions. TRPL was performed using excitation with a low time-averaged intensity to suppress halide segregation, but the PL spectra shown in Fig. S17 were measured under 1-sun equivalent excitation density.

### Device optimization and stability

Mixed Br/Cl perovskite solar cells with a p–i–n device architecture were fabricated using [2-(9*H*-carbazol-9-yl)ethyl]phosphonic acid (2PACz) as the hole-transport layer (HTL) and [6,6]-phenyl-C_61_-butyric acid methyl ester (PCBM) as the electron-transport layer (ETL). ITO served as the front contact, while LiF/Al was employed as the back contact. The LiF interlayer was used to tune the effective work function of the Al electrode and facilitate efficient electron extraction. The resulting active areas are 0.09 and 0.16 cm^2^. We started the device optimization using the 20% Cl perovskite (FAPbBr_2.4_Cl_0.6_) in which the early-stage halide segregation progresses more slowly than for 40 and 60% Cl. Starting from this configuration, the following section describes the sequential optimization of the device architecture. This involved partial cesium incorporation in the bulk, followed by top-surface passivation and electron-transport layer optimization using a fullerene blend. Together, these three modifications define the optimized device. This optimized protocol was then applied to devices with higher chloride fractions of 40% and 60%.

In mixed-halide I/Br perovskites, partial FA-to-Cs substitution has been shown to enhance structural stability and suppress light-induced halide segregation.^17,18,53^ The smaller Cs cation alleviates the lattice strain caused by the large FA and small Br ions, thereby reducing the Goldschmidt tolerance factor.^54^ Consequently, the resulting less-strained lattice diminishes the formation of iodide vacancies needed to accommodate bromide, limiting halide migration.^19^ To explore whether this approach is also effective for mixed Br/Cl perovskites, where the size mismatch is even larger due to the smaller Cl ions, FA was partially replaced with Cs at concentrations of 0, 2, 4, and 6%. Higher concentrations led to some undissolved material in solution, likely due to the limited solubility of CsBr and possible formation of CsCl through halide exchange with PbBr_2_ and PbCl_2_.

UV-vis absorbance (Fig. S18) for the Cs_*x*_FA_1−*x*_PbBr_2.4_Cl_0.6_ series revealed a sharp absorbance edge across all compositions, corresponding to a fixed optical bandgap of 2.38 eV as determined by Tauc analysis (Fig. S19). This indicates that the introduction of small amounts of cesium has negligible effect on the bandgap. In fact, A-site cation substitution only partially influences the bandgap, because the Pb–halide framework dominates the electronic structure, and A-site cations mainly adjust lattice geometry.^30,31^ As a result, higher Cs concentrations may be required to induce noticeable changes. SEM analysis (Fig. S20 and S21) shows that, compared to the pristine 0% Cs film, the 2% Cs sample exhibits larger grains (from 502 nm to 684 nm), albeit distinct size distributions are present. At higher Cs concentrations (4% and 6%), small crystallites appear at the surface, possibly arising from undissolved Cs-containing precursors. All films preserve a cubic structure with unchanged peak positions in XRD (Fig. S22), indicating once again that low Cs incorporation does not substantially affect the bandgap or lattice parameters. The intensity of the (100) and (200) reflections slightly increase for the 2% and 4% Cs films, suggesting improved crystallinity or preferred orientation at moderate Cs content. All diffractograms show an additional small peak corresponding to the *δ*-phase of FAPbBr_3_ at 12.28°, which is most pronounced at 6% Cs.^55^

In photovoltaic devices (Fig. S23), 2% Cs delivers the highest combination *V*_OC_, *J*_SC_, and FF, resulting in the best overall performance. While the reverse-scan (RS) efficiency is comparable to the pristine 0% Cs device, hysteresis between the RS and forward-scan (FS) is strongly reduced, primarily due to the low FF in the FS of the neat perovskite. Hysteresis is typically linked to ion migration and interfacial charge accumulation.^56^ Even small amounts of Cs can locally relieve lattice strain, increasing the energetic barrier for halide vacancy migration.^57^ Consequently, interfacial polarization effects are mitigated, reducing scan-direction dependence.^58^ At higher Cs contents (4% and 6%), all photovoltaic parameters deteriorate, coinciding with the presence of surface crystals (SEM) and the emergence of the *δ*-phase at 6% Cs (XRD), indicating reduced phase purity.

**Fig. 4:**
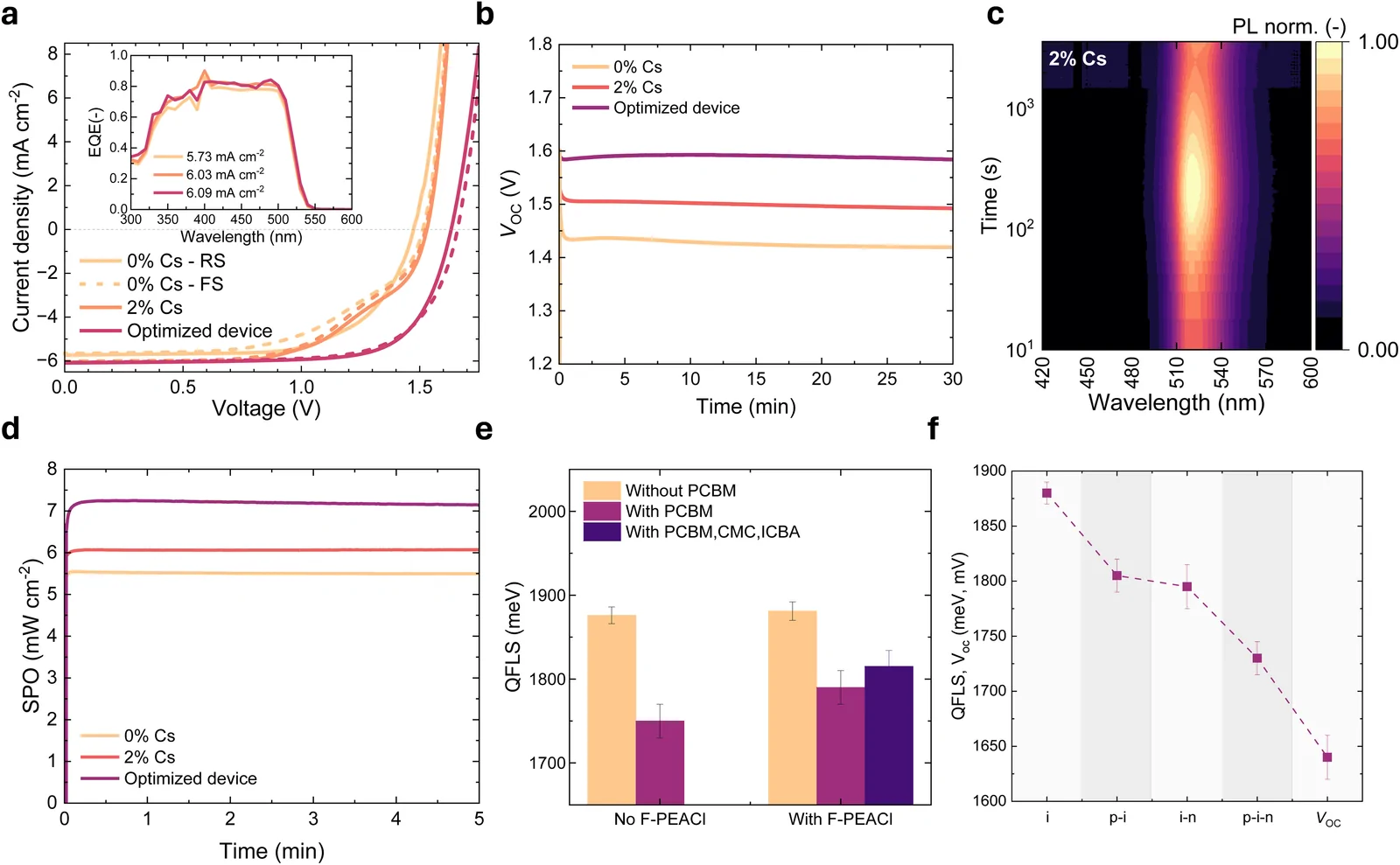
(a) Current density–voltage (*J*–*V*) characteristics in reverse and forward scan directions, and (b) *V*_OC_ tracking of the best performing Cs_*x*_FA_1−*x*_PbBr_2.4_Cl_0.6_ perovskite solar cells processed without (*x* = 0) and with 2% Cs (*x* = 0.02), and of the optimized device (*x* = 0.02) using 1.0 mg mL^−1^ F-PEACl top surface passivation and a 1 : 1 : 1 (w/w/w) mixture of PCBM, CMC, and ICBA as the ETL. The inset in (4a) shows the integrated short-circuit current densities derived from EQE spectra measured under 530-nm light bias at 1-sun equivalent intensity. (c) Normalized PL emission *versus* time of Cs_0.02_FA_0.98_PbBr_2.4_Cl_0.6_ perovskite films during continuous 365 nm illumination at 1-sun equivalent intensity. (d) Stabilized power output of the best performing Cs_*x*_FA_1−*x*_PbBr_2.4_Cl_0.6_ perovskite solar cells processed without (*x* = 0) and with 2% Cs (*x* = 0.02) incorporation in the bulk, and of the optimized device processed with F-PEACl top surface passivation and with a 1 : 1 : 1 (w/w/w) mixture of PCBM, CMC, and ICBA as ETL. (e) QFLS of Cs_0.02_FA_0.98_PbBr_2.4_Cl_0.6_ perovskite films processed without or with F-PEACl top surface passivation and without or with PCBM or a 1 : 1 : 1 (w/w/w) mixture of PCBM, CMC, and ICBA as ETL. (f) QFLS of partial (i, p–i, and i–n) and complete (p–i–n) device stacks compared to the *V*_OC_ of full devices; i represents the active layer (Cs_0.02_FA_0.98_PbBr_2.4_Cl_0.6_) passivated with F-PEACl on glass/HDPA, p–i the active layer on top of glass/ITO/2PACz, i–n the active layer covered with the ETL (PCBM, CMC, ICBA) on glass/HDPA, and p–i–n the complete stack on glass/ITO.

To ensure accurate performance evaluation under 1-sun conditions, the best-performing devices were further analyzed *via* photocurrent spectroscopy to determine the external quantum efficiency (EQE) (Fig. 4a), from which AM1.5G-integrated *J*_SC_ values were extracted and used to calibrate the light intensity in current density–voltage (*J*–*V*) scans (Table 2). Upon 2% Cs incorporation, the EQE-integrated *J*_SC_ increases slightly from 5.73 to 6.03 mA cm^−2^. After calibration, the pristine device exhibits a PCE of 5.5% (RS) and 4.8% (FS), corresponding to a hysteresis index (HI) of 13%, mainly due to a reduced FF of 0.56 in the FS. With 2% Cs, the RS shows improved *V*_OC_ and *J*_SC_, but a reduced FF due to an S-shaped *J*–*V* curve, resulting in a similar PCE of 5.5%. However, hysteresis is strongly suppressed (HI = 3%). The enhanced *V*_OC_ is linked to improved operational stability under illumination: unlike the pristine device, which undergoes a rapid initial *V*_OC_ loss under illumination, the 2% Cs device maintains a much more stable photovoltage, retaining 98% of its initial *V*_OC_ after 30 minutes of continuous illumination compared to 89% for the pristine device (Fig. 4b and S24).

**Table 2:** Photovoltaic parameters from recorded reverse and forward current density–voltage scans of the best performing Cs_*x*_FA_1−*x*_PbBr_2.4_Cl_0.6_ perovskite solar cells processed without (*x* = 0) and with 2% Cs (*x* = 0.02) incorporation in the bulk, and of the optimized device processed with F-PEACl top surface passivation and with a 1 : 1 : 1 (w/w/w) mixture of PCBM, CMC, and ICBA as ETL

Sample	Scan	*V*_OC_ [V]	FF [—]	*J*_SC_ [mA cm^−2^]^a^	PCE [%]	HI [%]
0% Cs	Reverse	1.47	0.65	5.74	5.5	13
	Forward	1.51	0.56	5.66	4.8	
2% Cs	Reverse	1.53	0.60	6.05	5.5	3
	Forward	1.52	0.58	6.02	5.3	
Optimized device	Reverse	1.63	0.73	6.09	7.3	2
	Forward	1.66	0.70	6.07	7.1	

a From integrating the EQE with the AM1.5 G irradiance.

To assess the stabilizing effects of Cs incorporation on the halide dynamics, the PL evolution under continuous illumination was monitored for Cs_0.02_FA_0.98_PbBr_2.4_Cl_0.6_ (Fig. 4c and S25). The introduction of 2% Cs markedly stabilizes the perovskite: the main PL peak at 520 nm shifts by only 1 nm after 20 min and by 2 nm after 30 min, with the appearance of a minor shoulder at higher wavelengths indicating the formation of a slightly bromide-enriched region. After 1 h, the total peak shift is limited to 4 nm. This is in stark contrast with the Cs-free film for which the same shift occurs in about 30 s.

Top surface passivation with 4-fluorophenethylammonium chloride (F-PEACl) was employed to further improve device performance. XPS confirms the presence of fluorine after F-PEACl treatment (Fig. S26), while SEM shows the formation of a surface layer (Fig. S27), and XRD indicates a 2D Ruddlesden–Popper phase at higher concentrations (Fig. S28).^59–62^ Increasing the F-PEACl concentration up to 1.0 mg mL^−1^ improves the *V*_OC_ and FF, whereas higher concentrations reduce *J*_SC_ due to insulating overlayer formation and increase hysteresis (Fig. S29).^63^ The F-PEACl treatment (Fig. S30 and Table S7) was combined with a ternary ETL blend of PCBM, CMC, and ICBA in a 1 : 1 : 1 (w/w/w) ratio, as previously reported,^9^ defining the optimized device (Fig. 4a and Table 2). This configuration suppresses interfacial recombination and improves charge extraction.^64,65^ Consequently, a PCE of 7.3% (RS) is achieved with enhanced *V*_OC_ (1.63–1.66 V) and FF while maintaining the *J*_SC_, alongside a minimal hysteresis (HI = 2%). In addition, the optimized device exhibits excellent photovoltage stability under continuous illumination, maintaining virtually 100% of its initial *V*_OC_ after 30 minutes (Fig. 4b and S24). To further evaluate device performance under continuous 1-sun illumination, stabilized power output (SPO) measurements were performed on the best-performing Cs-free, 2% Cs, and optimized devices (Fig. 4d and S31). All three devices exhibit nearly constant normalized power output over 5 minutes, with less than 1% variation, demonstrating stable operation under continuous illumination.

APL measurements were performed to quantify nonradiative losses in Cs-containing and F-PEACl-treated films, both in the absence and presence of different ETLs. For perovskite films (Fig. S32), the QFLS remains unchanged upon introducing 2% Cs or F-PEACl treatment (1883 ± 3 meV), corresponding to a nonradiative loss of 167 ± 3 meV relative to the SQ limit (Table S5). This indicates that neither modification significantly affects charge recombination in the films. Instead, Cs primarily enhances operational stability, while F-PEACl influences interfacial charge recombination when an ETL is present (Fig. 4e), consistent with the substantial enhanced FF seen for the optimized device. Deposition of PCBM on a non-passivated perovskite reduces the QFLS by 126 meV, whereas insertion of F-PEACl lowers this loss to 91 meV, confirming reduced interfacial recombination. Replacing PCBM with the ternary fullerene blend further decreases the loss to 66 meV. This progressive improvement in QFLS directly correlates with the observed increase in *V*_OC_.

Interfacial voltage losses were further analyzed by extracting the QFLS of individual sub-stacks (i, p–i, i–n) and the full p–i–n stack (Fig. 4f). The intrinsic absorber exhibits a QFLS of 1880 meV on glass/HDPA (i), which decreases by 75 meV when deposited on ITO/2PACz (p–i), and by 85 meV when PCBM is deposited on top (i–n). The similar reduction indicates comparable recombination losses induced by both HTL and ETL. This contrasts with narrower-bandgap perovskites, where ETL losses typically dominate, and suggests that neither charge transport layer (CTL) is ideally matched to the wide-bandgap absorber. When combined in the full p–i–n stack, the QFLS decreases by 150 meV relative to the i stack to 1730 meV, still exceeding the measured *V*_OC_ of 1640 mV by 90 meV, pointing to additional voltage losses from interfacial barriers and energetic misalignment in the operating device.

**Fig. 5:**
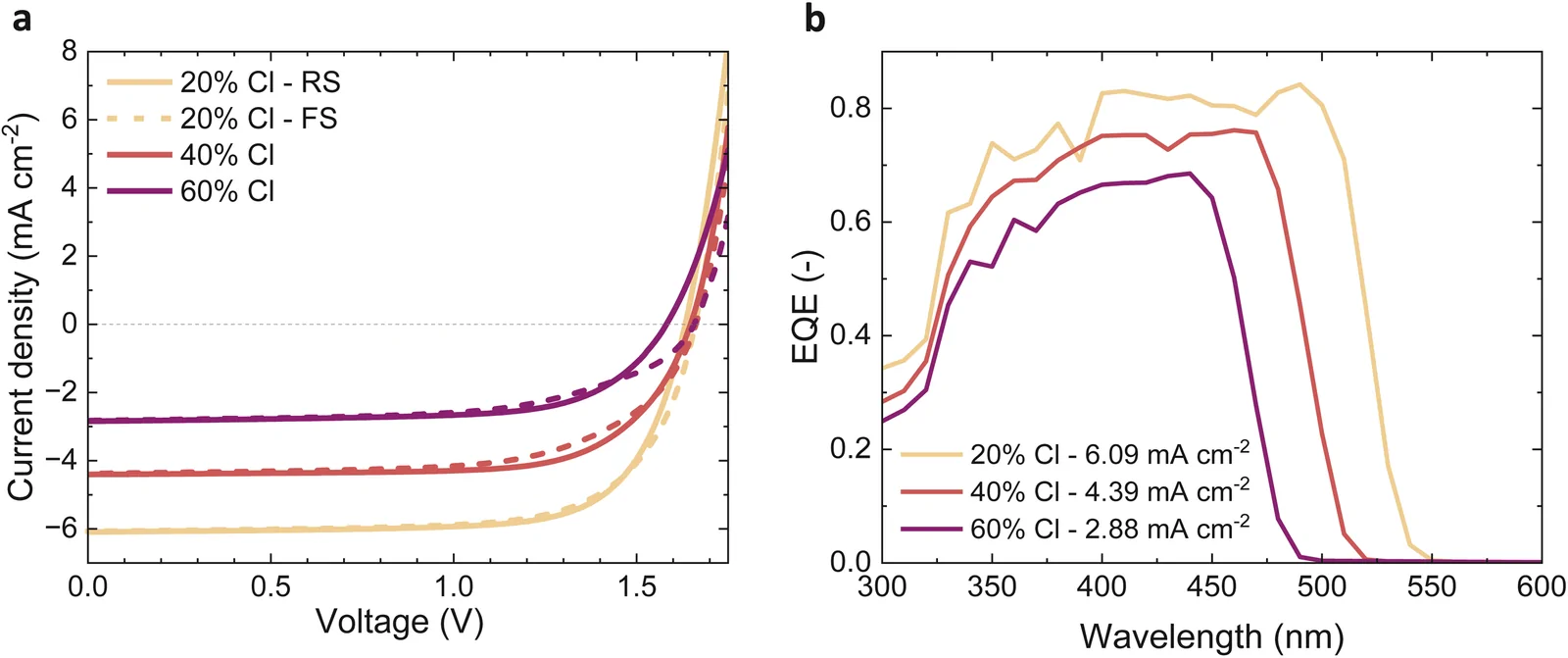
(a) Current density–voltage (*J*–*V*) characteristics in reverse and forward scan directions of the best performing Cs_0.02_FA_0.98_PbBr_3−*x*_Cl_*x*_ (*x* = 0.6, 1.2, and 1.8) perovskite solar cells with 1.0 mg mL^−1^ F-PEACl top surface passivation and a 1 : 1 : 1 (w/w/w) mixture of PCBM, CMC, and ICBA as ETL. (b) Corresponding integrated short-circuit current densities derived from EQE spectra measured under 530-nm light bias at 1-sun equivalent intensity.

The optimized device and passivation strategy found for 20% Cl, *i.e.*, incorporating 2% Cs, surface passivation with 1.0 mg mL^−1^ F-PEACl, and the ternary ETL blend, was then used to fabricate Cs_0.02_FA_0.98_PbBr_3−*x*_Cl_*x*_ solar cells with *x* = 1.2 and 1.8 (40% and 60% Cl). The devices exhibit PCEs of 5.1% and 3.0% in RS, respectively (Fig. 5a and Table 3). The *J*_SC_ decreases progressively with increasing chloride content, consistent with the reduced absorption at wider bandgaps. The maximum EQE (Fig. 5b) also decreases from ∼80% to ∼75% and ∼68%, which may – at least in part – be related to reduced film thickness upon progressive substitution of Br with Cl (Table S6). The bandgap values derived from the EQE spectra (Fig. S33) are in excellent agreement with those obtained from Tauc analysis (Table 1). In FS, the *V*_OC_ saturates at 1.66 V for all three compositions and does not follow the increasing QFLS of the neat perovskite films with increasing chloride content (Fig. 3c). This suggests an energetic misalignment at the CTLs. The HI remains moderate at 7 and 8%, mainly due to a slightly lower FF in the FS. These results demonstrate that reasonably efficient devices can still be realized at high chloride fractions; however, further improvements require the development of CTLs and contacts better suited for such wide-bandgap absorbers, potentially drawing inspiration from materials and contact strategies employed in wide-bandgap perovskite light-emitting diodes. Overall, the device performances achieved, together with the pronounced increase in visible-light diffused transmission at higher chloride contents (Fig. S34), reinforce the suitability of mixed Br/Cl perovskites as promising absorbers for semi-transparent photovoltaic and BIPV applications, where a balance between transparency and photovoltaic efficiency is required.

**Table 3:** Photovoltaic parameters from recorded reverse and forward current density–voltage scans of the best performing Cs_0.02_FA_0.98_PbBr_3−*x*_Cl_*x*_ (*x* = 0.6, 1.2, and 1.8) perovskite solar cells with 1.0 mg mL^−1^ F-PEACl top surface passivation and a 1 : 1 : 1 (w/w/w) mixture of PCBM, CMC, and ICBA as ETL

Sample	Scan	*V*_OC_ [V]	FF [—]	*J*_SC_ [mA cm^−2^]^a^	PCE [%]	HI [%]	PCE/PCE^SQ^ [%]
20% Cl	Reverse	1.63	0.73	6.09	7.3	2	49
	Forward	1.66	0.70	6.07	7.1		
40% Cl	Reverse	1.65	0.71	4.40	5.1	8	42
	Forward	1.66	0.65	4.38	4.7		
60% Cl	Reverse	1.58	0.68	2.84	3.0	7	33
	Forward	1.66	0.60	2.82	2.8		

a From integrating the EQE with the AM1.5 G irradiance.

## Conclusion

In summary, the structural and optoelectronic properties of FAPbBr_3−*x*_Cl_*x*_ perovskites are tightly governed by the chloride fraction. While chloride incorporation induces near-linear bandgap widening and lattice contraction, it also significantly slows crystallization dynamics. In particular, reduced nucleation kinetics promote the formation of distinct morphologies with enlarged grain size. All mixed bromide/chloride perovskites undergo light-induced halide segregation, with faster early-stage segregation at higher chloride fractions, though domains remix in the dark. Small amounts of cesium, replacing formamidinium at the A-site, were very effective in stabilizing the perovskite under continuous illumination. Top surface passivation improved device performance considerably. Increasing the chloride content beyond 20% did not yield a higher *V*_OC_, despite a higher QFLS, emphasizing that interface-related losses remain a key limitation in wide-bandgap perovskite absorbers.

## Author contributions

L. B. conceptualized and designed the study, prepared and optimized the films and devices, measured UV-vis absorbance and diffuse transmission spectra, XRD, XPS, depth-profiling, thickness and roughness, *J*–*V*, EQE, *V*_OC_ and SPO tracking, analyzed and interpreted the data, and prepared the initial draft of the manuscript. G. J. W. A. performed TRPL measurements and simulations, and calculated the exciton binding energies and Shockley–Queisser limits. S. V. Q. M. assisted with halide segregation measurements. N. R. M. S. assisted with *in situ* UV-vis absorption measurements. W. H. M. R. carried out APL measurements. L. M. K. performed SEM measurements. C. H. L. W. carried out UPS measurements. M. M. W. provided supervision. R. A. J. J. provided supervision and secured funding. All authors reviewed and approved the final draft of the manuscript.

## Conflicts of interest

The authors declare no competing financial interest.

## Supplementary Material

EL-002-D6EL00097E-s001

EL-002-D6EL00097E-s002

## Data Availability

All relevant data supporting the findings of this study are included within the supplementaryinformation (SI), including detailed methods, additional structural and optical characterization data, and supplementary device performance and stability measurements. Ref. 34 and 66 are cited in the SI. Additional data are available from the corresponding author upon reasonable request. Supplementary information is available. See DOI: https://doi.org/10.1039/d6el00097e.
