# Supplementary material for: Influence of chloride on the crystallization and photostability of mixed-halide FAPbBr3−xClx perovskites for photovoltaics
Source: EES Solar. 2026 Aug 4;2(5):1250–62. doi: 10.1039/d6el00097e (PMC13456141; doi:10.1039/d6el00097e)
Supplement: Supplementary file 1 [file EL-002-D6EL00097E-s002.pdf]

Supplementary Information (SI) for EES Solar.
This journal is © The Royal Society of Chemistry 2026

Supplementary Information for

Influence of Chloride on the Crystallization and Photostability of Mixed-Halide $FAPbBr_{3-x}Cl_x$ Perovskites for Photovoltaics

Laura Bellini,[a] Guus J. W. Aalbers,[a] Simon V. Quiroz Monnens,[a] Nick R. M. Schipper,[a] Willemijn H. M. Remmerswaal,[a] Lana M. Kessels,[a] Christ H. L. Weijtens,[a] Martijn M. Wienk[a] and René A. J. Janssen[ab]*

[a] Materials to Optoelectronic Devices and Institute of Complex Molecular Systems
Eindhoven University of Technology
P.O. Box 513, 5600 MB Eindhoven, The Netherlands
E-mail: r.a.j.janssen@tue.nl

[b] Dutch Institute for Fundamental Energy Research
De Zaale 20, 5612 AJ Eindhoven, The Netherlands

Experimental Section

Materials

All chemicals were handled as received and stored in an N_2-filled glovebox. Patterned indium tin oxide (ITO) coated glass substrates were supplied by Naranjo BV. Formamidinium bromide (FABr, >99.99%), formamidinium chloride (FACl, >99.99%), and 4-fluorophenethylammonium chloride (F-PEACl, >99.99%) were obtained from Greatcell Solar Materials. Cesium bromide beads (CsBr, 99.999% trace metal basis), isopropanol (IPA, anhydrous, >99.5%), ethanol (EtOH, anhydrous, >99.5%), chlorobenzene (CB, anhydrous, 99.8%), and chloroform (CF, anhydrous, 99.0%) were purchased from Sigma Aldrich. Lead(II) bromide ($PbBr_2$, >99.0%), lead(II) chloride ($PbCl_2$, >99.0%), [2-(9*H*-Carbazol-9-yl)ethyl]phosphonic acid (2PACz, >98.0%), and C_{60}-fused *N*-methylpyrrolidine-*m*-C_{12}-phenyl (CMC, >97.0%) were sourced from TCI. [6,6]-Phenyl-C_{61}-butyric acid methyl ester (PCBM, 99.0%) was supplied by Solenne BV. 1′,1″,4′,4″-Tetrahydro-di[1,4]methanonaphthaleno[1,2:2′,3′,56,60:2″,3″][5,6]fullerene-C_{60} (ICBA) was obtained from Lumtec. *N,N*-Dimethylformamide (DMF, anhydrous, 99.8%), and dimethyl sulfoxide (DMSO, anhydrous, >99.9%) were purchased from Thermo Fischer.

Precursor solutions preparation

Mixed-halide $FAPbBr_{3-x}Cl_x$ precursor solutions with nominal compositions of x = 0.0, 0.6, 1.2, 1.8, and 2.4 were formulated following a two-step approach. In this procedure, the inorganic lead halide precursors PbX_2 (X= Br or Cl) were dissolved at a total concentration of 1.25 M in a DMF:DMSO 4:1 solvent mixture (v/v), while the corresponding organic halide salts FAX (X= Br or Cl) were separately dissolved in IPA at a concentration of 0.49 M. Both precursor solutions were magnetically stirred at 60 °C overnight to ensure complete dissolution and subsequently cooled to ambient temperature prior to deposition. Due to its poor solubility in IPA, CsBr (0, 2, 4, and 6 mol% relative to the total FAX content) was added to the PbX_2 precursor solution. To enable complete dissolution, the solvent ratio was adjusted to DMF:DMSO 2:1 (v/v). The FAX content was correspondingly reduced to maintain the overall stoichiometry.

For the hole-transport layer, 2PACz (0.33 mg mL^{-1}) was dissolved in ethanol under vortex mixing. For surface passivation, F-PEACl was dissolved in IPA (1.0 mg mL^{-1}) and stirred overnight at 60 °C. Electron-transport layer solutions were prepared by dissolving PCBM, CMC, or ICBA individually at 20 mg mL^{-1} in a CB:CF 1:1 (v/v) solvent mixture. The ternary mixture was obtained by mixing the corresponding stock solutions in a 1:1:1 (w/w/w) ratio, stirred overnight at 60 °C and filtered using 0.22 μm PTFE syringe filters prior to use.

Film fabrication

Glass substrates were cleaned by ultrasonication in IPA for 15 min and treated with UV-ozone for 30 min immediately before spin-coating. Perovskite films were deposited via a sequential spin-coating process. The PbX_2 precursor solution (120 µL) was first dispensed statically onto the substrate and spin-coated for 30 s at 3000 rpm, followed by an additional 30 s spin step at 3300 rpm. Thirty seconds after the onset of spinning, the FAX solution (100 µL) dynamically applied onto the rotating substrate. All films were then annealed for 30 min at 100 °C.

Device fabrication

Patterned indium tin oxide (ITO) coated glass substrates were cleaned with ultrasonication in acetone for 15 min, scrubbed with a soap-water solution, ultrasonicated for 15 min in soap-water, rinsed with deionized water, and ultrasonicated for 15 min in isopropanol. Next, the substrates were treated with UV-ozone for 30 min immediately before spin-coating. The HTL (120 µL) was deposited by spin-coating at 3000 rpm for 30 s and annealed at 100 °C for 5 min. After cooling, the perovskite layer was deposited as described above. Surface passivation was applied by dynamically spin-coating the F-PEACl solution (100 µL) at 4000 rpm for 30 s, followed by annealing at 100 °C for 5 min. The fullerene-based electron-transport layer (100 µL) was then spin-coated at 1000 rpm for 30 s and annealed at 100 °C for 10 min. A 1 nm LiF layer and a 100 nm Al electrode were subsequently deposited by thermal evaporation under high vacuum. The device active area, defined by the overlap of the ITO and top electrode, was 0.09 cm^2 or 0.16 cm^2.

Film characterization

A PerkinElmer Lambda 1050 spectrometer equipped with a 60 mm integrating sphere was used to measure ultraviolet-visible (UV-vis) absorbance and diffuse transmission spectra of the perovskite films under ambient conditions. Exciton binding energies (E_{B}) were determined by fitting the UV-vis absorbance spectra using the Elliott band-fluctuations (EBF) model following Lizárraga et al.[34] with six free parameters according to:

$$\alpha(E) = \frac{\sqrt{E_{\mathrm{B}}}}{E}\left[2A_1E_{\mathrm{B}}\sum_{n=1}^{N}\frac{1}{n^3}\frac{\exp\left(\frac{E_{x,n}-E}{\sigma_n}\right)}{\sigma_n\left(1+\exp\left(\frac{E_{x,n}-E}{\sigma_n}\right)\right)^2} + A_2\left(\frac{1}{1+\exp\left(\frac{E_{\mathrm{g}}-E}{\sigma_{\mathrm{c}}}\right)} + \frac{1}{\exp\left(2\pi\sqrt{\frac{E_{\mathrm{B}}}{E-E_{\mathrm{g}}}}\right)-1}\right)\right] \quad (1)$$

With the excitonic peak position as $E_{x,n} = E_{\mathrm{g}} - E_{\mathrm{B}}/n^2$ and the Toyozawa broadening as $\sigma_n = \sigma_{\mathrm{c}} - (\sigma_{\mathrm{c}} - \sigma_{\mathrm{d}})/n^2$. Here, E is the photon energy, E_{g} is the bandgap, $\sqrt{E_{\mathrm{B}}}/E$ constitutes the physical prefactor of the model, E_{B} is the exciton binding energy, σ_{d} and σ_{c} are the excitonic and continuum broadening, A_1 and A_2 are excitonic and continuum transition amplitudes, respectively. N is the number of exciton levels which we fix at 3 for room temperature. The fitting window is $E_{\mathrm{g}} - 0.15 < E < E_{\mathrm{g}} +$

0.20 eV. The first part is the excitonic term in which the total number of excitonic levels is summed from $n = 1$ to N of including a level-dependent broadening following the Toyozawa relation. The second part is the continuum term that includes a Fermi-function for the broadband edge and a Sommerfeld enhancement factor that corrects for the residual Coulomb attraction between free carriers above the bandgap. Since the UV-vis absorbance was normalized to its maximum (within the fitting energy range), the amplitudes for A_1 and A_2 absorb all dimensional prefactors and carry no absolute physical meaning, only the ratio between A_2/A_1. The parameters E_g, E_B, σ_d, and σ_c are determined exclusively by the UV-vis absorbance line shape and are unaffected by the normalization. Fitting was performed by nonlinear least squares with a coarse grid initialization over (E_g, E_B) followed by local refinement with random restarts.

X-ray diffraction (XRD) was recorded using a Bruker 2D phaser (Cu Kα radiation). Measurements were performed in the range of 10–40° with a step size of 0.01° and collection time of 0.2 s for the different chloride ratios, and with a step size of 0.05° and collection time of 0.5 s for Cs-containing samples. For F-PEACl-passivated samples, measurements were performed in the range of 3–40° to detect the formation of any 2D perovskite phase. A step size of 0.02° and a collection time of 0.2 s were used.

X-ray photoemission spectroscopy (XPS) measurements were carried out using a Thermo Scientific K-Alpha spectrometer equipped with a 180° double-focusing hemispherical analyzer and a 128-channel detector. Spectra were collected using monochromatic Al Kα radiation with an X-ray spot size of 400 µm. High-resolution spectra of individual core levels were recorded using a pass energy of 50 eV, with 30 accumulated scans and a dwell time of 50 ms. Depth profiling was performed in etching mode using an ion energy of 500 eV and a low ion current, corresponding to an estimated sputter rate of approximately 0.05 nm s^{-1} on Ta_2O_5. Each sputtering cycle had a duration of 30 s.

Ultraviolet photoelectron spectroscopy (UPS) measurements were carried out using a VG EscaLab II instrument equipped with a He I excitation source (21.22 eV). A –6 V sample bias was applied during acquisition. The base pressure in the analysis chamber was approximately 1×10^{-10} mbar, increasing to 1×10^{-6} mbar during the experiment.

Film thicknesses were measured using a Veeco Dektak 150 profilometer.

Scanning electron microscopy images were acquired with an FEI Quanta 3D FEG microscope operated at 5 keV after sputtering a thin gold layer onto the samples.

In-situ UV-vis absorption measurements were obtained by converting time-resolved reflectance spectra into absorption. To suppress transmission contributions, the backside of the glass substrates was coated with white spray paint. This opaque, highly scattering layer effectively prevents light from being transmitted through the sample, rendering the transmittance term negligible and

allowing absorptance to be approximated as one minus the measured reflectance. Measurements were performed under nitrogen with samples placed on either a spin coater or hotplate. A halogen light source was focused onto the sample, and scattered light was collected off-specular using an optical fiber coupled to a spectrometer. Absorptance values were calculated as:

$$A(\lambda, t) = -\log_{10}\left(\frac{I_m(\lambda,t) - I_{m,dark}(\lambda)}{I_{m,blank}(\lambda) - I_{m,dark}(\lambda)}\right) \quad (2)$$

Where $I_m(\lambda, t)$ is the absorptance, $I_{m,dark}(\lambda)$ represents the noise, and $I_{m,blank}(\lambda)$ represents a measurement on a white substrate without perovskite (thus, full reflection).

For transient photoluminescence (TRPL), a pulsed Continuum Surelite-II 10 Nd:YAG laser with a 355 nm wavelength output was used as the excitation source with a repetition rate of 10 Hz. The laser beam passed through an aperture and hit the sample normal to the surface with a circular spot diameter of 2 mm. The excitation fluence was around 9.55 μJ cm^{-2}, resulting in an average initial charge carrier density (Δn) of ~3.5×10^{17} cm^{-3}. The carrier density *x*-axis under high-level injection conditions was calculated as

$$n(t) = \Delta n \sqrt{\frac{\phi_{\mathrm{TRPL}}(t)}{\phi_{\mathrm{TRPL}}(0)}} \quad (3)$$

Where $n(t)$ is the time-dependent carrier density, $\phi_{\mathrm{TRPL}}(t)$ is the time-dependent PL intensity, and $\phi_{\mathrm{TRPL}}(0)$ is the PL intensity after the laser pulse. The emitted PL signal was focused on an optical fiber and coupled into a Princeton Instruments SpectraPro 300 spectrometer, and the spectrally dispersed signals were analyzed using a Roper Scientific PI-MAX HQ intensified CCD camera. To yield time-resolved PL spectra, the gate functionality of the iCCD camera was used along with an external trigger from the laser to capture a PL spectrum at changing waiting times after the trigger. The TRPL trace was converted into a differential decay time according to:

$$\tau_{\mathrm{diff}}(t) = \left(-\frac{\mathrm{d\,l}\ \ [\phi_{\mathrm{TRPL}}(t)]}{2\mathrm{d}t}\right)^{-1} \quad (4)$$

Where τ_{diff} is the differential decay time, ϕ_{TRPL} is the TRPL intensity, and t is time.

Absolute photoluminescence (APL) for Quasi-Fermi level splitting (QFLS) was assessed through absolute photoluminescence measurements where excitation was achieved using a 405 nm Thorlabs M405F3 fiber-coupled LED. Samples were placed underneath an Avantes AvaSphere-30-REFL integrating sphere equipped with in-line filter holders for excitation light and emitted light, holding a 425 nm short-pass filter (Edmund Optics) and a 450 nm long-pass filter (Edmund Optics), respectively. To allow a prediction of the open circuit voltage corresponding to AM1.5G illumination conditions, the excitation photon flux in the experiment was adjusted to correspond to the number of photons that would be absorbed for broad-band AM1.5G excitation. The intensity of the LED was adjusted to a 1-sun equivalent intensity by measuring the illumination with a power meter (Ophir

Nova) and matching this to the current density (J_{SC}) of the perovskite solar cells. The integrating sphere was connected to an Avantes AvaSpec-HSC1024X58TEC-EVO spectrometer by an optical fiber. The setup was calibrated using an Avantes halogen lamp yielding a spectral correction factor. Spectral photon fluxes (φ_{PL}) were obtained after a Jacobian transformation. Using the nonlinear least squares fit method in MATLAB, the QFLS was determined from the φ_{PL}. The relation between QFLS and photon flux is defined as follows:

$$\varphi_{PL} = \frac{1}{4\pi^2\hbar^3c^2}\frac{a(E)E^2}{\exp\left(\frac{E-\Delta\mu}{k_BT}\right)-1} \quad (5)$$

Where $a(E)$ is the photon energy-dependent absorptivity, $\hbar$ the reduced Planck constant, c the speed of light in vacuum, k_B the Boltzmann constant, T the temperature, and $\Delta\mu$ the QFLS. The $a(E)$ is assumed to be unity for photon energies sufficiently larger than the optical bandgap.

Continuous photoluminescence (PL) measurements for halide-segregation were performed using an M365L2 LED (Thorlabs, M00517550) as the excitation source. The emitted light was collimated onto the sample using a 2M2P50-A collimator. Condenser lenses (LA1384-AB-ML) collected and focused the resulting PL onto an optical fiber (Avantes FC-UV800-2ME-FC/PC), with a 390/420 nm high-pass filter positioned before the fiber. The light was transmitted through the fiber to an Avantes NIR spectrometer (1201161U2), and spectra were recorded using Avantes Avasoft software. The setup was calibrated to an irradiance equivalent to 1 sun. A dark spectrum was acquired prior to measurement and subtracted from the PL spectrum to correct for background contributions. During continuous PL measurements, approximately 1 cm^2 of the sample was illuminated with the 365 nm LED. UV-vis absorption measurements were subsequently performed on the same region to probe halide segregation.

Device characterization

Current density–voltage (JV) characteristics were measured under simulated AM1.5G illumination generated using a tungsten-halogen lamp (100 mW cm^{-2}) in combination with UV (Schott GG385) and daylight (Hoya LB120) correction filters. Electrical characterization was performed using a Keithley 2400 source-measure unit. JV curves were recorded by sweeping the applied bias from +2.0 V to –0.5 V for reverse scans, or vice versa for forward scans, using a voltage increment of 0.01 V and a scan rate of 0.25 V s^{-1}. The same measurement setup was used to monitor the open-circuit voltage (V_{OC}) over time under continuous AM1.5G illumination. During V_{OC} tracking, the device was maintained under open-circuit conditions while the voltage was recorded as a function of illumination time. Subsequently, a slow JV scan was performed to determine the stabilized maximum power point

voltage (V_{MPP}). Stabilized power output (SPO) measurements were then carried out by holding the device at this fixed V_{MPP} under continuous 1-sun illumination while recording the current over time.

External quantum efficiency (EQE) spectra were measured using a monochromatic illumination setup. Light from a tungsten-halogen lamp (Philips Focusline, 50 W) was mechanically modulated at 165 Hz using an optical chopper (Stanford Research SR540) before being spectrally dispersed by a monochromator (Oriel Cornerstone 130). The monochromatic beam passed through an aperture defining an illuminated area of 0.0314 cm^2 on the device. The generated photocurrent was amplified using a low-noise current preamplifier (Stanford Research SR570) and detected with a lock-in amplifier (Stanford Research SR830). The incident photon flux was calibrated using a silicon reference photodiode. To determine the short-circuit current density under near-1-sun conditions during EQE measurements, a continuous-wave 530 nm LED (Thorlabs M530L3) driven by a Thorlabs DC4104 current controller was used to provide a steady bias illumination. This bias light enabled accurate extraction of the EQE response under operating conditions comparable to solar illumination. Because of insufficient blue-light content of the illumination setup, the initially measured *JV* curves correspond to sub-1 sun illumination conditions. Unless mentioned otherwise, for all cells reported in this study, the lamp intensity was adjusted to match the J_{SC} with the EQE-integrated photocurrent of the same device, measured with bias illumination, to ensure an accurate determination of the PCE. The optical bandgap was determined from the EQE spectra by calculating the first derivative of the EQE response with respect to photon energy. The resulting derivative spectrum was fitted with a Gaussian function, and the center of the fitted peak was taken as the optical bandgap.

Additional Figures and Tables

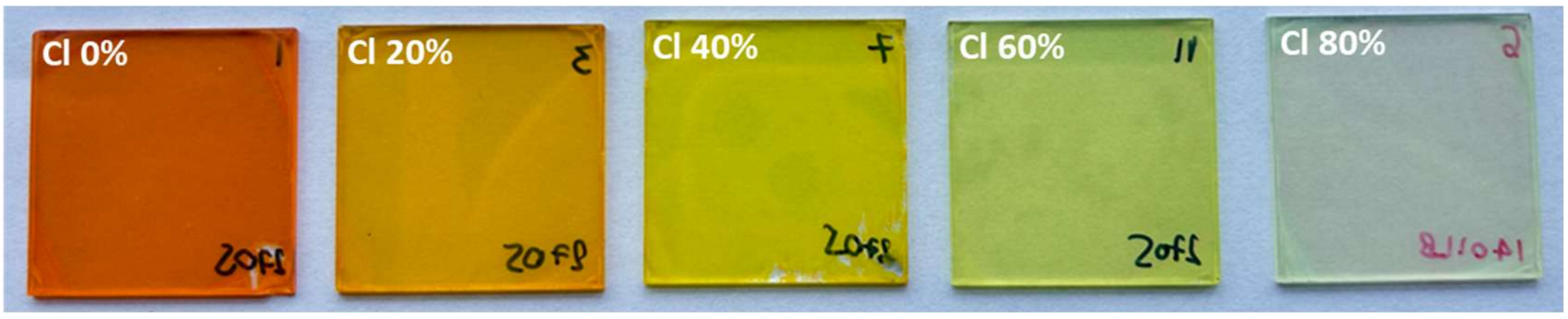

Fig. S1 Photograph of $FAPbBr_{3-x}Cl_x$ perovskite films containing 0, 20, 40, 60, and 80% Cl relative to the total halide concentration.

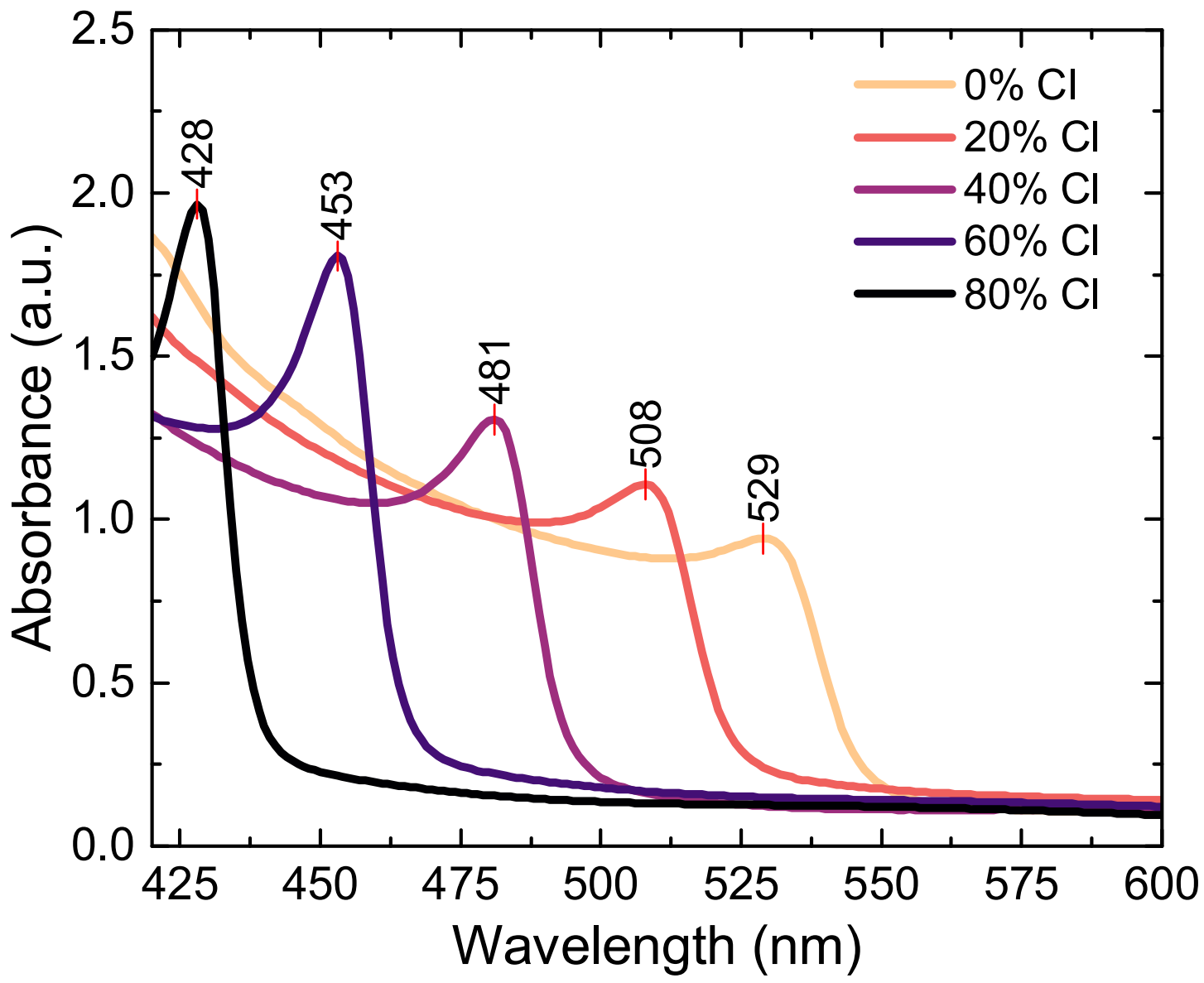

Fig. S2 UV-vis absorbance spectra of $FAPbBr_{3-x}Cl_x$ perovskite films containing 0, 20, 40, 60, and 80% Cl relative to the total halide concentration.

Table S1 Optical bandgaps determined from Tauc analysis of $FAPbBr_{3-x}Cl_x$ perovskite films containing 0, 20, 40, 60, and 80% Cl relative to the total halide concentration.

Cl content [%]	Optical bandgap [eV]
0	2.28
20	2.38
40	2.52
60	2.68
80	2.85

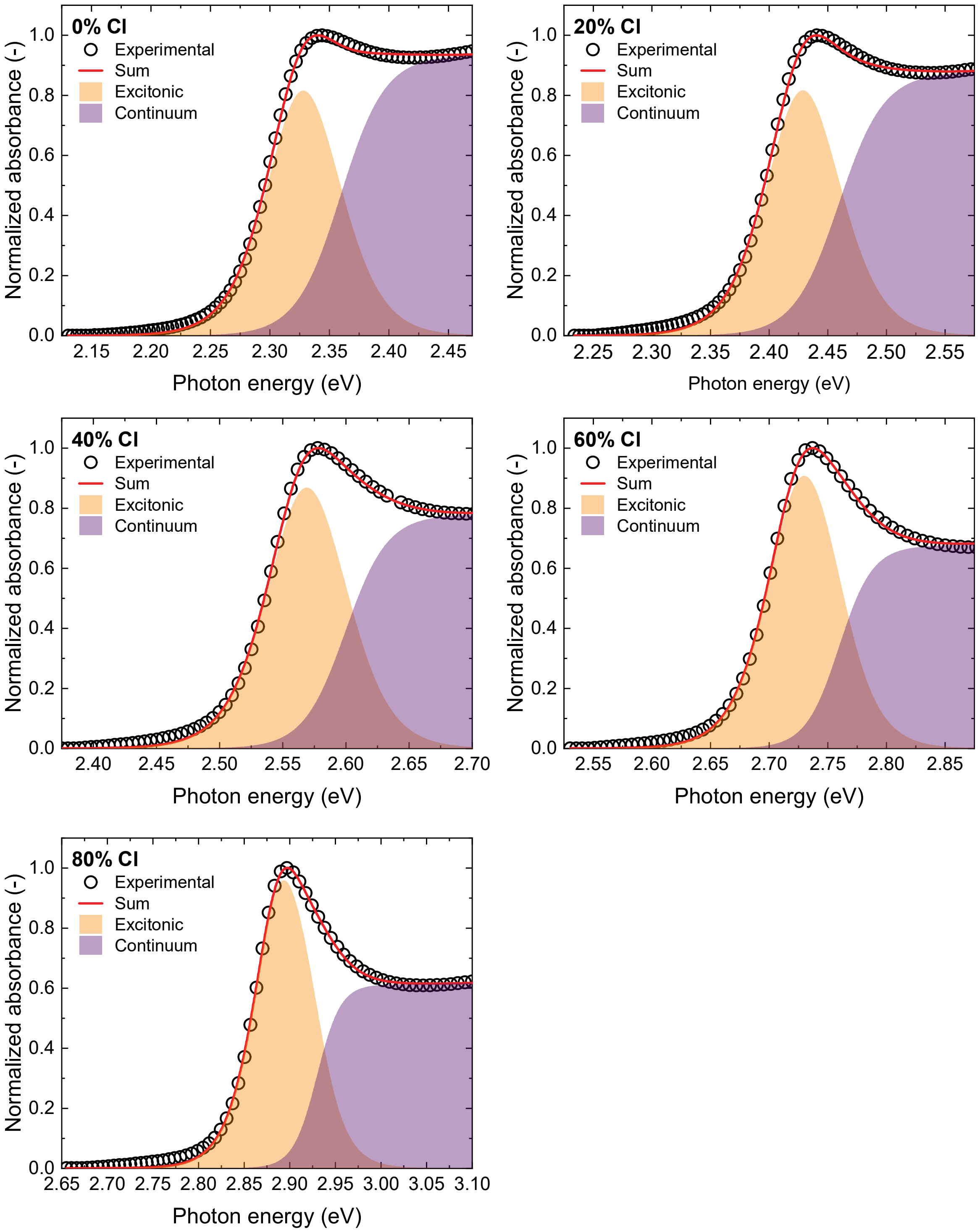

Fig. S3 Deconvolution of UV-vis absorbance spectra into excitonic and continuum absorption contributions using Elliott's equation for $FAPbBr_{3-x}Cl_x$ perovskite films containing with 0, 20, 40, 60, and 80% Cl relative to the to the total halide concentration.

Table S2. Optimized fit parameters of Elliott's equation (1) to the experimental absorbance spectra shown in Fig. S3.

Cl [%]	E_g [eV]	E_B [meV]	σ_d [meV]	σ_c [meV]	A_1 [−]	A_2 [−]	R^2 [−][a]	A_2/A_1 [−]
0	2.36	34.4	20.0	18.7	0.97	1.10	0.9996	1.13
20	2.46	33.6	20.5	19.2	1.05	1.06	0.9994	1.01
30	2.60	34.0	20.1	16.7	1.28	1.11	0.9993	0.87
40	2.76	33.5	19.5	14.3	0.84	0.61	0.9993	0.73
60	2.93	40.2	19.9	13.1	1.33	0.97	0.9991	0.72

[a] Coefficient of determination.

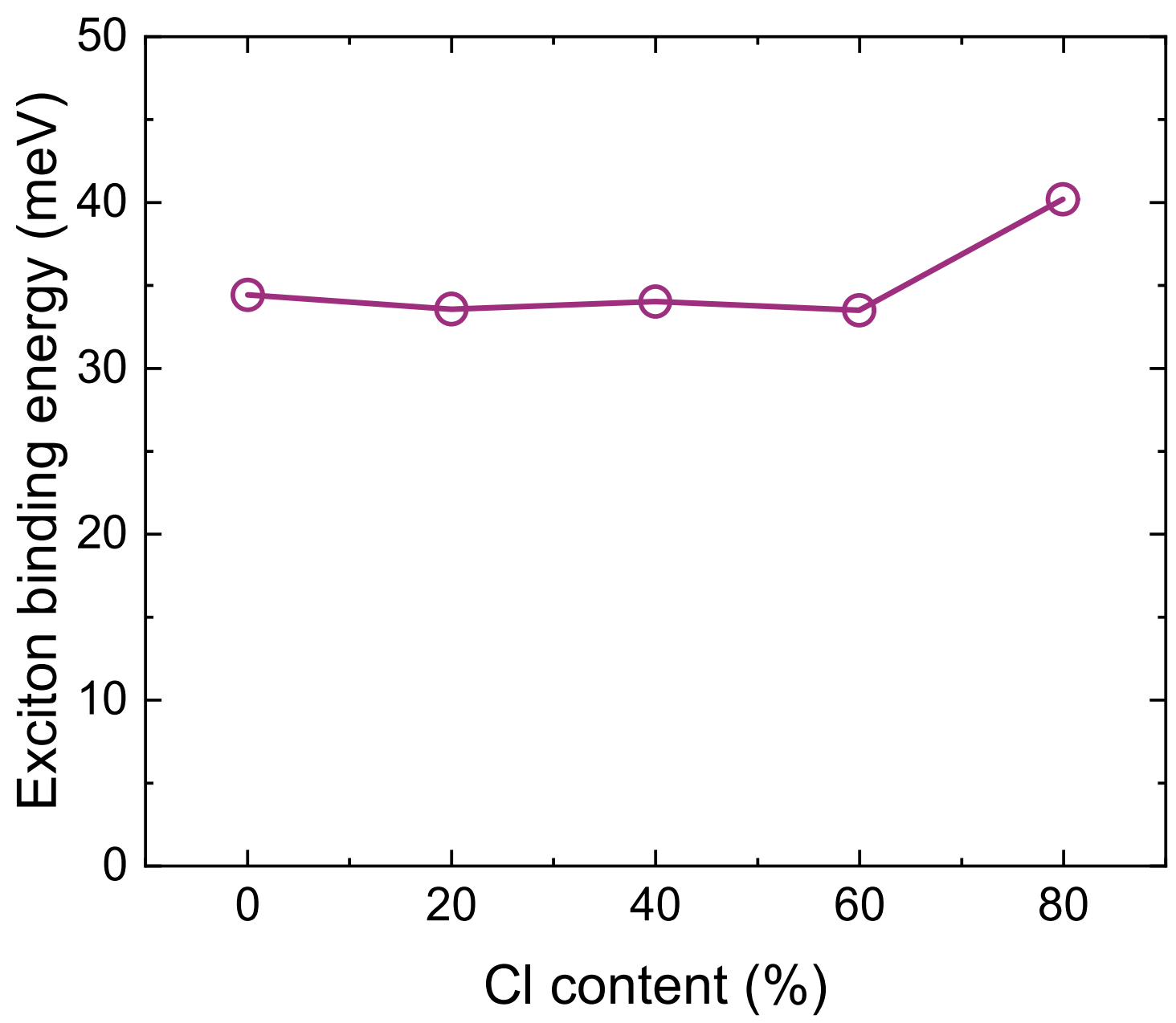

Fig. S4 Exciton binding energies of $FAPbBr_{3-x}Cl_x$ perovskite films containing 0, 20, 40, 60, and 80% Cl relative to the total halide concentration.

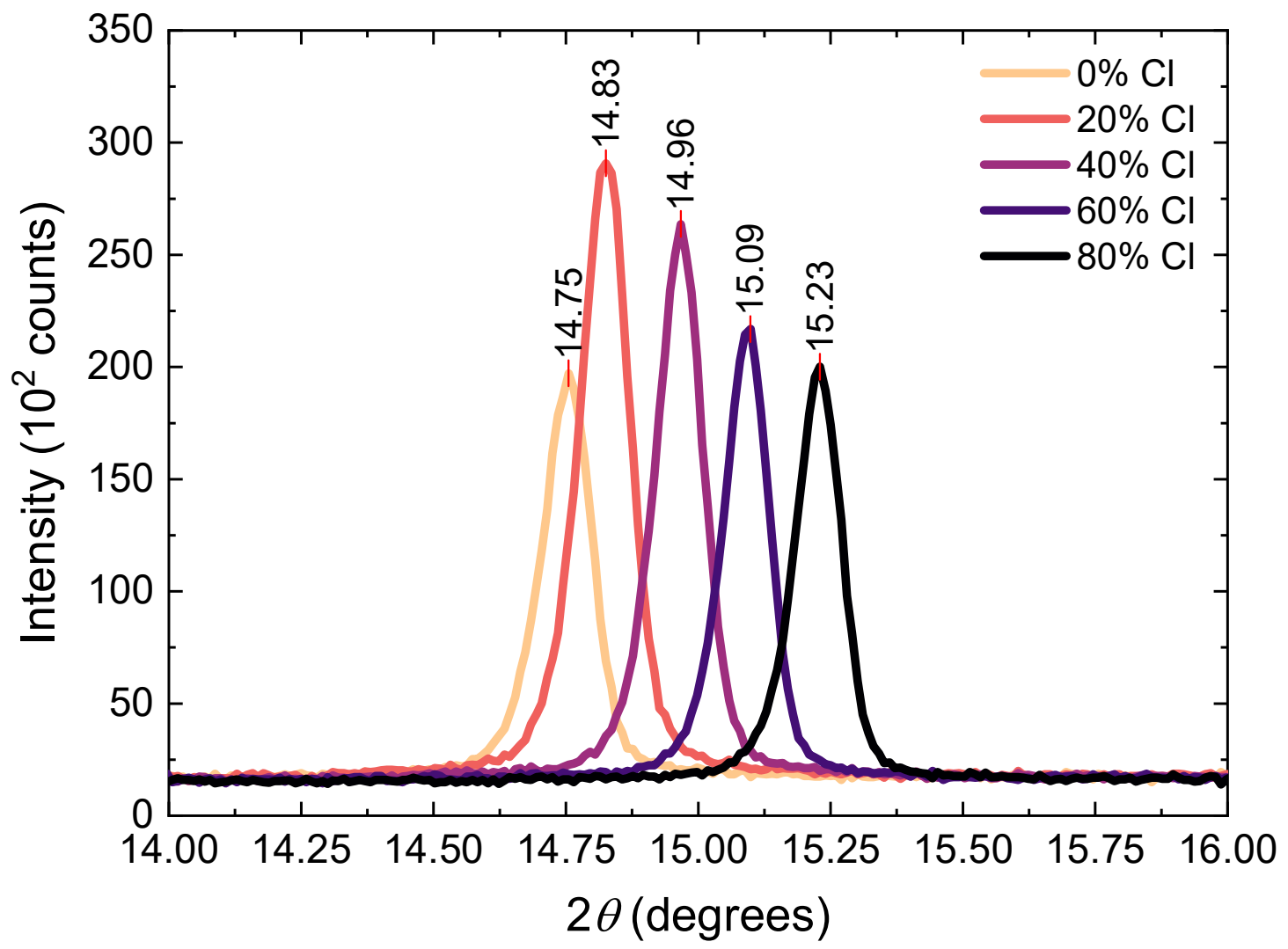

Fig. S5 (100) XRD reflection of cubic $FAPbBr_{3-x}Cl_x$ perovskite films containing 0, 20, 40, 60, and 80% Cl relative to the total halide concentration.

Table S3 Lattice parameter (*a*) of cubic $FAPbBr_{3-x}Cl_x$ perovskite films containing 0, 20, 40, 60, and 80% Cl relative to the total halide concentration, calculated from the (100) XRD reflection peak.

Cl content [%]	(100) ref. [degrees]	Lattice parameter, *a* [Å]
0	14.75	5.99
20	14.83	5.96
40	14.96	5.92
60	15.09	5.87
80	15.23	5.81

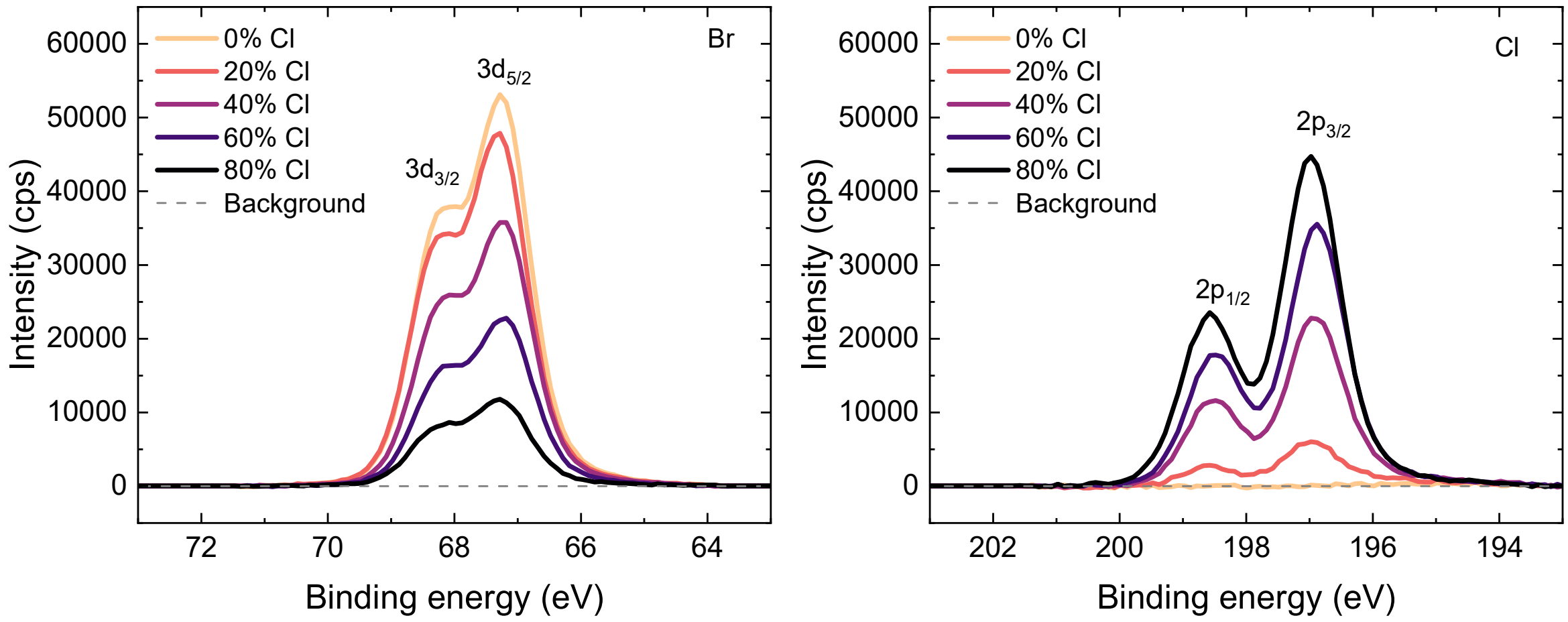

Fig. S6 High-resolution XPS spectra of the Br 3d (left) and Cl 2p (right) core levels for $FAPbBr_{3-x}Cl_x$ perovskite films containing 0, 20, 40, 60, and 80% Cl relative to the total halide concentration.

Table S4 Experimental and expected Br and Cl atomic concentrations determined by XPS for $FAPbBr_{3-x}Cl_x$ perovskite films containing with 0, 20, 40, 60, and 80% Cl relative to the total halide concentration.

Composition	Atomic concentration [%][a]			
	Br		Cl	
	Experimental	Expected	Experimental	Expected
0% Cl	38.8	42.9	0.3	0.0
20% Cl	31.2	34.3	9.8	8.6
40% Cl	21.5	25.7	17.4	17.1
60% Cl	13.1	17.1	25.7	25.7
80% Cl	6.5	8.6	37.0	34.3

[a] Excluding hydrogen.

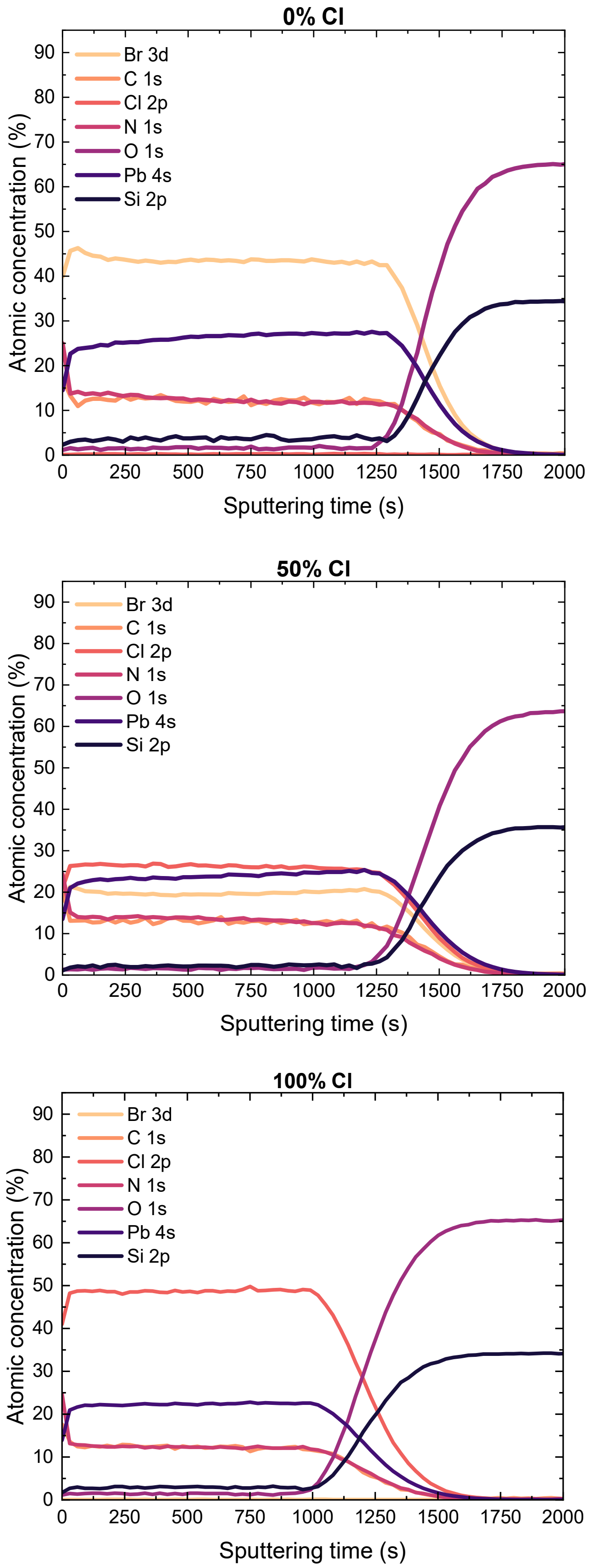

Fig. S7 Depth-profiling XPS of $FAPbBr_{3-x}Cl_x$ perovskite films containing 0, 50, and 100% Cl relative to the total halide concentration.

Table S5 Shockley-Queisser V_{OC}, J_{SC}, FF, and PCE limits for $FAPbBr_{3-x}Cl_x$ perovskite films containing 0, 20, 40, 60, and 80% Cl relative to the total halide concentration.

Cl content [%]	E_g [eV][a]	V_{OC}^{SQ} [V]	J_{SC}^{SQ} [mA cm^{-2}]	FF^{SQ} [-]	PCE^{SQ} [%]
0	2.28	1.94	9.3	0.93	16.8
20	2.38	2.05	7.8	0.93	14.9
40	2.52	2.18	5.9	0.94	12.2
60	2.68	2.33	4.2	0.94	9.2
80	2.85	2.49	2.7	0.94	6.4

[a] From Tauc plot.

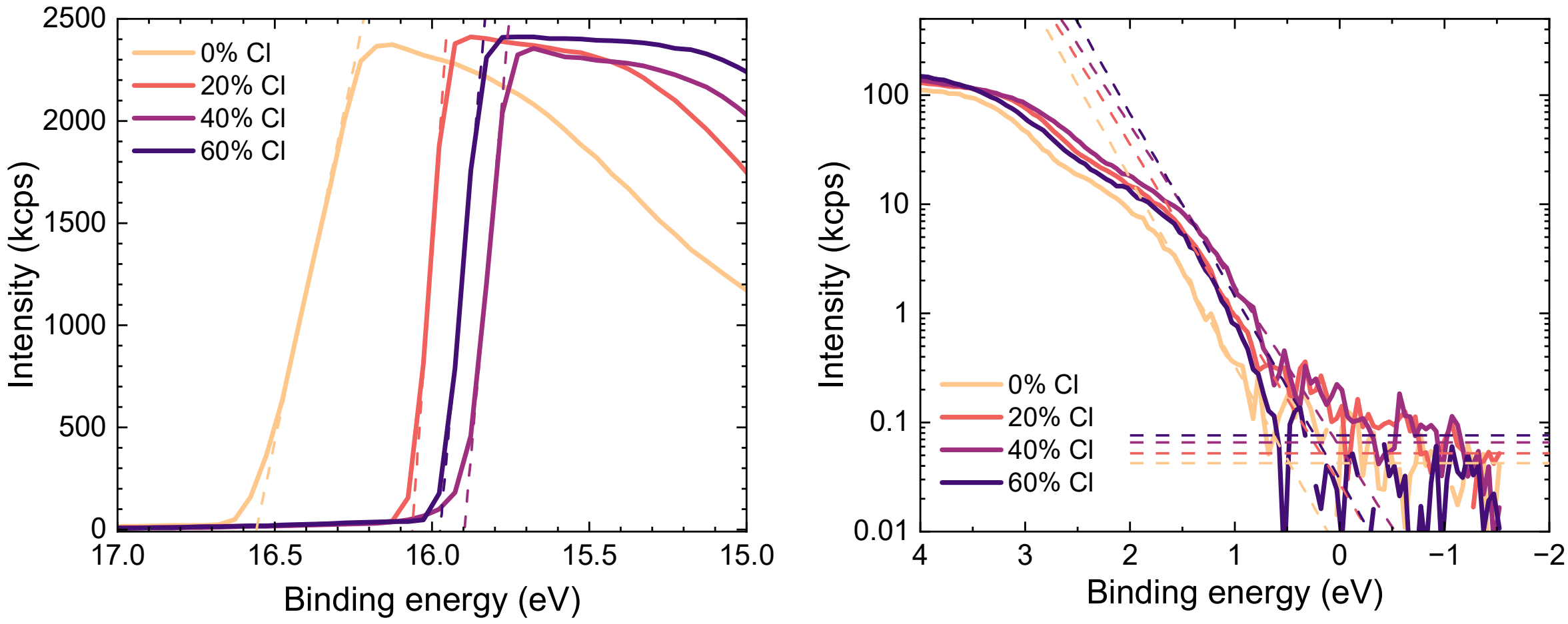

Fig. S8 UPS spectra of $FAPbBr_{3-x}Cl_x$ perovskite films containing 0, 20, 40, 60, and 80% Cl relative to the total halide concentration. Left: Secondary electron cutoff region used to determine the Fermi level (E_F). Right: Low-binding-energy onset displayed on a semi-logarithmic scale, used to determine the valence band maximum (VBM). The binding energy in the spectra is always relative to the Fermi level.

Table S6 Average thickness and roughness values of $FAPbBr_{3-x}Cl_x$ perovskite films containing 0, 20, 40, and 60% Cl relative to the total halide concentration, determined from surface profilometry.

Cl content [%]	Thickness [nm]	Roughness [nm]
0	415 (± 14)	26 (± 4)
20	408 (± 7)	29 (± 1)
40	377 (± 9)	17 (± 1)
60	368 (± 9)	20 (± 1)

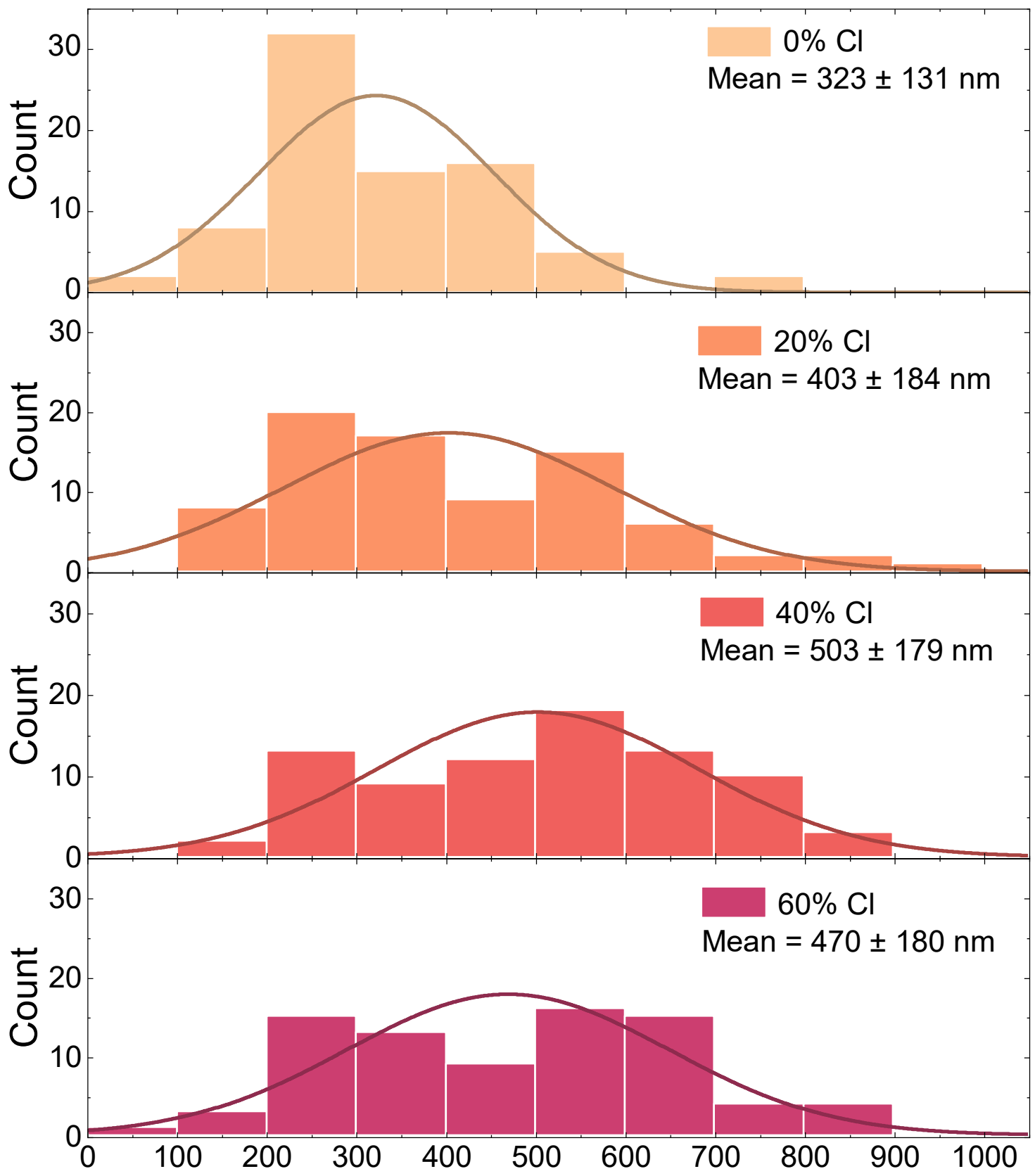

Fig. S9 Grain size distribution in $FAPbBr_{3-x}Cl_x$ perovskite films containing 0, 20, 40, and 60% Cl relative to the total halide concentration, determined from SEM.

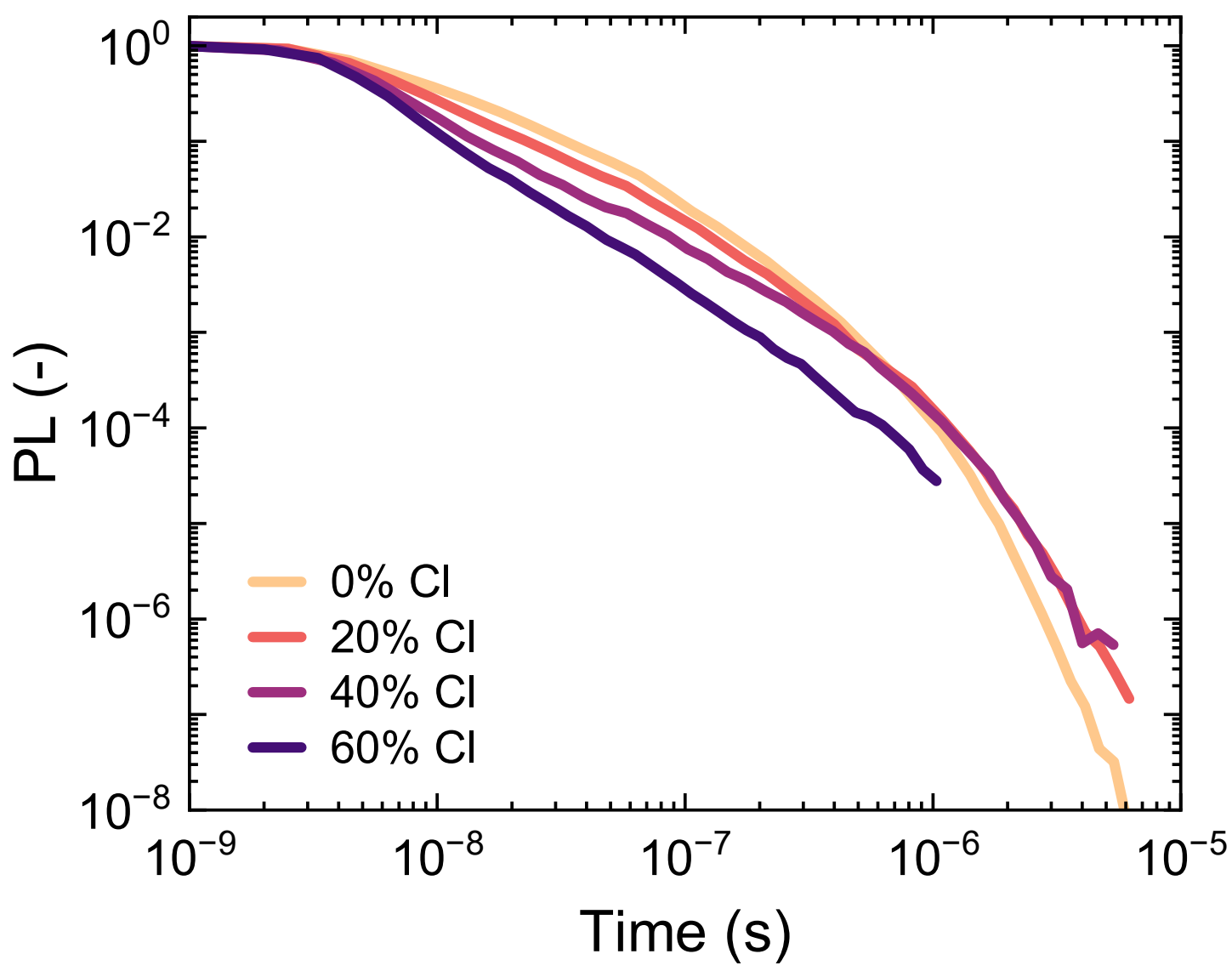

Fig. S10 Normalized PL intensity versus time of $FAPbBr_{3-x}Cl_x$ perovskite films containing 0, 20, 40, and 60% Cl relative to the total halide concentration.

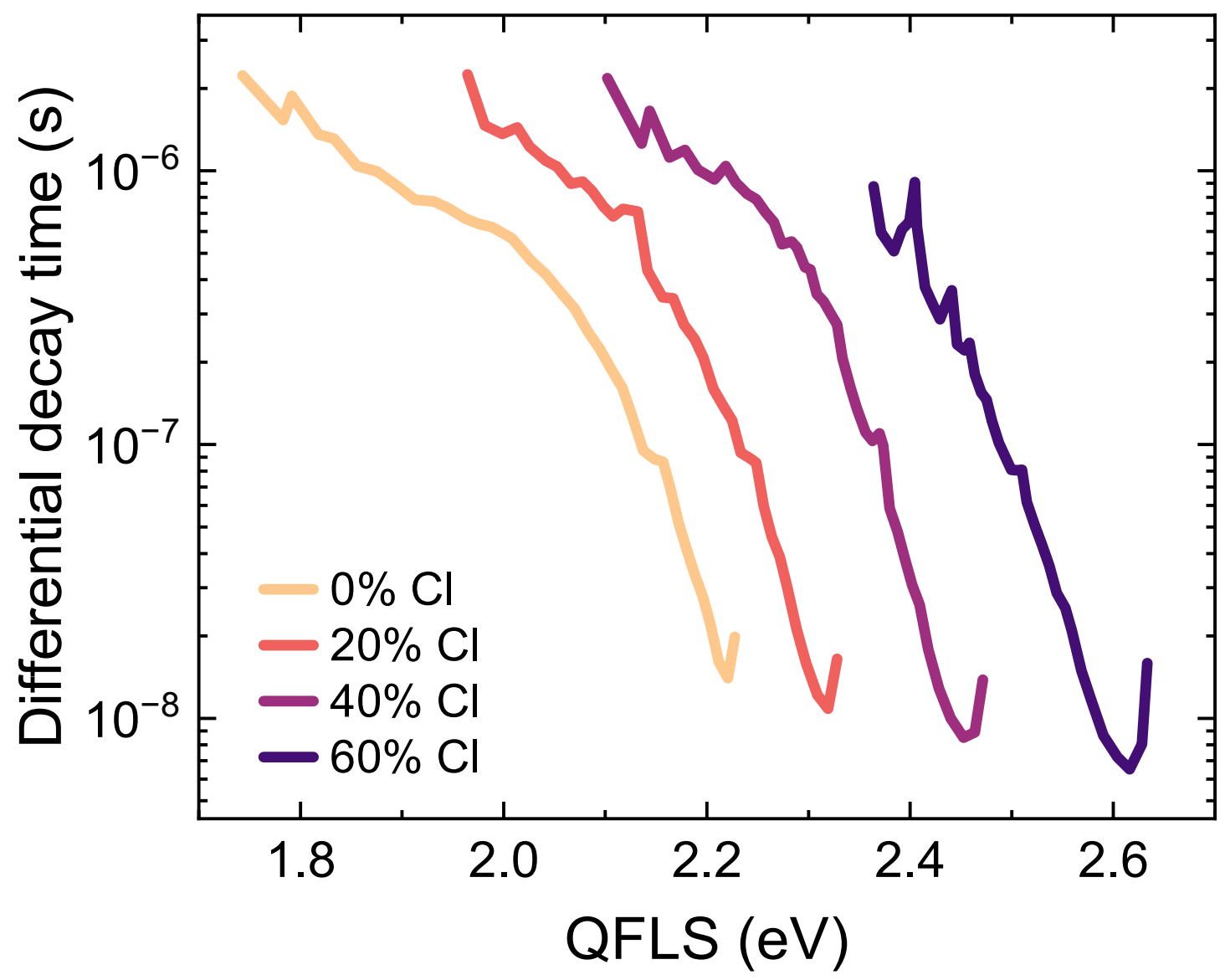

Fig. S11 Differential PL decay time versus QFLS of $FAPbBr_{3-x}Cl_x$ perovskite films containing 0, 20, 40, and 60% Cl relative to the total halide concentration.

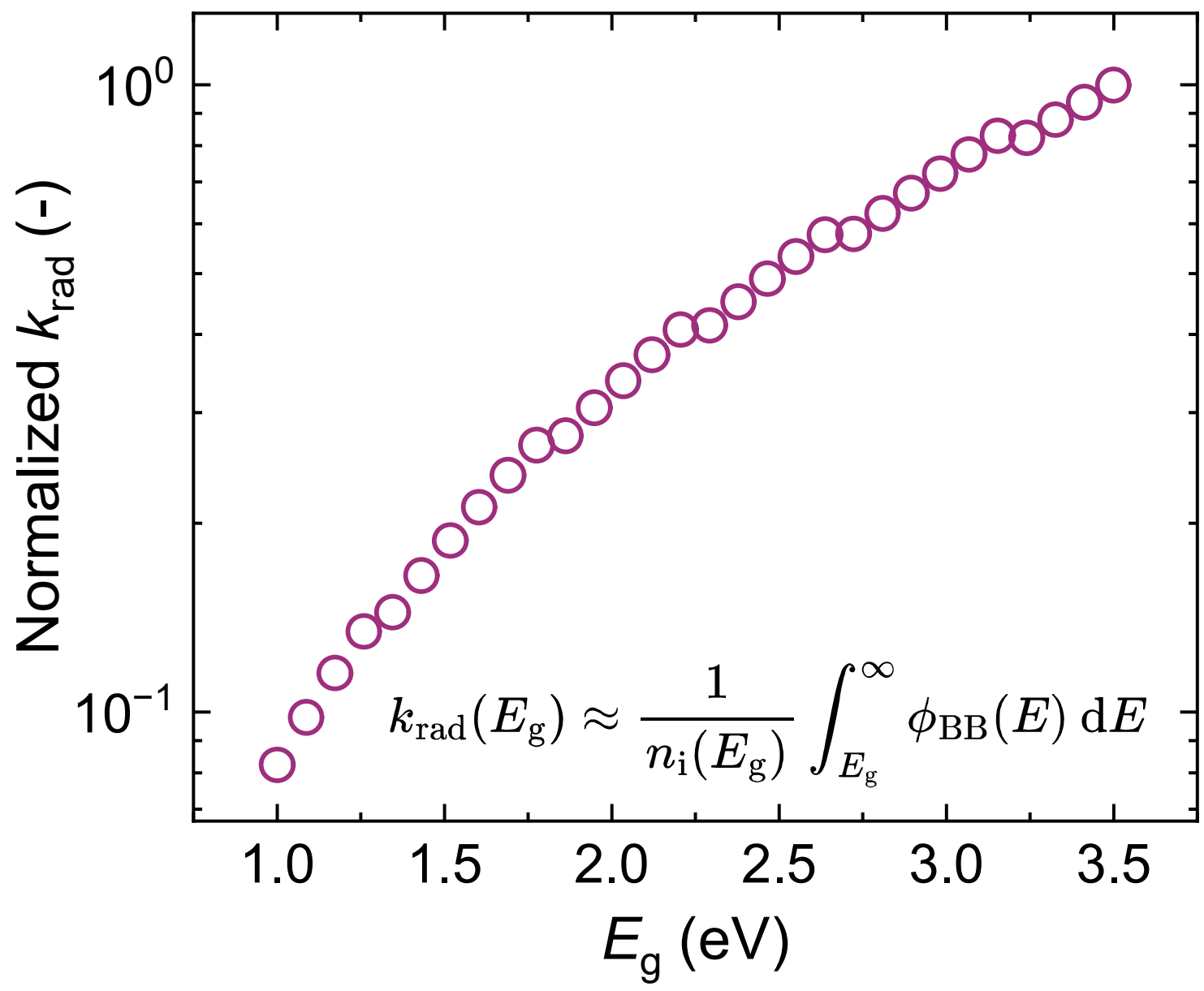

Fig. S12 Normalized radiative recombination coefficient $k_{rad}(E_g)$ calculated as a function of optical bandgap using the Van Roosbroeck-Shockley relation,[66] where n_i is the intrinsic carrier concentration, E_g is the bandgap, ϕ_{BB} is the blackbody emission, and E is the photon energy. Coefficients are calculated under the Shockley-Queisser limit assuming perfect absorption (a = 1) and without corrections for light outcoupling probabilities by excluding the refractive index.

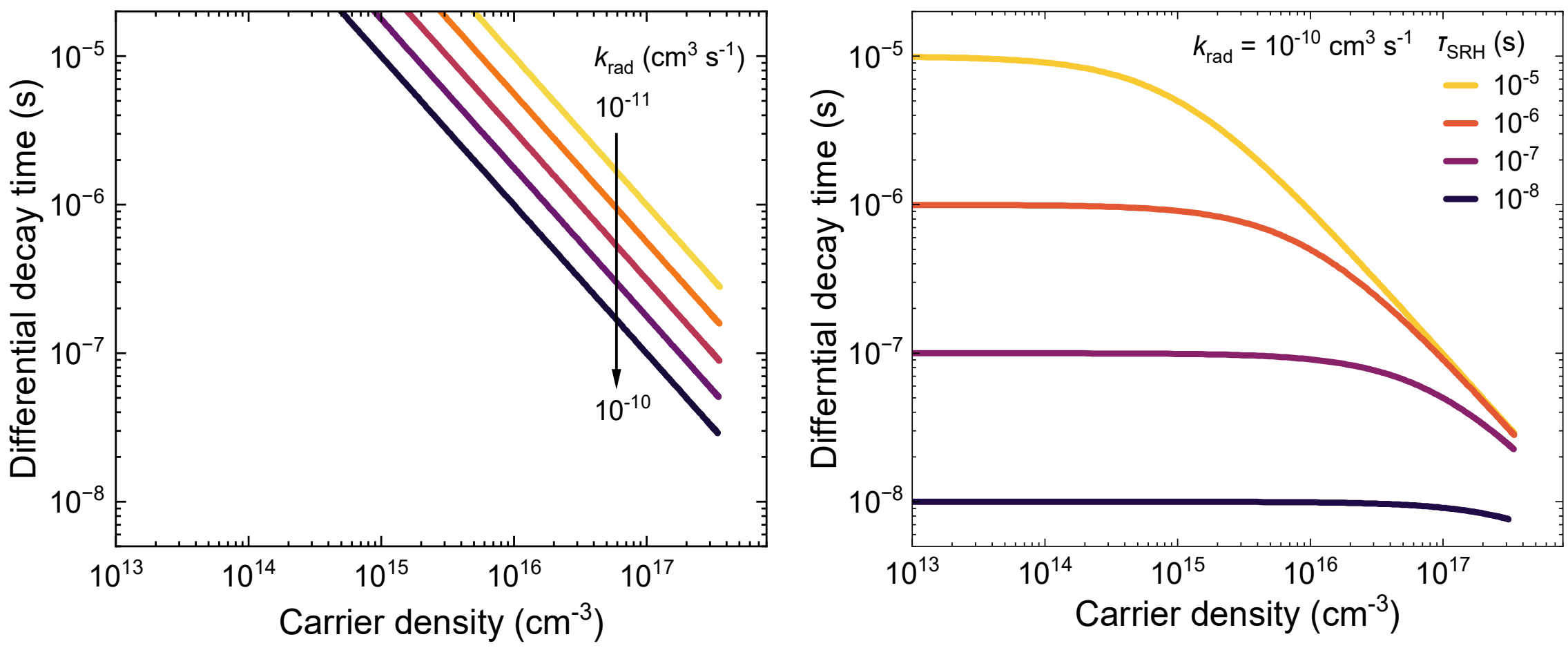

Fig. S13 Simulated differential decay time versus carrier density. Left: Assuming only bimolecular radiative recombination ($dn/dt = - k_{rad}\, n^2$), using various k_{rad} that range from 10^{-10}–10^{-11} cm^3 s^{-1}. Right: Assuming a combination of bimolecular radiative and monomolecular (Shockley-Read-Hall) recombination ($dn/dt = - k_{rad}\, n^2 - n/\tau_{SRH}$) using $k_{rad} = 10^{-10}$ cm^3 s^{-1} and various τ_{SRH} that range from 10^{-5}–10^{-8} s. The horizontal and vertical scales correspond to those in Fig. 3b to enable easier comparison. Comparison with the experimental data (Fig. 3b) shows that the initial shift of the curves with increasing Cl content to lower differential decay times at high constant carrier density seen in Fig. 3b is consistent with a higher k_{rad}. When τ_{SRH} decreases, the shift to lower differential decay times is accompanied by a direct flattening of curves. This is not observed in the experiment (Fig. 3b)

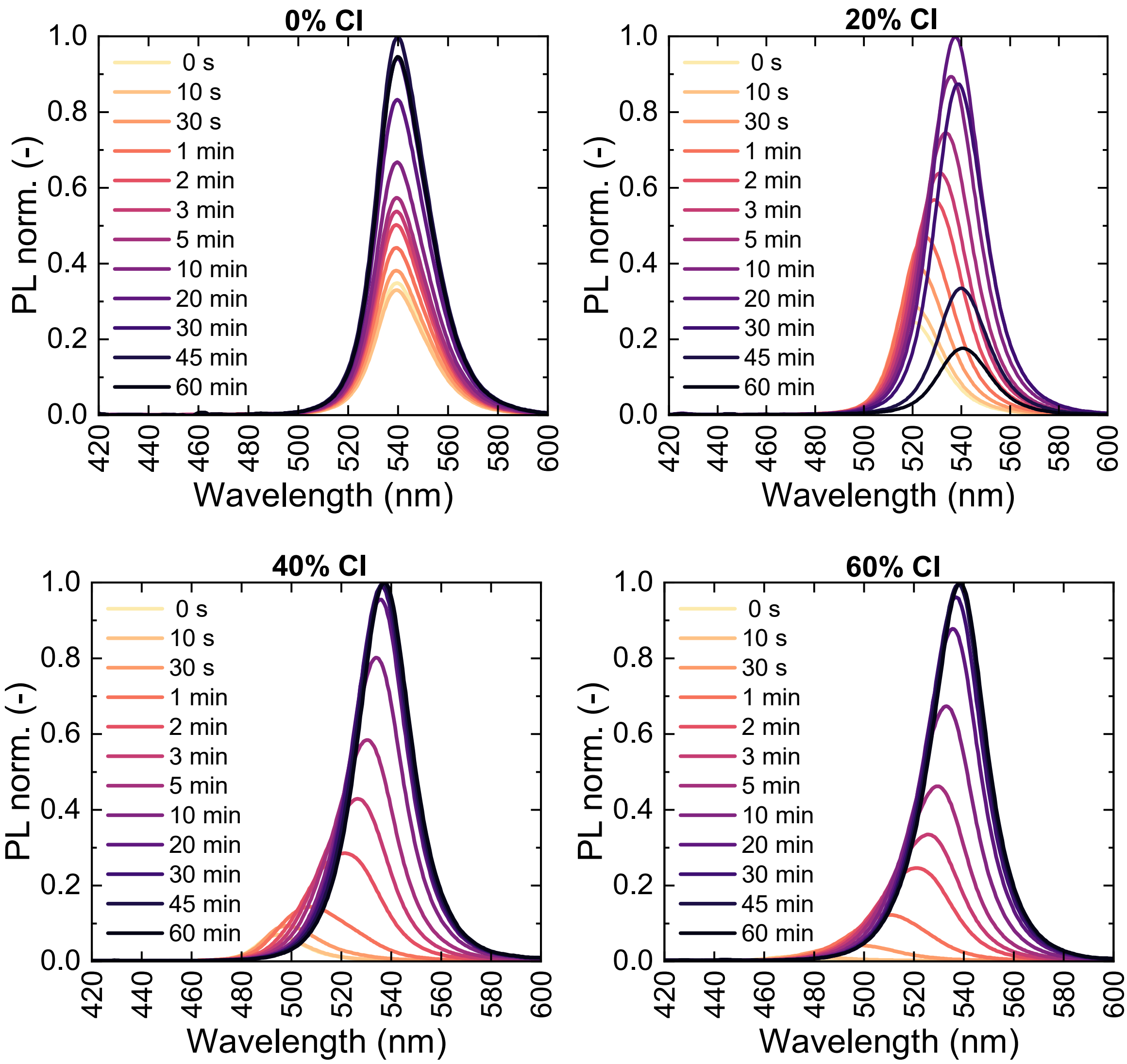

Fig. S14 Normalized PL emission versus time of $FAPbBr_{3-x}Cl_x$ perovskite films containing 0, 20, 40, and 60% Cl relative to the total halide concentration during continuous 365 nm illumination at 1-sun equivalent intensity.

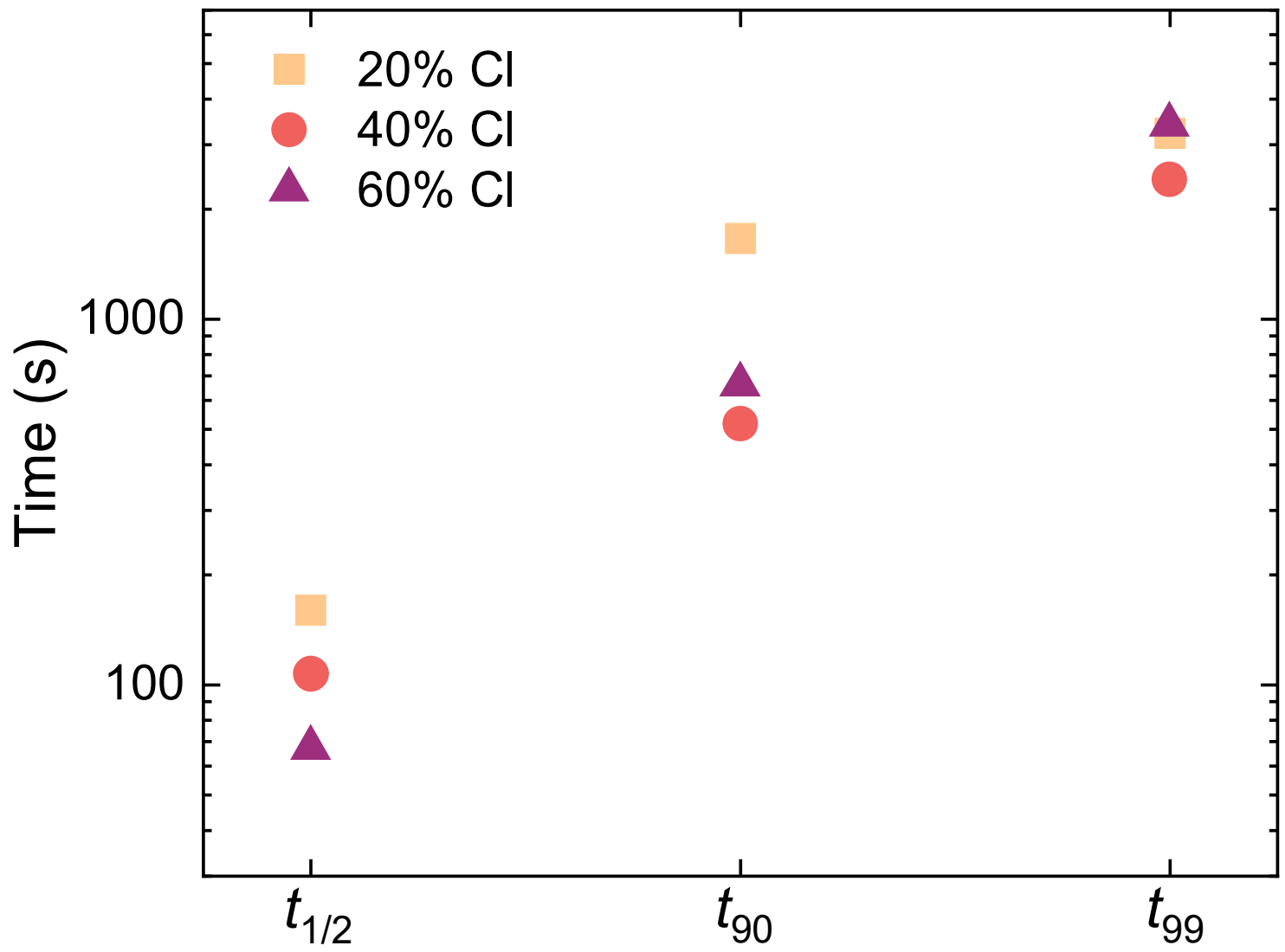

Fig. S15 Extracted light-induced halide segregation times ($t_{1/2}$, t_{90}, and t_{99}) of $FAPbBr_{3-x}Cl_x$ perovskite films containing 20, 40, and 60% Cl relative to the total halide concentration.

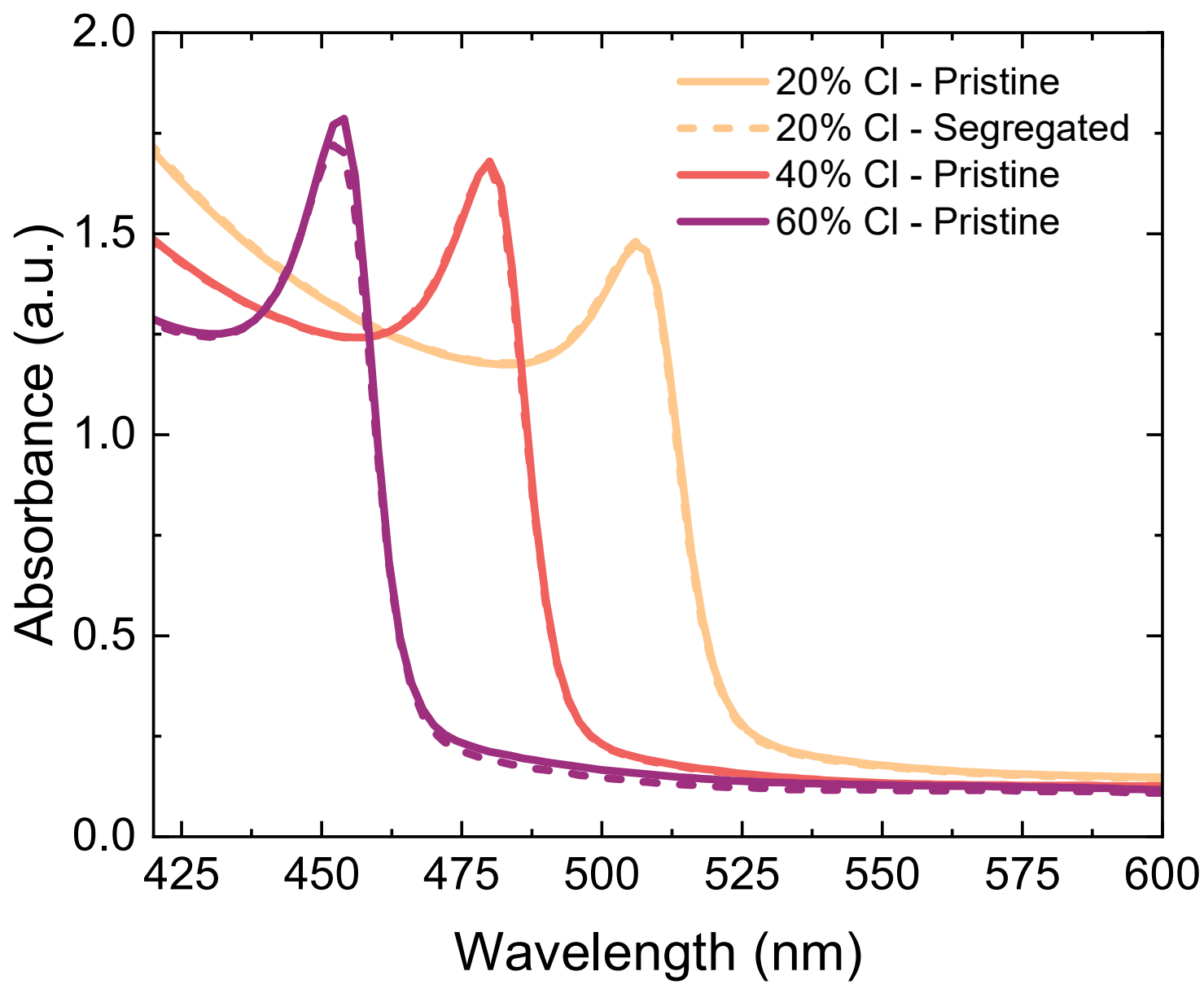

Fig. S16 UV-vis absorbance spectra of $FAPbBr_{3-x}Cl_x$ perovskite films containing 20, 40, and 60% Cl relative to the total halide concentration before illumination (pristine) and immediately after illumination (segregated).

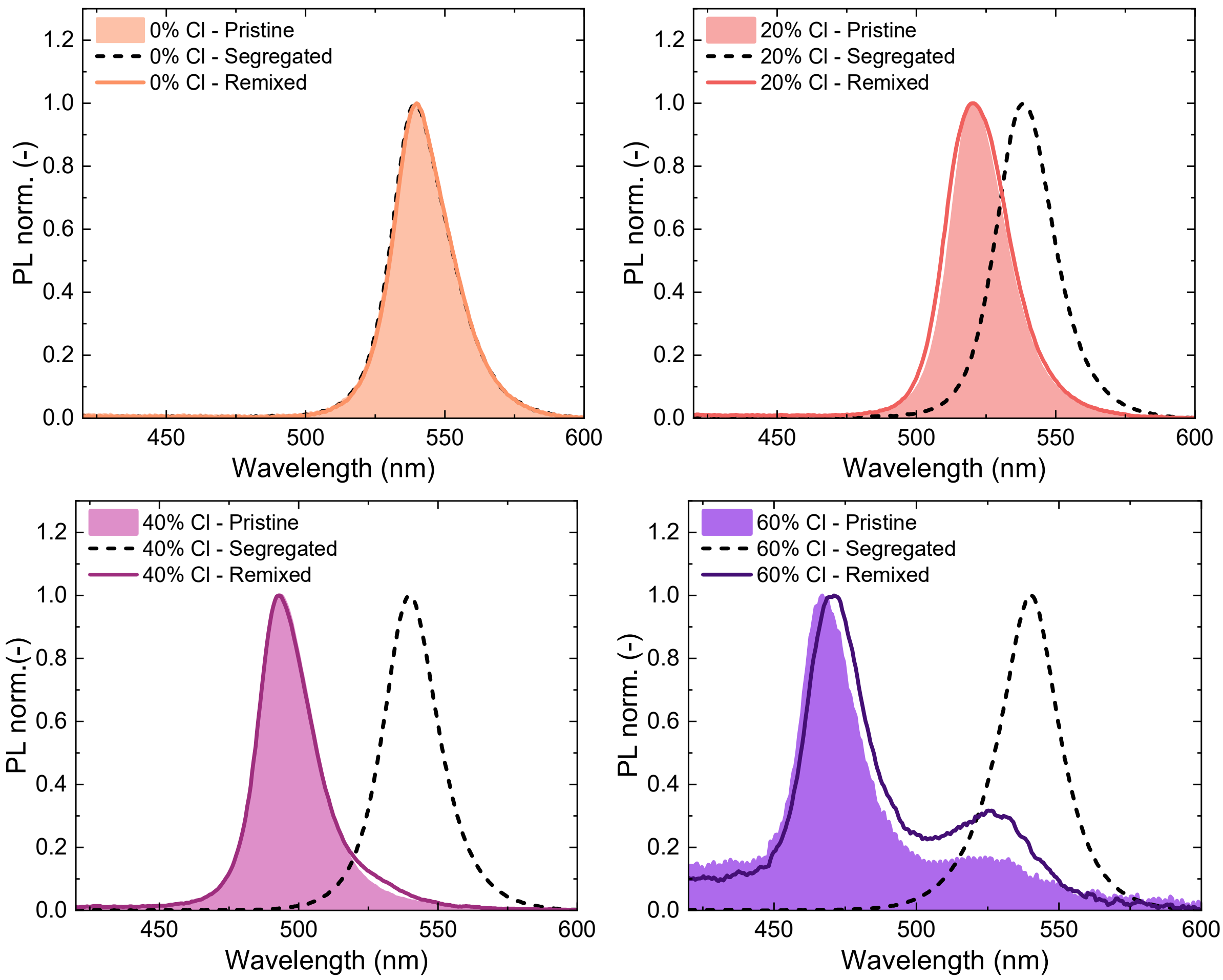

Fig. S17 Normalized PL emission of $FAPbBr_{3-x}Cl_x$ perovskite films containing 0, 20, 40, and 60% Cl relative to the total halide concentration before illumination (pristine), immediately after illumination (segregated), and after 48 h of storage in dark (remixed).

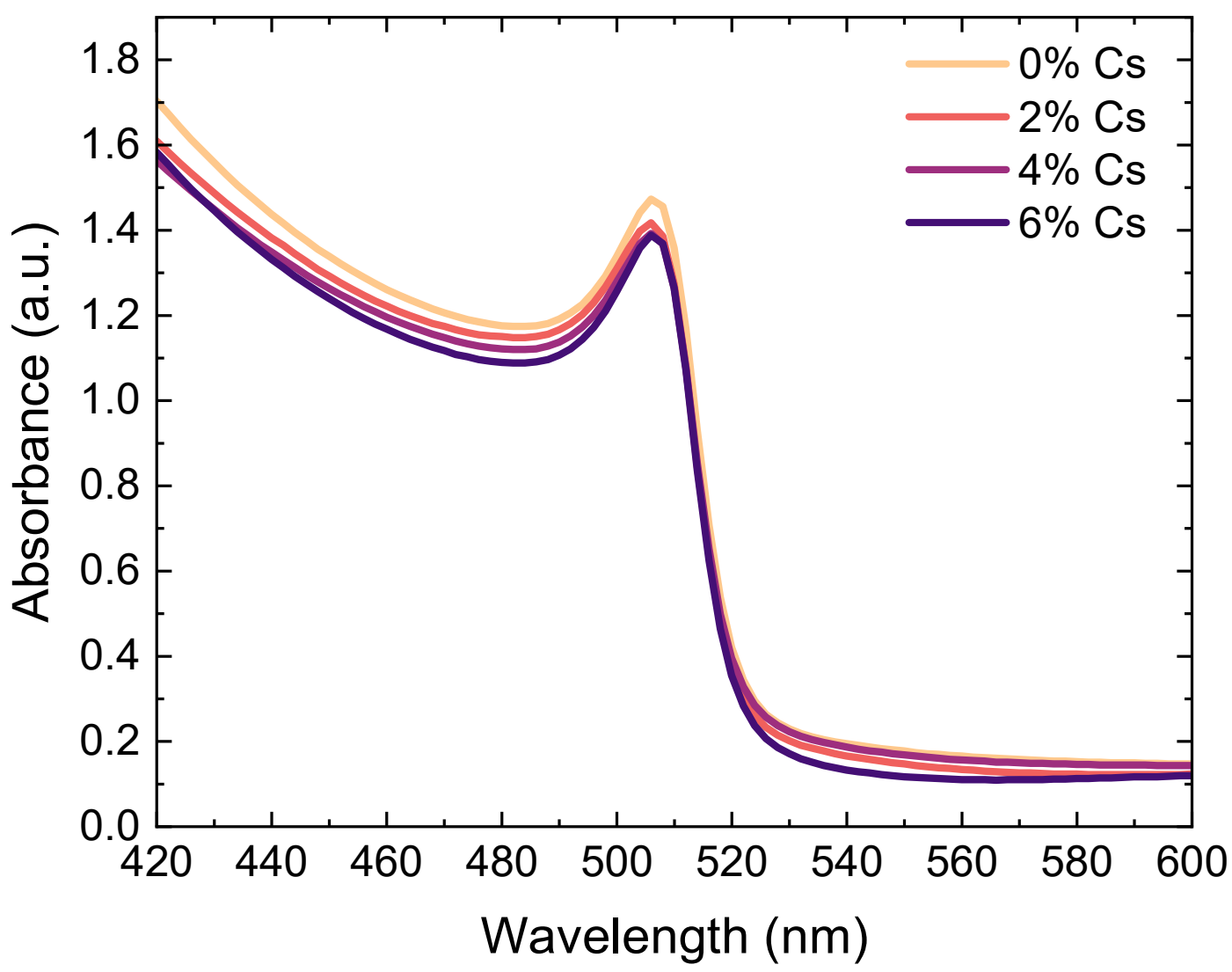

Fig. S18 UV-vis absorbance spectra of $Cs_xFA_{1-x}PbBr_{2.4}Cl_{0.6}$ perovskite films containing 0, 2, 4, and 6% Cs relative to the total A-site cation (FA + Cs) concentration.

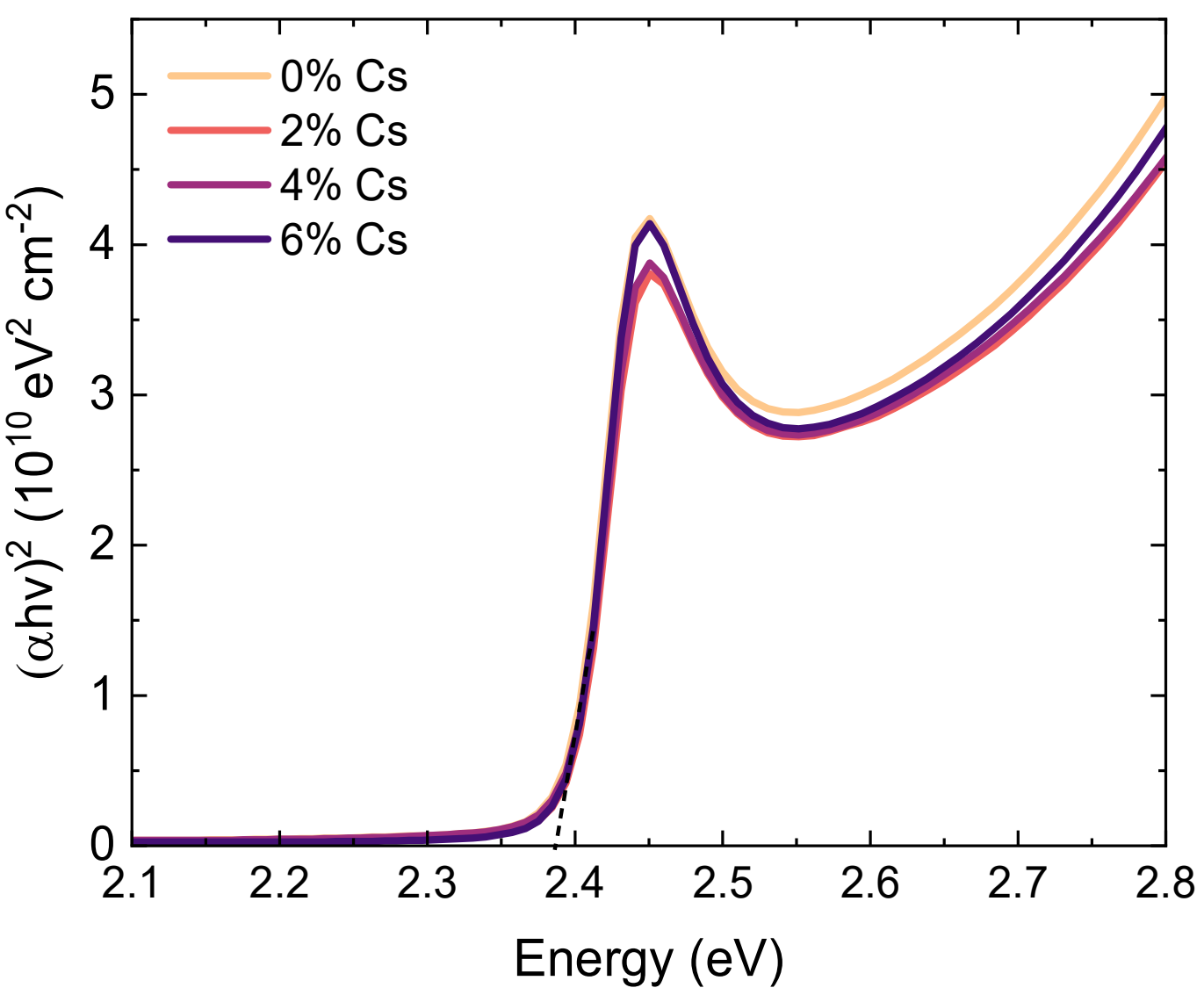

Fig. S19 Tauc plots of $Cs_xFA_{1-x}PbBr_{2.4}Cl_{0.6}$ perovskite films containing 0, 2, 4, and 6% Cs relative to the total A-site cation (FA + Cs) concentration, derived from absorbance spectra.

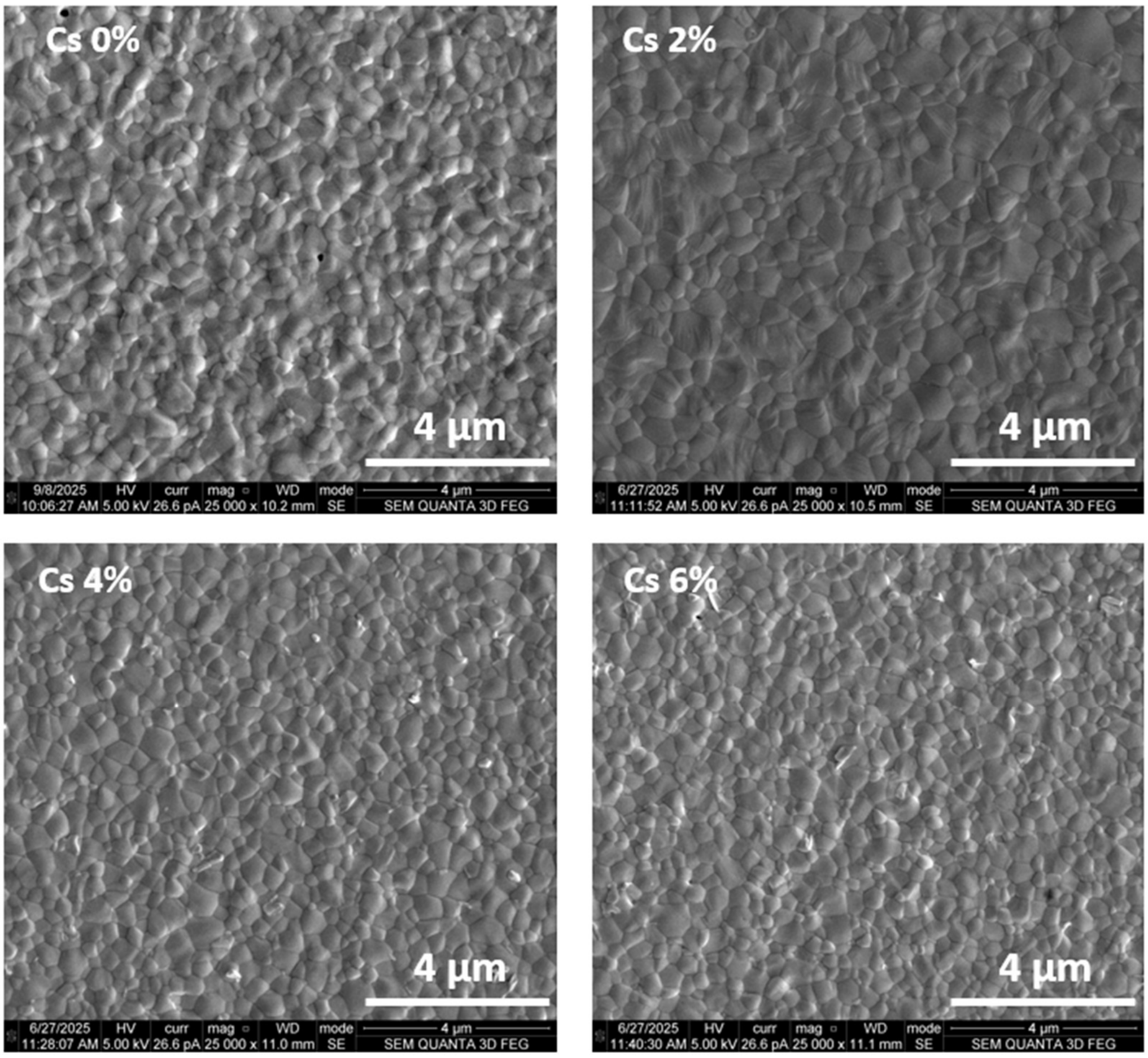

Fig. S20 SEM images of $Cs_xFA_{1-x}PbBr_{2.4}Cl_{0.6}$ perovskite films containing 0, 2, 4, and 6% Cs relative to the total A-site cation (FA + Cs) concentration.

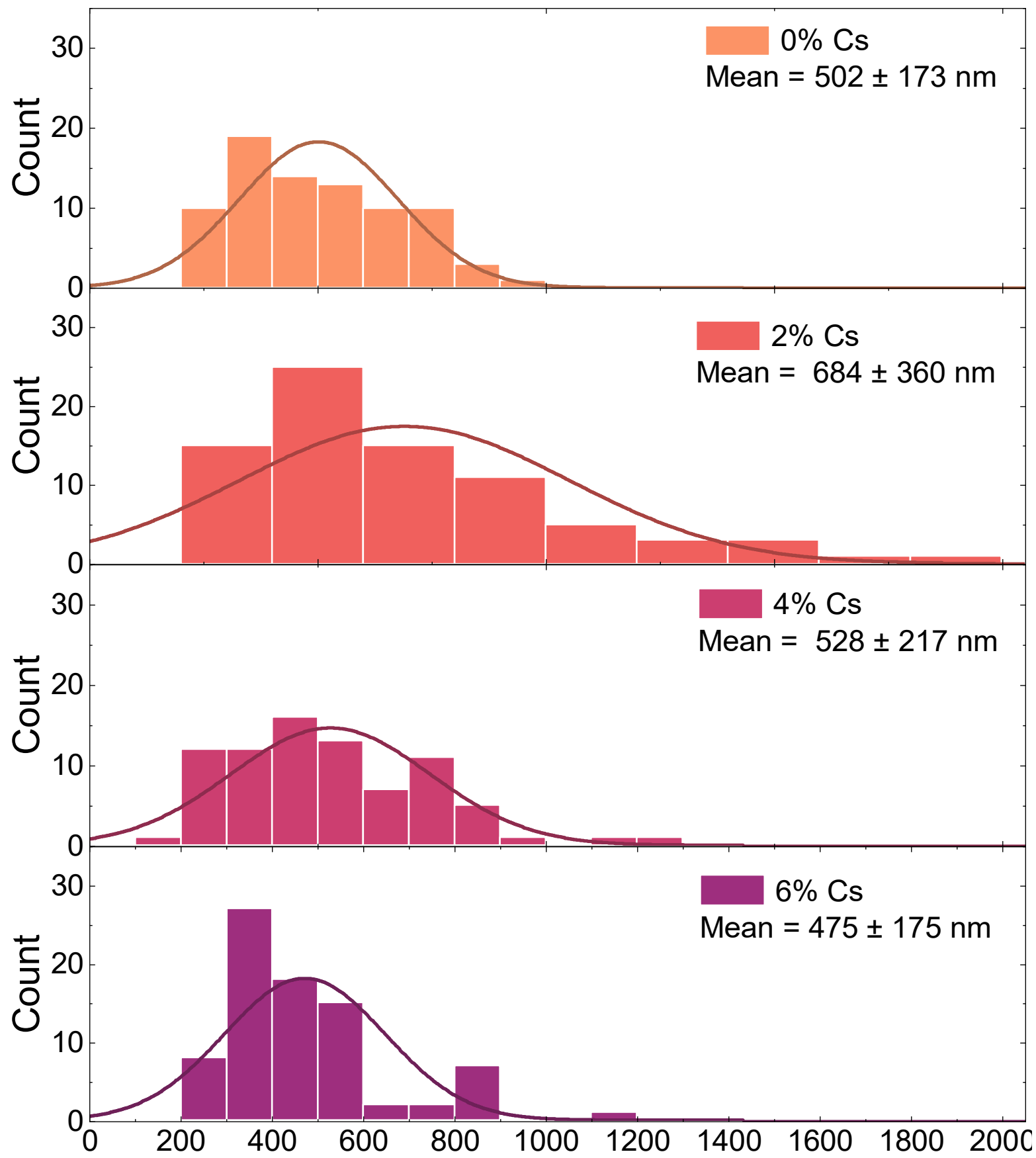

Fig. S21 Grain size distribution in $Cs_xFA_{1-x}PbBr_{2.4}Cl_{0.6}$ perovskite films containing 0, 2, 4, and 6% Cs relative to the total A-site cation (FA + Cs) concentration.

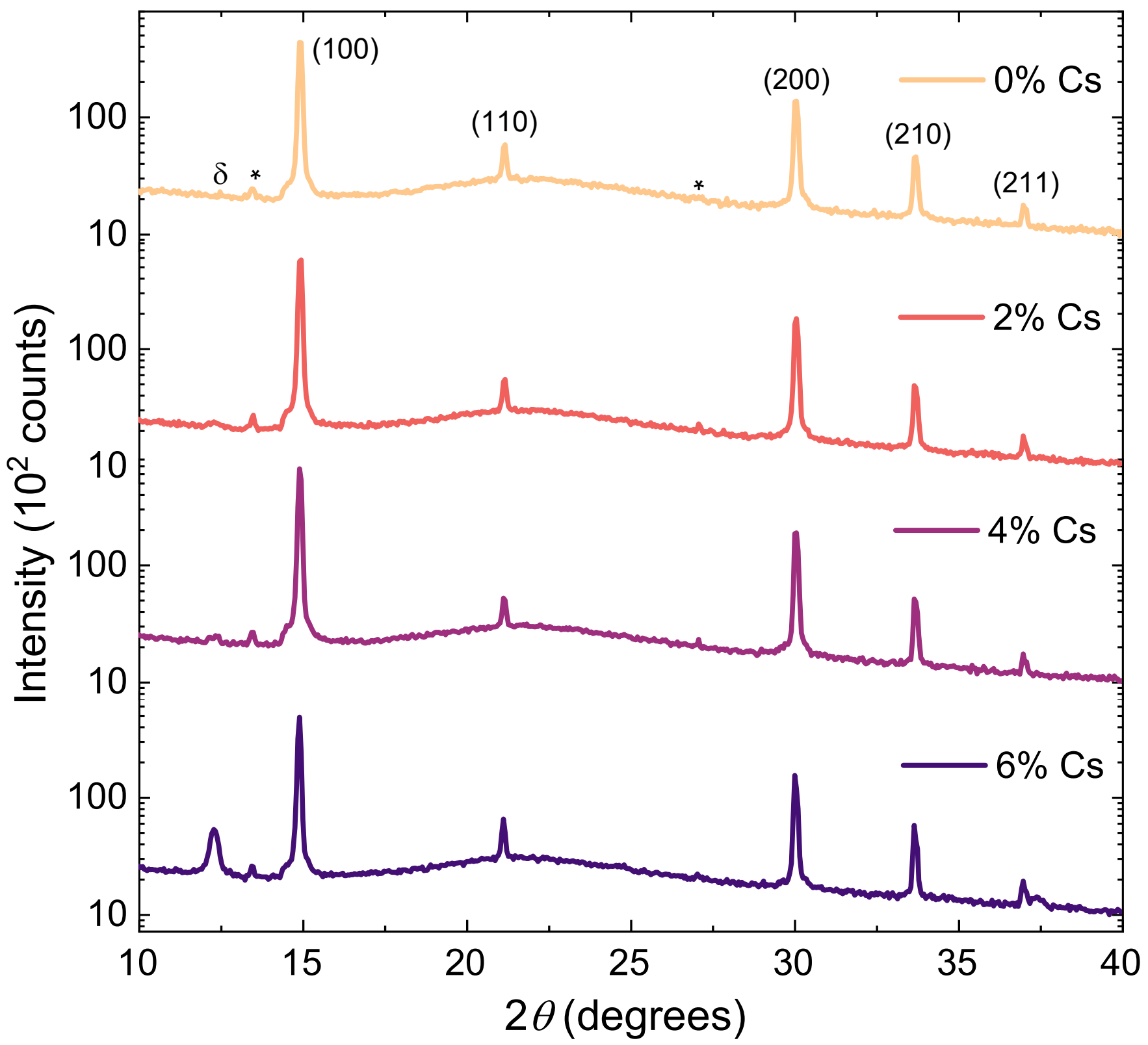

Fig. S22 XRD of $Cs_xFA_{1-x}PbBr_{2.4}Cl_{0.6}$ perovskite films containing 0, 2, 4, and 6% Cs relative to the total A-site cation (FA + Cs) concentration. All diffractograms show the δ-phase of $FAPbBr_3$ at 2θ =12.28° and small unidentified peaks at 2θ = 13.46° and 27.09°, indicated with an asterisk.

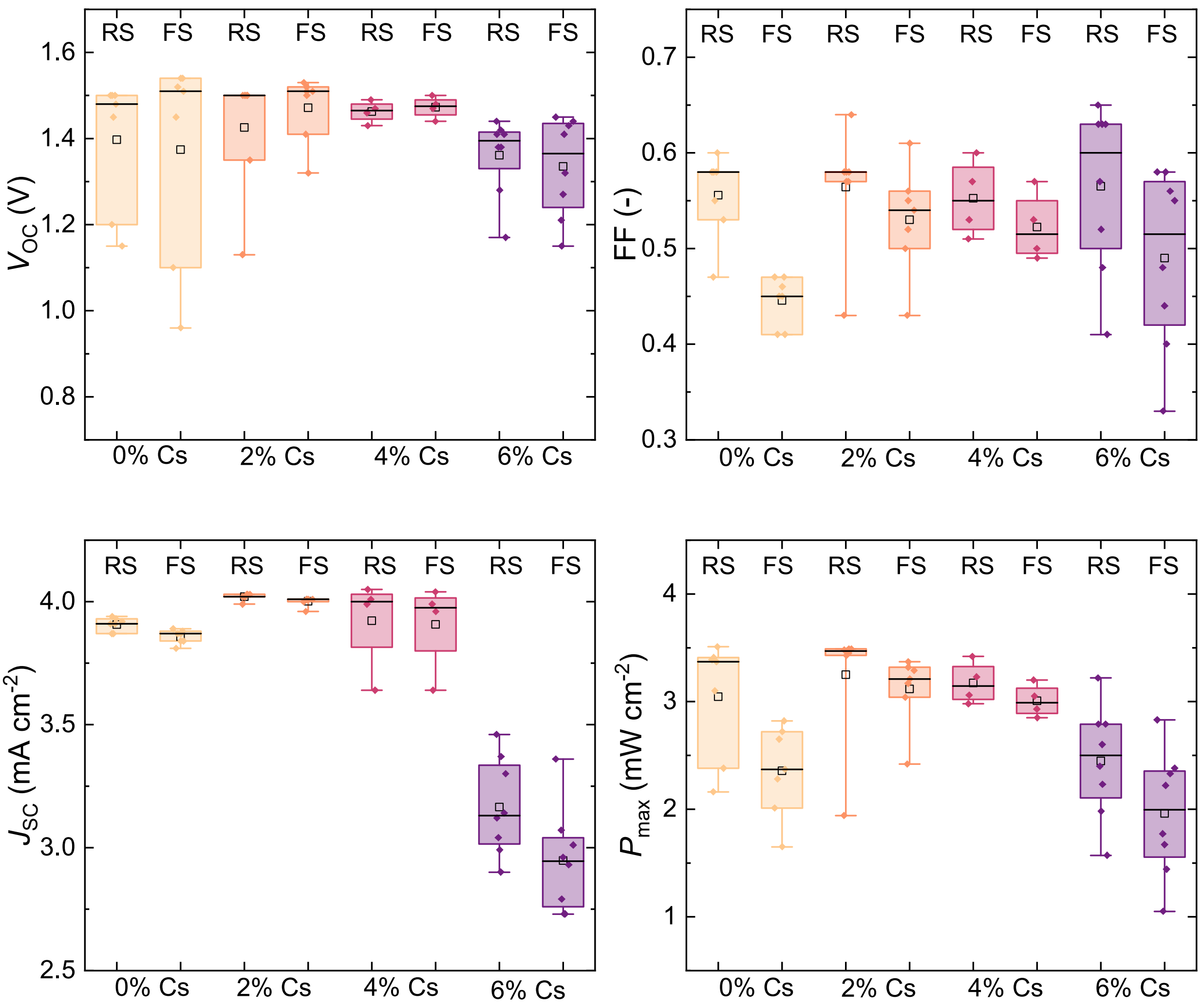

Fig. S23 Box charts reporting *JV* characteristics (V_{OC}, FF, J_{SC}, P_{max}) of $Cs_xFA_{1-x}PbBr_{2.4}Cl_{0.6}$ perovskite solar cells containing 0, 2, 4, and 6% Cs relative to the total A-site cation (FA + Cs) concentration. RS refers to the reverse scan; FS denotes the forward scan direction. The box plots show the mean (blank open square), median (black solid line), 25th and 75th percentiles (box limits), and 5th and 95th percentiles (whiskers). In general the median values are higher than the mean values. This indicates that mean values suffer from few less-efficient devices. The reported maximum power values (P_{max}) are lower than the efficiencies stated in the main text because these initial *JV* characterizations were performed under illumination levels below 1 sun. For the final *JV* data presented in the main text (Table 1), the light intensity was calibrated to the EQE-integrated current obtained under bias illumination. This correction compensates for the insufficient blue spectral content of the light source and enables a reliable determination of the actual PCE at standard 1-sun conditions.

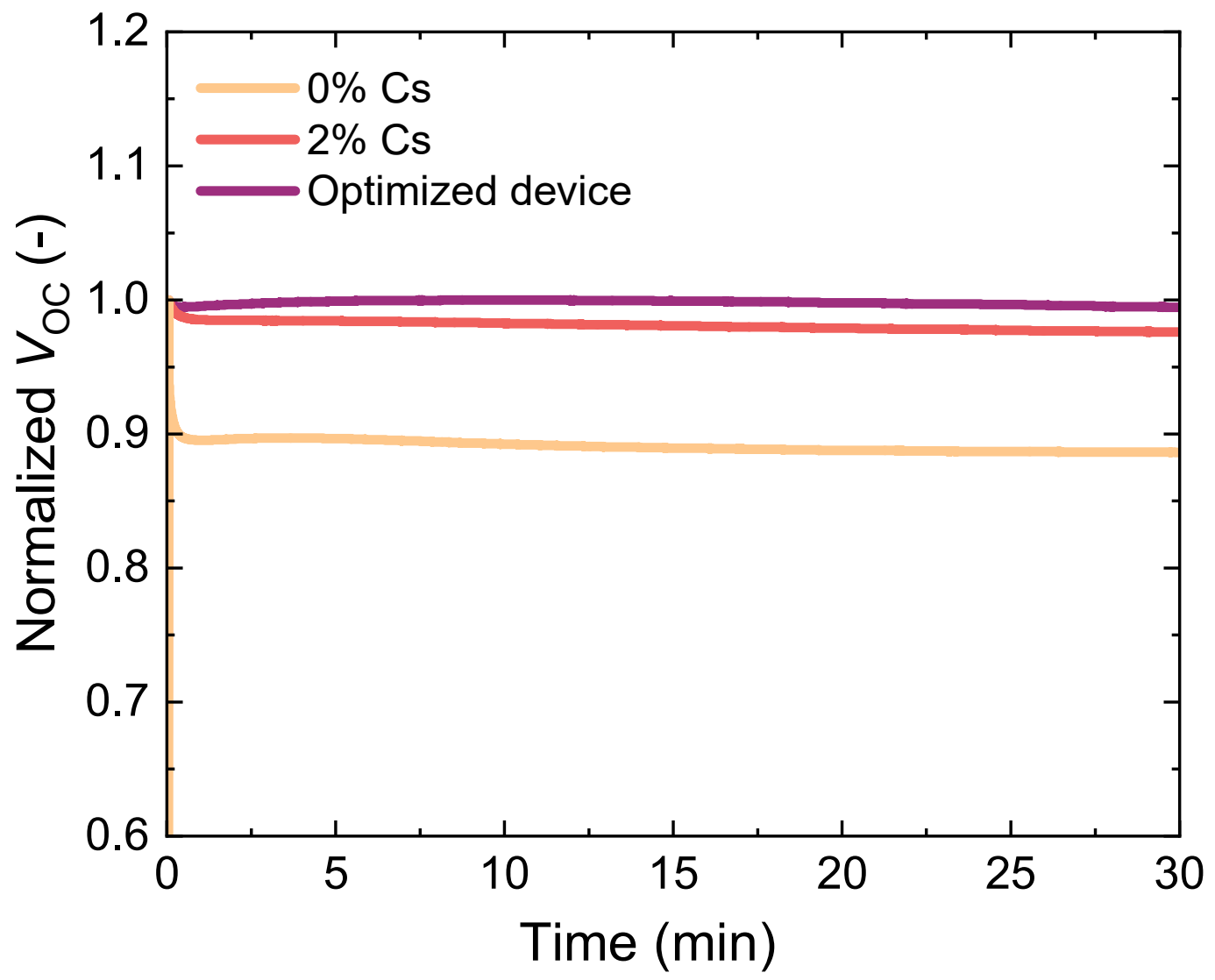

Fig. S24 Normalized V_{OC} tracking of $Cs_xFA_{1-x}PbBr_{2.4}Cl_{0.6}$ perovskite solar cells processed without (x = 0) and with 2% Cs (x = 0.02) incorporation in the bulk, and of the optimized device processed with F-PEACl top surface passivation and with a 1:1:1 (w/w/w) mixture of PCBM, CMC, and ICBA as ETL.

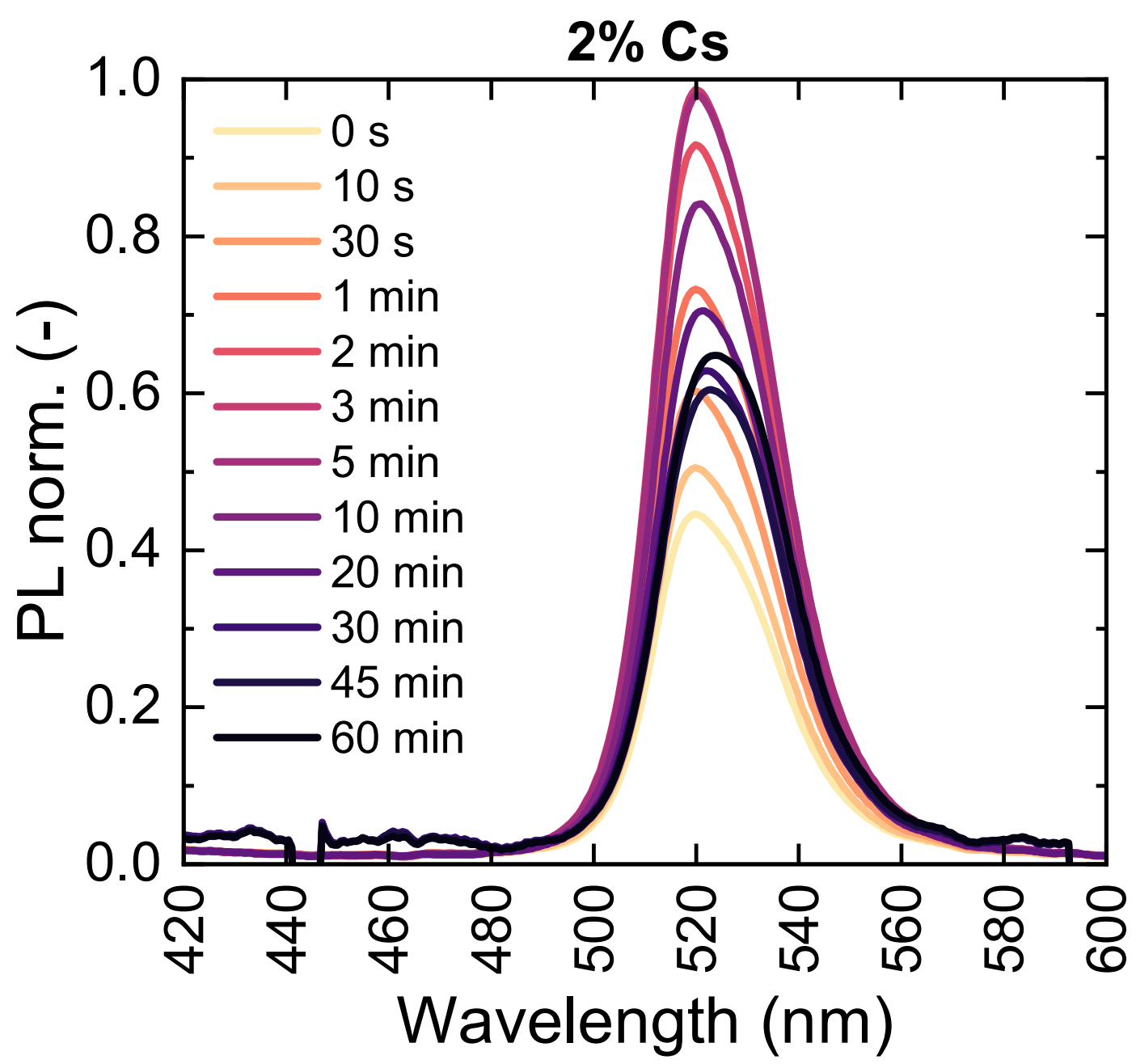

Fig. S25 Normalized PL emission versus time of $Cs_{0.02}FA_{0.98}PbBr_{2.4}Cl_{0.6}$ perovskite films during continuous 365 nm illumination at 1-sun equivalent intensity.

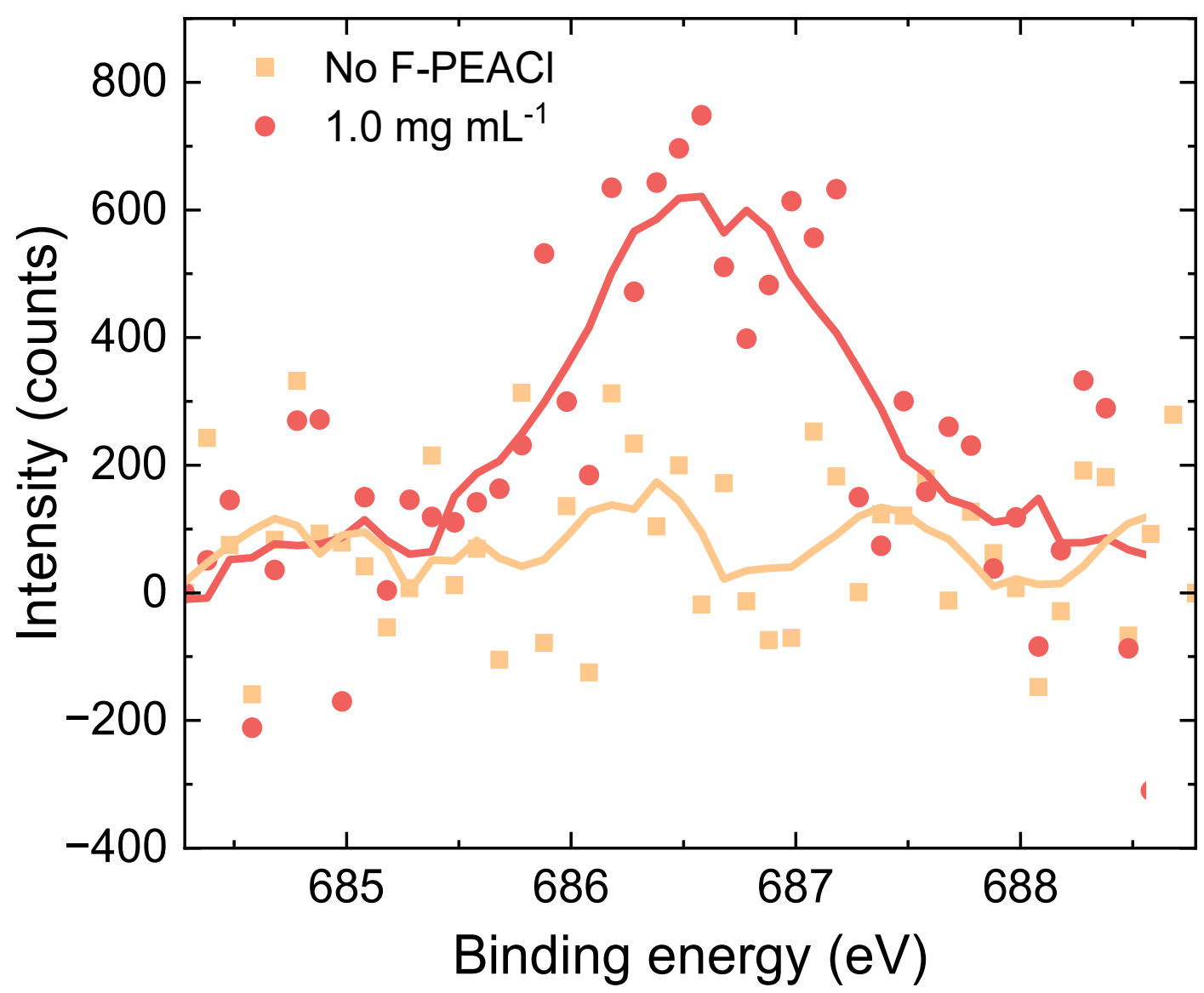

Fig. S26 XPS F 1s signal (solid markers) of $Cs_{0.02}FA_{0.98}PbBr_{2.4}Cl_{0.6}$ perovskite films processed without and with 1.0 mg mL^{-1} F-PEACl top surface passivation. Solid lines are obtained with Savitzky-Golay filtering.

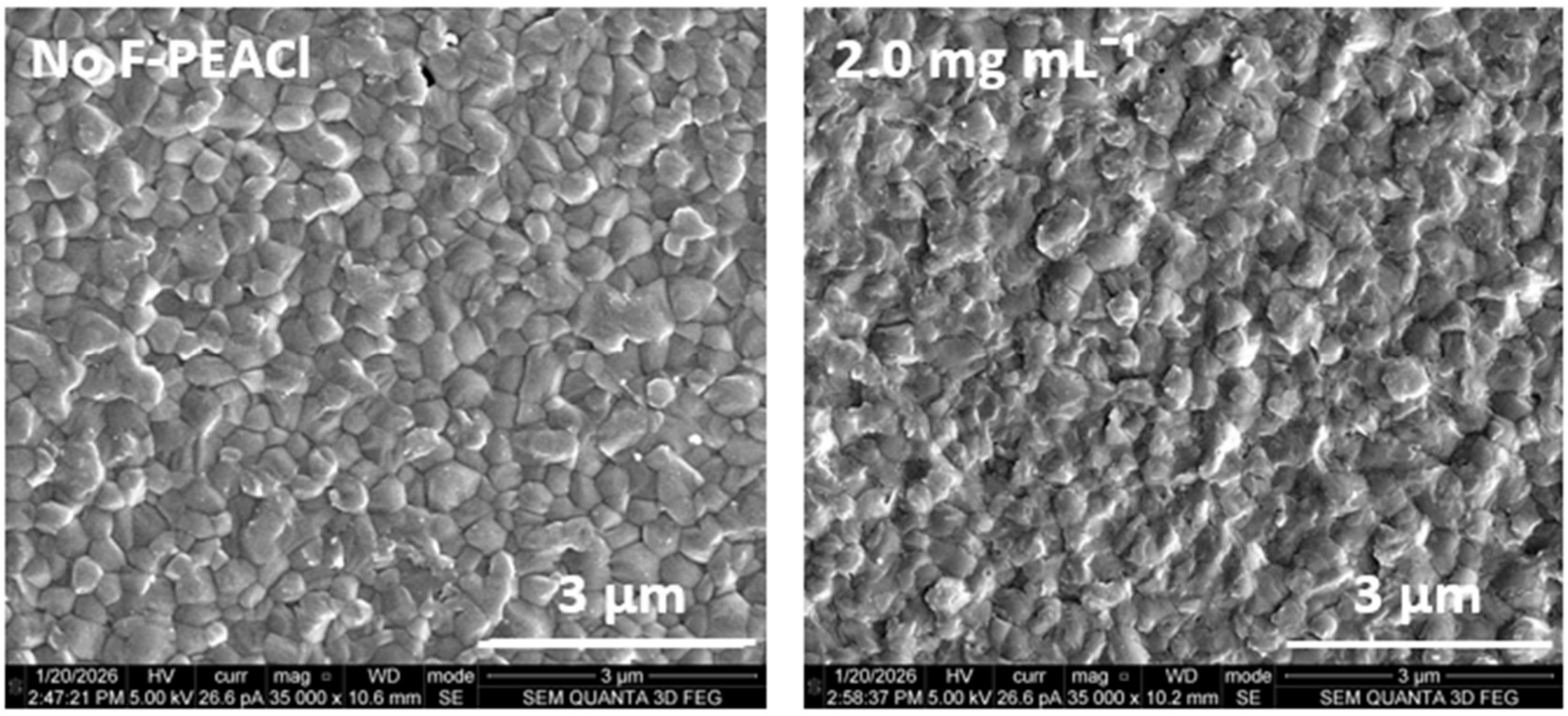

Fig. S27 SEM images of $Cs_{0.02}FA_{0.98}PbBr_{2.4}Cl_{0.6}$ perovskite films processed without and with 2.0 mg mL^{-1} F-PEACl top surface passivation.

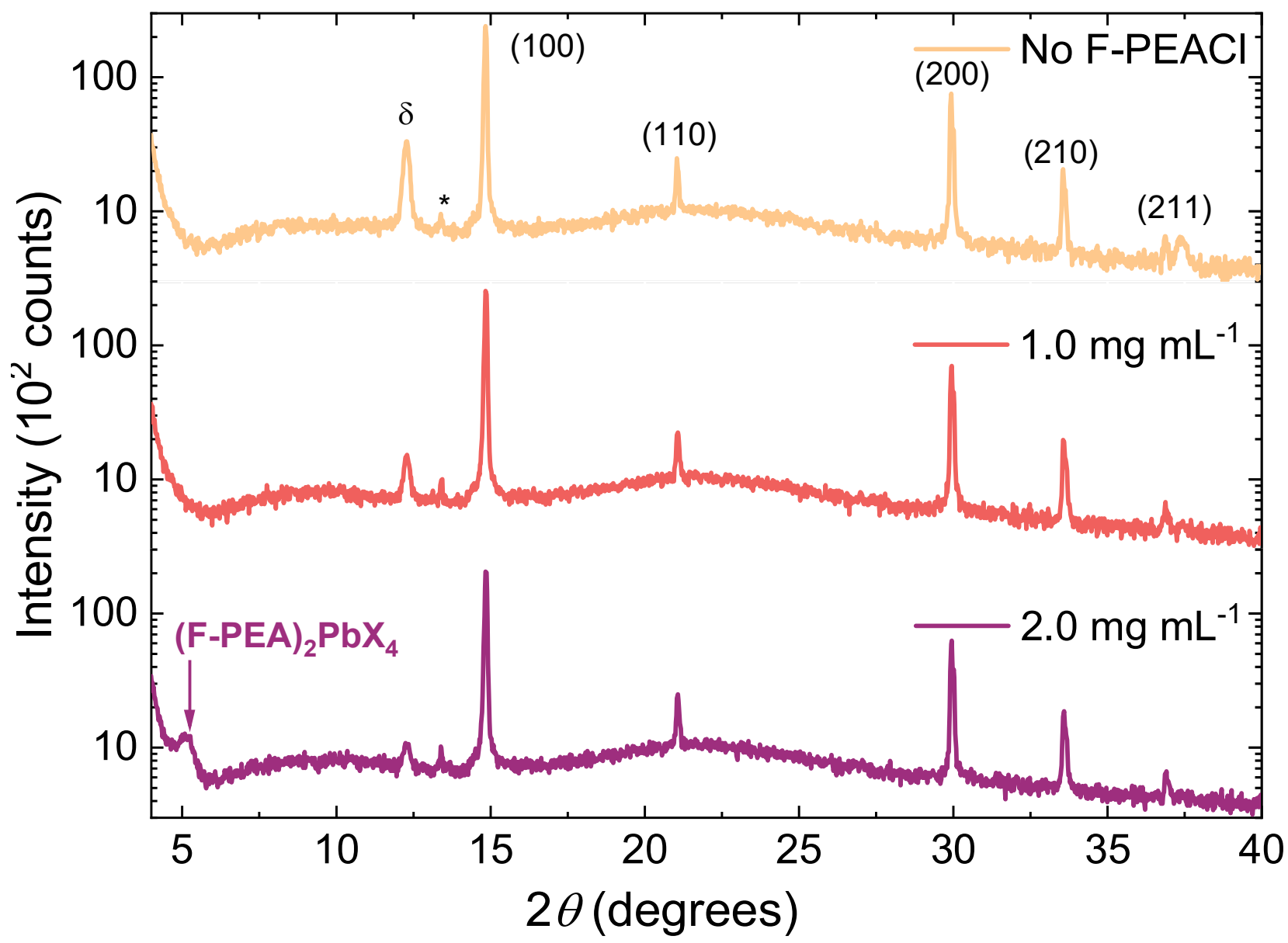

Fig. S28 XRD of $Cs_{0.02}FA_{0.98}PbBr_{2.4}Cl_{0.6}$ perovskite films processed without and with 1.0 and 2.0 mg mL^{-1} F-PEACl surface top passivation. All diffractograms show the δ-phase of $FAPbBr_3$ at 2θ =12.28° and a small unidentified peak at 13.46°, indicated with an asterisk. The peak of $(F\text{-}PEA)_2PbX_4$ is located at 5.10°.

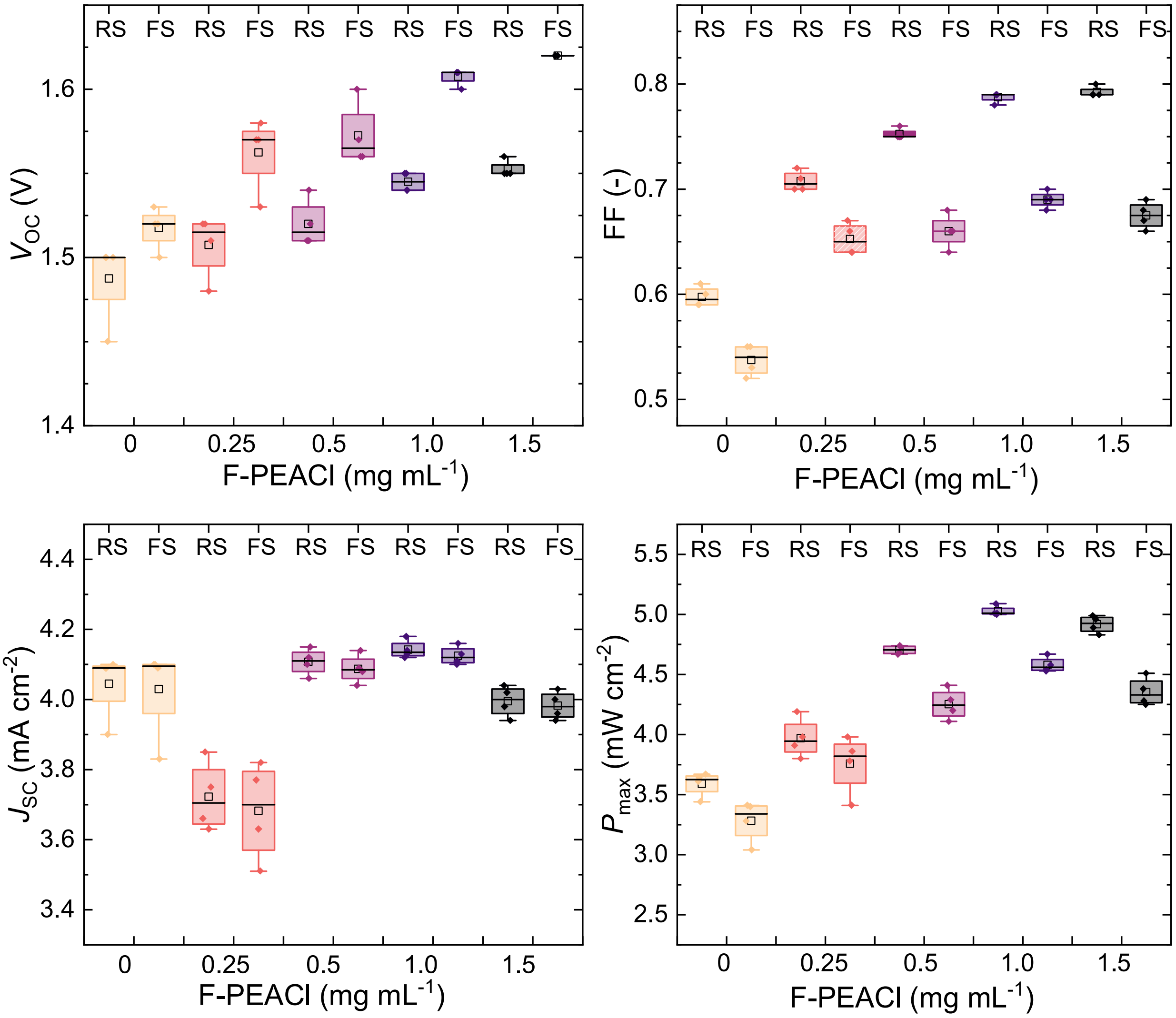

Fig. S29 Box charts reporting *JV* characteristics (V_{OC}, FF, J_{SC}, P_{max}) of $Cs_{0.02}FA_{0.98}PbBr_{2.4}Cl_{0.6}$ perovskite solar cells processed without and with F-PEACl surface top passivation. Concentrations of 0.25, 0.5, 1.0, and 1.5 mg mL^{-1} were employed. RS refers to the reverse scan; FS denotes the forward scan direction. The box plots show the mean (blank open square), median (black solid line), 25th and 75th percentiles (box limits), and 5th and 95th percentiles (whiskers). The reported maximum power values (P_{max}) are lower than the efficiencies stated in the main text (Table 1) because these initial *JV* characterizations were performed under illumination levels below 1 sun. For the final *JV* data presented in the text, the light intensity was calibrated to the EQE-integrated current obtained under bias illumination. This correction compensates for the insufficient blue spectral content of the light source and enables a reliable determination of the actual PCE at standard 1-sun conditions.

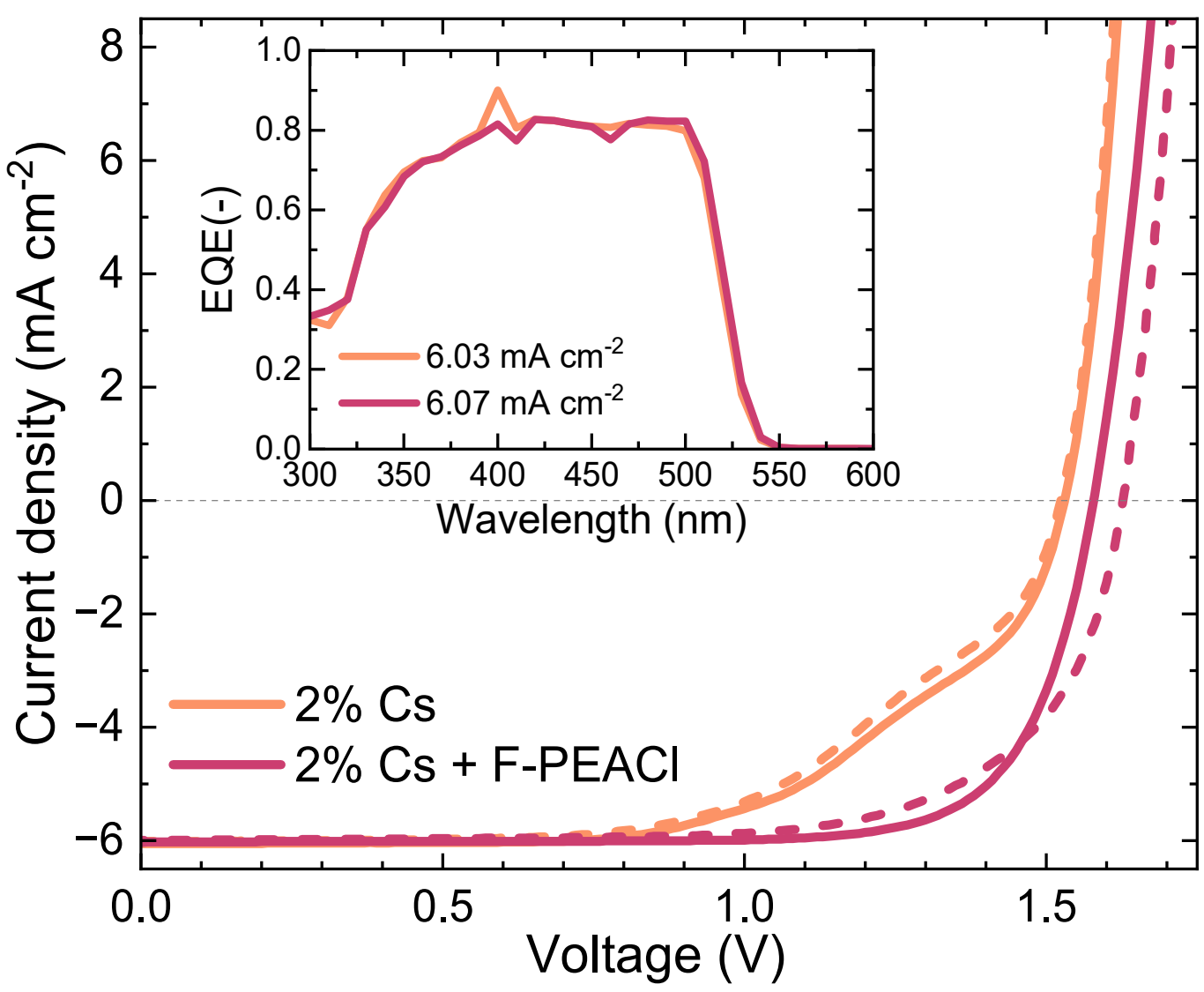

Fig. S30 Current density–voltage (*JV*) characteristics in reverse and forward scan directions of the best performing $Cs_{0.02}FA_{0.98}PbBr_{2.4}Cl_{0.6}$ perovskite solar cells processed without and with 1.0 mg mL^{-1} F-PEACl top surface passivation and PCBM as ETL.

Table S7 Photovoltaic parameters from recorded reverse and forward current density–voltage scans of the best performing $Cs_{0.02}FA_{0.98}PbBr_{2.4}Cl_{0.6}$ perovskite solar cells processed without and with 1.0 mg mL^{-1} F-PEACl top surface passivation and PCBM as ETL.

Sample	Scan	V_{OC} [V]	FF [-]	J_{SC} [mA cm^{-2}][a]	PCE [%]	HI [%]
2% Cs	Reverse	1.53	0.60	6.05	5.5	3
	Forward	1.52	0.58	6.02	5.3	
2% Cs + F-PEACl	Reverse	1.58	0.77	6.03	7.3	5
	Forward	1.63	0.70	6.00	6.9	

[a] From integrating the EQE with the AM1.5G irradiance.

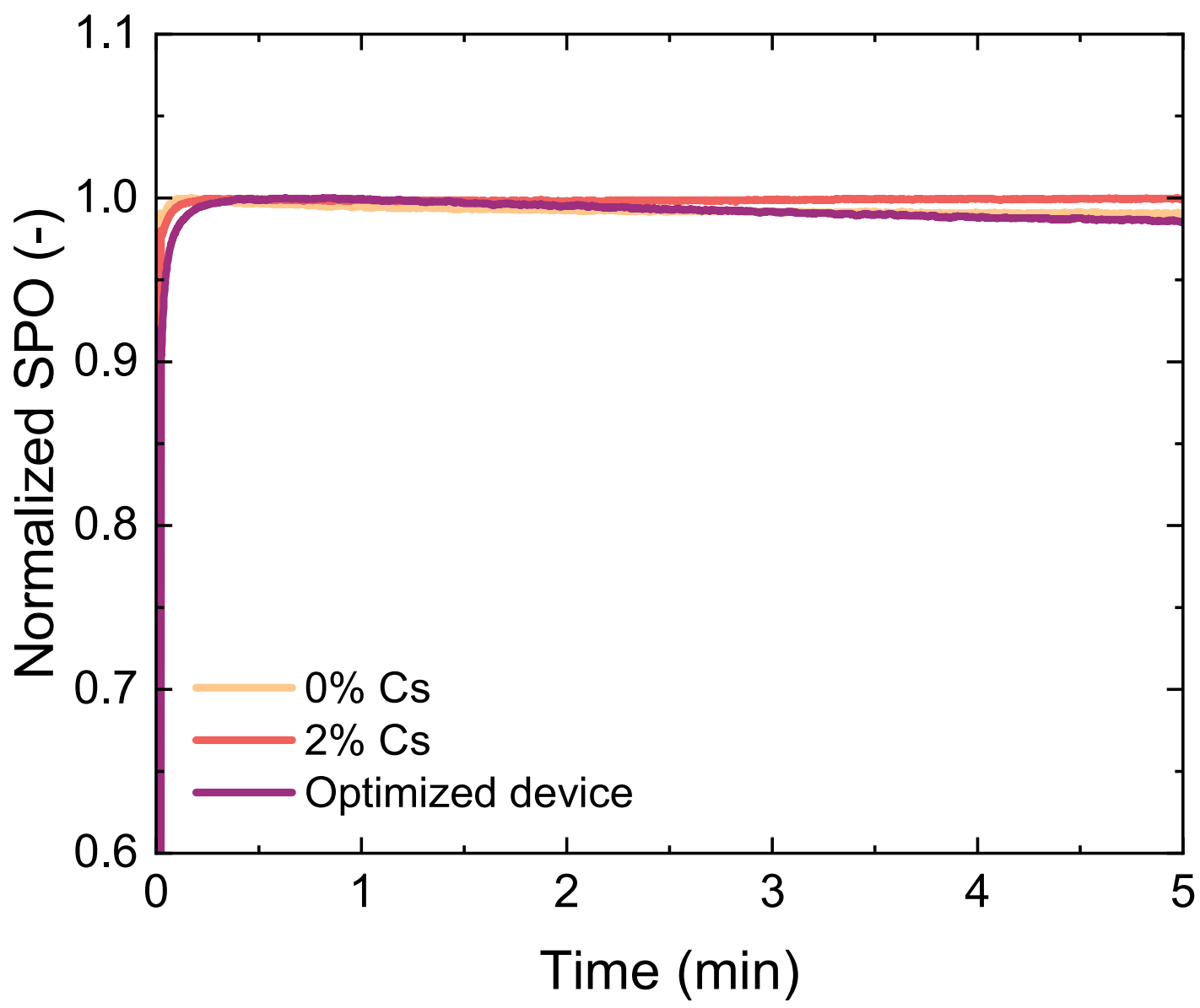

Fig. S31 Normalized stabilized power output of the best performing $Cs_xFA_{1-x}PbBr_{2.4}Cl_{0.6}$ perovskite solar cells processed without (x = 0) and with 2% Cs (x = 0.02) incorporation in the bulk, and of the optimized device processed with F-PEACl top surface passivation and with a 1:1:1 (w/w/w) mixture of PCBM, CMC, and ICBA as ETL.

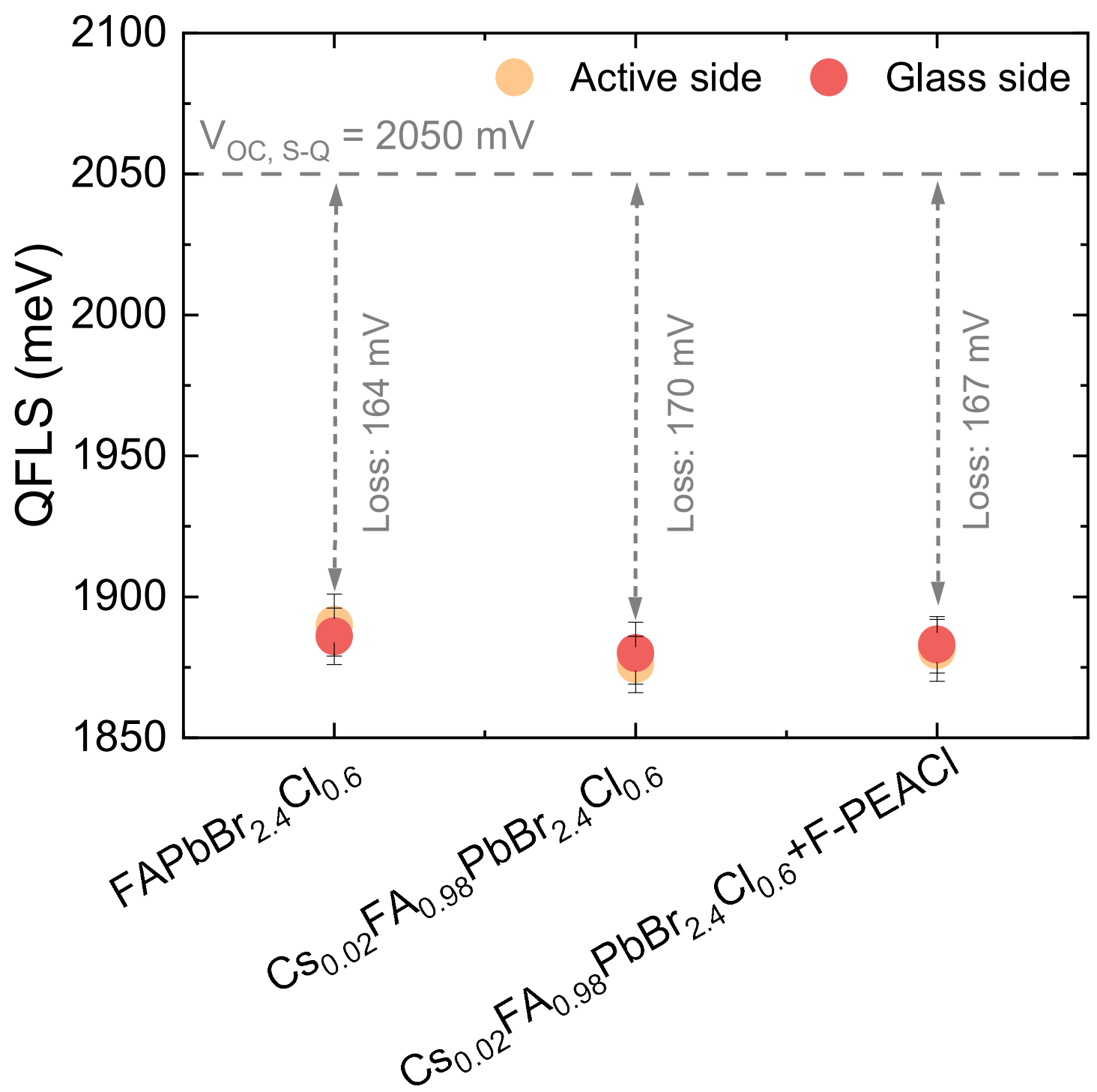

Fig. S32 QFLS of $Cs_xFA_{1-x}PbBr_{2.4}Cl_{0.6}$ perovskite films processed without (x = 0) and with 2% Cs (x = 0.02) and 1.0 mg mL^{-1} F-PEACl surface top passivation.

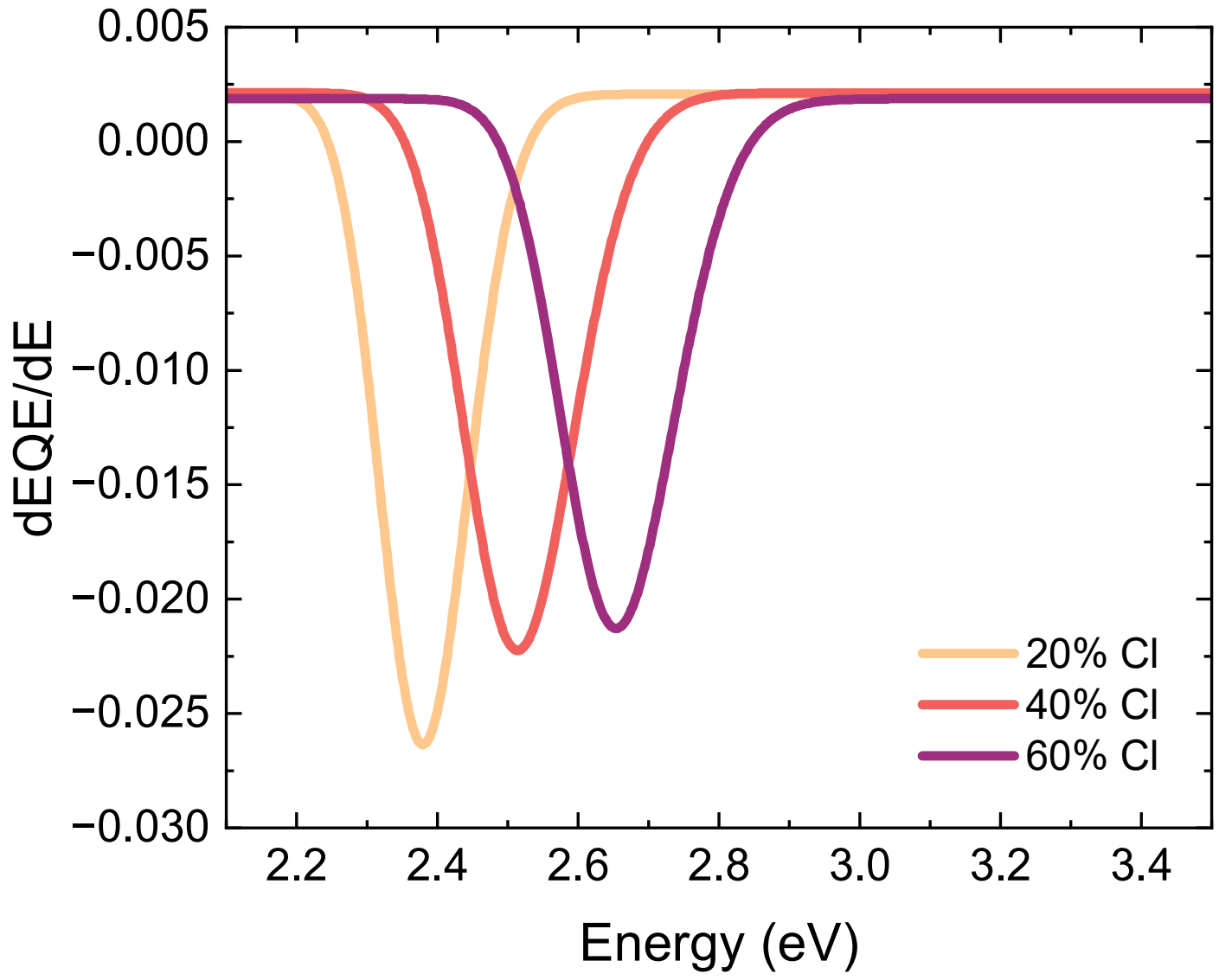

Fig. S33 Bandgap (E_g) values of $FAPbBr_{3-x}Cl_x$ perovskite films containing 20, 40, and 60% Cl relative to the total halide concentration determined from the first derivative of the EQE spectra using Gaussian fitting.

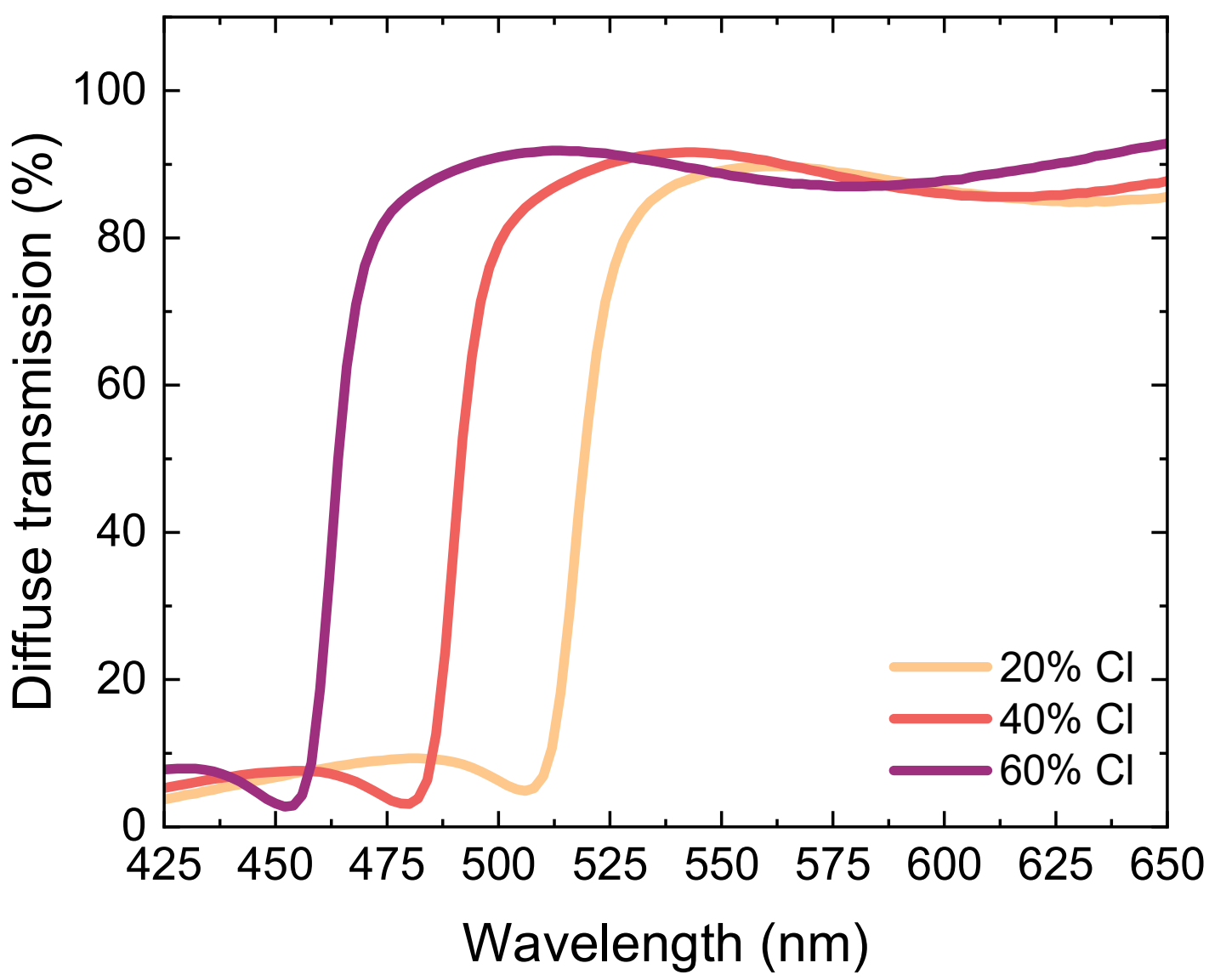

Fig. S34 Diffuse UV-vis transmission spectra of $FAPbBr_{3-x}Cl_x$ perovskite films containing 20, 40, and 60% Cl relative to the total halide concentration.
